# Equipositioning of chromosomes in the polyploid archaeon *Haloferax volcanii* by HpaAB

**DOI:** 10.1038/s41467-026-78993-w

**Published:** 2026-10-30

**Authors:** Kartikeyan Premrajka, Ulrike Johnsen, Holger Preidel, Jascha Wienberg, Chiara Corleis, Marius Ortjohann, Giacomo Giacomelli, Marc Bramkamp

**Affiliations:** Institute for General Microbiology, Kiel University, Kiel, Germany

**Keywords:** Archaeal biology, Archaeal genetics, Cellular microbiology, Chromosome segregation

## Abstract

Proper genome segregation is essential for cellular survival, yet this process is poorly understood in archaea. Archaea show considerable variability in genome ploidy and organization. *Haloferax volcanii* DS2 is a polyploid archaeon with a fragmented genome consisting of a main chromosome, three secondary chromosomes and a plasmid. Due to high ploidy, genomic segregation in *H. volcanii* is typically considered random. However, we identified four two-gene operons encoding ParA-like proteins and uncharacterized partners. Here we characterized HpaA and HpaB, encoded on the secondary chromosome pHV3. HpaA contains conserved ATPase walker motifs and is a ParA protein with a bacterial origin. Mutation of key residues in HpaA alters its localization, while HpaA is necessary for proper HpaB spatial distribution. HpaB forms cytosolic foci and functions as a centromere-binding protein, analogous to bacterial ParB. Deletion of the genes *hpaA and hpaB* results in severe growth defects when xylose is the sole carbon source, consistent with the location of xylose degradation genes on pHV3. It also reduces the pHV3 to main chromosome ratio. Single-particle tracking suggests that HpaB influences HpaA dynamics. Together, these findings demonstrate an equipositioning of pHV3, likely supporting maintenance in the different cell morphologies observed in *H. volcanii*.

## Abstract (web summary)

How the archaeon *Haloferax volcanii* segregates its multi-copy chromosomes during cell division is unclear. Here, the authors identify two proteins required for an equidistant positioning mechanism, maintaining uniform spacing between chromosomes.

## Introduction

Proper segregation of the genome is a fundamental process across all domains of life, ensuring the accurate transmission of genetic material to progeny during cell division. While this process has been extensively characterized in both Eukarya^1,2^ and Bacteria^3–6^, our understanding of genome segregation in Archaea remains limited. In bacteria, one of the most comprehensively studied DNA segregation systems is the ParABS system. Originally identified in plasmid partitioning^3,7–11^, it was later found to also function in chromosomal segregation across diverse bacterial species^4,12,13^. The system comprises three core components: ParA, an ATPase; ParB, a site-specific DNA-binding protein; and *parS*, a centromere-like DNA sequence^12,14^. ParB recognizes *parS* sites that are typically clustered around the origin of replication (*oriC*)—through its CTP-binding and DNA-binding domains^12,15–18^. ParB forms dimers with high affinity for *parS*^4,12^, followed by CTP binding of the *parS-*bound ParB^19,20^, which allows for clamping of ParB onto the DNA and liberates it from *parS*, allowing spreading onto proximal DNA^21^. Subsequent CTP hydrolysis triggers unclamping and release of ParB from DNA, allowing for recycling and maintaining a dynamic pool of ParB molecules around *oriC*^22^. This CTPase activity is essential for forming the partitioning complex^17,19,20^. Additionally, ParB has been shown to be capable of CTP-dependent liquid-liquid phase separation (LLPS), creating condensates that concentrate ParB at *parS* sites. ParA-ATP-driven interactions allow for positioning or splitting these condensates, in turn supporting efficient segregation^23–25^.

ParA, a Walker-type ATPase, binds non-specifically to the nucleoid DNA in its ATP-bound dimeric form^26–29^. Interaction with *parS*-bound ParB triggers ATP hydrolysis, locally releasing ParA from the DNA and generating a dynamic ParA distribution^30^. The resulting spatial asymmetry of ParA drives a diffusion-ratchet mechanism, whereby ParB-*parS* complexes move toward regions enriched in DNA-bound ParA^30–34^, producing directional segregation and bipolar positioning of *parS*-containing genetic elements. The ParABS system has been characterized in a variety of bacteria, including *Bacillus subtilis*^35^, *Corynebacterium glutamicum*^36^, *Caulobacter crescentus*^30,37^ and *Myxococcus xanthus*^13^.

Interestingly, the ParA family of ATPases also contains multiple proteins that are involved in cargo positioning or spatial regulation. For instance, gradient-based mechanisms allow distribution of cargo within the cell, such as carboxysome positioning via McdA oscillation^38^. On the other hand, division site positioning is regulated by MinD gradients in *E. coli*^39^, while a bipolar gradient is formed by MipZ^40^ in alpha proteobacteria like *Caulobacter*. Interestingly, while ParB in some of these systems is responsible for stimulating the ATP hydrolysis of ParA, this interaction promotes dimerization of MipZ instead, leading to a dynamically different gradient of MipZ as compared to ParA^40^.

In contrast to bacteria, the mechanisms of DNA segregation in most archaeal lineages remain poorly understood. Some progress has been made in model crenarchaeal species such as *Sulfolobus solfataricus* and *Sulfolobus* strain NOB8H2. Two systems, SegAB^41,42^ and AspA-ParBA^43^, have been implicated in chromosome and plasmid segregation in these organisms, respectively. The AspA-ParBA system, encoded on the pNOB8 plasmid of *Sulfolobus NOB8H2*, features components with varying degrees of similarity to bacterial homologs. The ParB and ParA proteins share 42–58% and 33–37% sequence identity with their bacterial counterparts, respectively^44^. However, AspA is unique to Archaea and contains a helix-turn-helix motif. AspA binds specifically to a 23 bp palindromic sequence upstream of the segregation cassette and spreads along adjacent DNA at high concentrations, forming a DNA-protein superhelix^43^. ParB in this system functions as a bridging adapter between AspA and ParA. It has a modular structure, with the N-terminal domain binding AspA, the C-terminal domain binding non-specifically to DNA, and a linker region responsible for ParA interaction. ParA, like its bacterial counterpart, binds DNA in an ATP-dependent manner. The *aspA*-*parB*-*parA* cassette is widespread among Crenarchaeota, appearing on both plasmids and chromosomes^43^.

Archaea are a genetically diverse domain, and cell division mechanisms, genome ploidy, and organization vary widely among its lineages^45^. For example, Euryarchaeota have been shown to be oligoploid^46,47^, while Crenarchaea have one or two copies of their chromosomes in different stages of the cell cycle^48,49^. One major challenge in characterizing archaeal DNA segregation stems from their varied ploidy. For instance, the halophilic archaeon *Haloferax volcanii* contains approximately 20 chromosomal copies during exponential growth and 12 in stationary phase^50^, along with secondary chromosomes^51^. High ploidy in Euryarchaeota coincides with the presence of histones^52^, including in *H. volcanii*^53^. In *H. volcanii* strain DS2, the genome comprises a 2.8 Mb main chromosome, three secondary chromosomes pHV4 (636 kb), pHV3 (438 kb), and pHV1 (85.1 kb), and the pHV2 plasmid (6.35 kb)^54,55^. All secondary chromosomes contain a single origin of replication, while the main chromosome contains three origins^46,54,56^. However, in the laboratory strain H26 used in this study, pHV2 has been cured^57,58^, and pHV4 is stably integrated into the chromosome due to a fusion event^46^. It is generally believed that *H. volcanii* relies on random distribution rather than an active segregation mechanism for its chromosome, due to its high copy number and lack of identifiable centromere-like structures^59^. However, the segregation of secondary chromosomes and plasmids, which are maintained roughly in equimolar ratios with the main chromosome (0.7:1 to 1.4:1)^55,60^, remains poorly understood.

In this context, we identified several ParA-like genes throughout the *H. volcanii* genome, all organized in putative operons with adjacent uncharacterized genes. One such operon, located on the secondary chromosome pHV3, contains genes HVO_B0018 (Haloarchaeal Partitioning protein A - HpaA) and HVO_B0017 (Haloarchaeal Partitioning protein B - HpaB). While HpaA contains the conserved deviant Walker motif, which is a hallmark of canonical ParA/MinD like ATPases^61^, and the operon organization strongly resembles those of previously described *parABS* systems, HpaB shows no sequence similarity to conventional ParB proteins.

Given the domain structure and gene organization, along with the established role of ParA-family proteins in cargo positioning^62^, we hypothesized that HpaA-HpaB constitutes a novel chromosome segregation system in *H. volcanii* in which a ParA-like protein relies on a novel class of chromosomal ParB functional homologs. We present evidence that the HpaAB system operates as an equidistant positioning mechanism, maintaining uniform spacing between chromosomes. This is mechanistically slightly different from a classical segregation system that allows directional transport of DNA cargo. Moreover, equipositioning would allow effective segregation in both morphologies of *H. volcanii*, since it forms predominantly rods in early growth phases and plates in late growth phases^63^.

The unique physiology of Archaea and the presence of hybrid segregation systems such as AspA-ParBA and the here described HpaAB offer a window into the evolutionary origins of cellular organization, particularly with respect to Horizontal Gene Transfer (HGT). HGT allows for gene exchange between prokaryotes^64^. Haloarchaea, including *H. volcanii*, have acquired a substantial portion of their genome from bacteria^65,66^. Understanding how secondary chromosomes and plasmids are inherited and maintained in such highly polyploid and pleiomorphic organisms is therefore critical. It will not only shed light on genomic stability in complex prokaryotes but also on the evolution of ATPase-based cargo positioning systems across the domains of life.

## Results

### The *hpaAB* operon encodes a ParA-family ATPase and an uncharacterized partner protein

*H. volcanii* H26 contains 4 ORFs encoding a ParA-like protein on its secondary chromosomes and plasmids; two on pHV4 and one each on pHV3 and pHV1 (Table 1). All the ORFs are each arranged in an operon where the genes of annotated ParA-family proteins are immediately upstream of genes of unknown function, with an overlap of 4–8 nucleotides. Moreover, 3 out of the 4 operons are located in the vicinity of the origins of each secondary chromosome, which has been shown to be the case for *parAB* operons in the ParABS systems^67,68^, which is likely due to the vicinity of the *parS* site to the origin^6^. Only pHV4, which has integrated into the main chromosome in the lab strain H26, has two such operons, with the second (HVO_A0458 and HVO_A0459) being distant from the *ori* of pHV4.

**Table 1:** Four open reading frames on the *H. volcanii* genome with a ParA-annotated protein

Genetic element	Gene	Annotation	Size (bp)
pHV3	HVO_B0018 (HpaA)	ParA family protein	865
	HVO_B0017 (HpaB)	Hypothetical protein	424
pHV4	HVO_A0003	ParA family protein	826
	HVO_A0002	Hypothetical protein	580
	HVO_A0458	ParA family protein	919
	HVO_A0459	Hypothetical protein	403
pHV1	HVO_C0017	ParA family protein	931
	HVO_C0018	Hypothetical protein	388

In this project, we focus on the secondary chromosome pHV3, with the operon containing the HVO_B0018 (HpaA) and the HVO_B0017 (HpaB) loci. The operon has an overlap of 4 nucleotides between the genes and is therefore likely a single transcription unit.

The *hpaB* gene is located immediately downstream of *hpaA*. HpaB shows no significant sequence similarity to any annotated or characterized bacterial protein, including ParB. HpaA shows a high degree of similarity (70–90%) with other annotated ParA-like proteins in other Halobacteria, and specifically *Haloferax sp*.

**Fig. 1:**
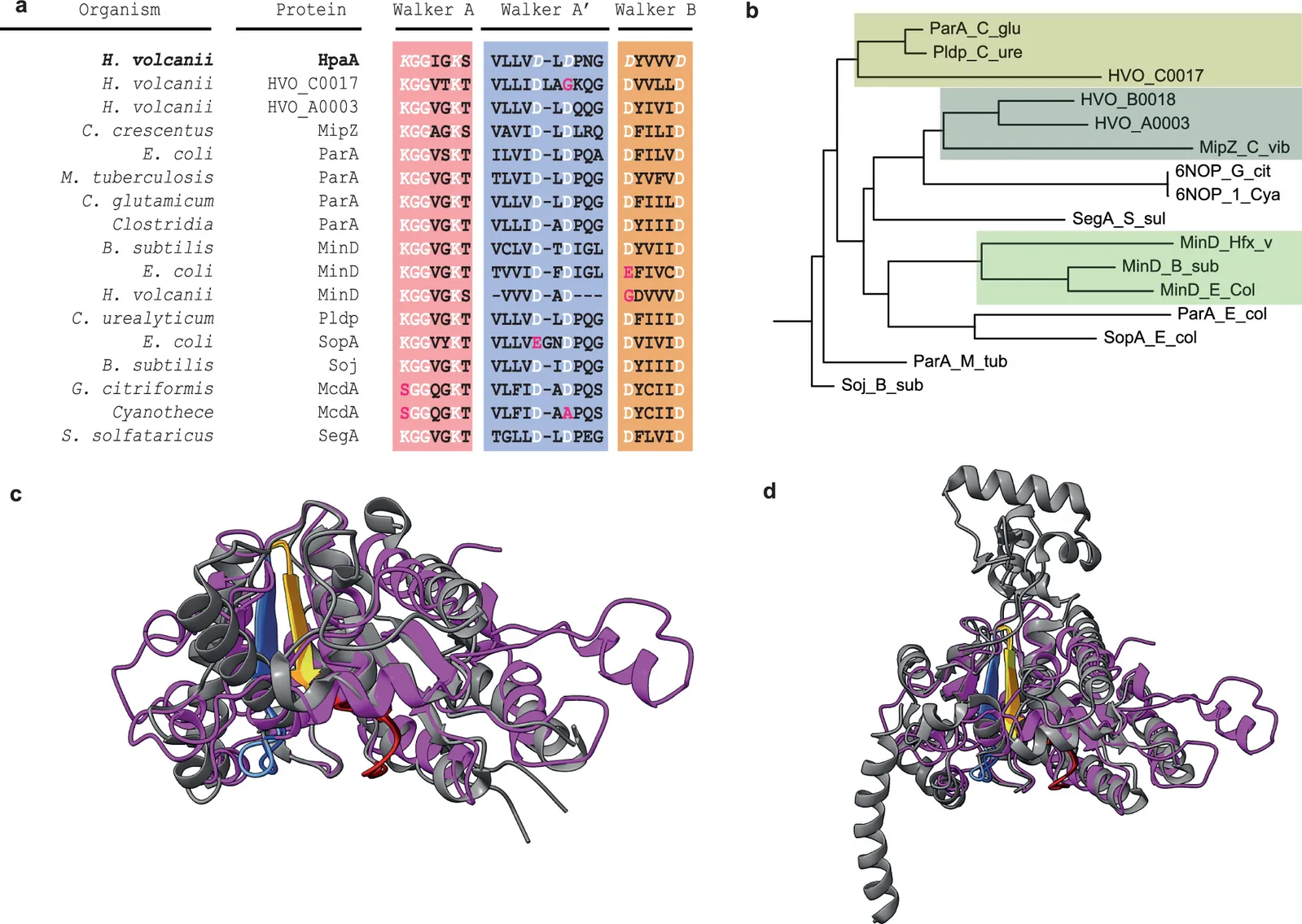
HpaA is a ParA homolog. **a** Alignment of the amino acid sequences of the Walker A, A’, and B motifs conserved among known bacterial and archaeal ParAs. The signature lysine, invariant glycines, catalytic lysine in the Walker A motif, and the catalytic aspartate in the Walker A’ motif are conserved (highlighted in white). **b** Phylogenetic tree using the whole gene sequence of HpaA with other *Haloferax* HpaAs and known bacterial ParAs; made using the neighbor-joining method. Colors indicate closer related proteins by branch length. The predicted structure of the AlphaFold model of the HpaA dimer in magenta, when compared with known structures of MipZ (2XJ4) in (**c**) and ParA (3EZ2) in (**d**) in gray, shows an overlap of the Walker motifs (Red/Dark Red—Walker A, Blue/Turqoise—Walker A’, Orange/Yellow—Walker B). Source data are provided as a Source Data file.

To assess the similarity between HpaA and canonical ParA proteins, a multiple sequence alignment was performed using the HpaA sequence and characterized ParA family proteins from *Escherichia coli*, *Bacillus subtilis*, *Corynebacterium glutamicum*, *Mycobacterium tuberculosis*, and *Clostridium Sp*. Additional comparisons included ParA-related proteins such as MinD, MipZ, and McdA from *Cyanothecae*, as well as SegA from the archaeon *Sulfolobus*. HpaA shows a high degree of similarity with the conserved ATPase Walker motifs of canonical ParA family proteins. This is corroborated by the presence of conserved key residues in the Walker A region, Walker A‘ region, and Walker B region; especially the signature lysine, catalytic lysine, and invariant glycine in Walker A (Fig. 1a).

A phylogenetic tree constructed using the neighbor-joining method with the aforementioned protein sequences categorized the ParA family proteins into three distinct clusters (Fig. 1b). The clusters were determined using phylogenetic distances-dependent branch lengths. Clustering of similar proteins like MinDs and canonical ParAs indicated the robustness of the tree. In the calculated tree, HpaA clustered with the MipZ protein from *Caulobacter vibriodes*.

Comparison of the predicted structure of HpaA using Alphafold 3 with known structures of MipZ (pdb: 2XJ4) and ParA (pdb: 3EZ2) showed that HpaA shared a higher structural similarity with MipZ as compared to ParA (Fig. 1c, d). The RSMD scores between the predicted HpaA structure and MipZ were lower at 9.931 Å unpruned and 0.93 pruned; while ParA from *E. coli* had higher RSMD scores at 10.416 Å unpruned and 0.99 Angstroms pruned. However, in both cases, the conserved ATPase Walker motifs overlapped to a high degree.

### HpaA binds DNA in the presence of ATP

**Fig. 2:**
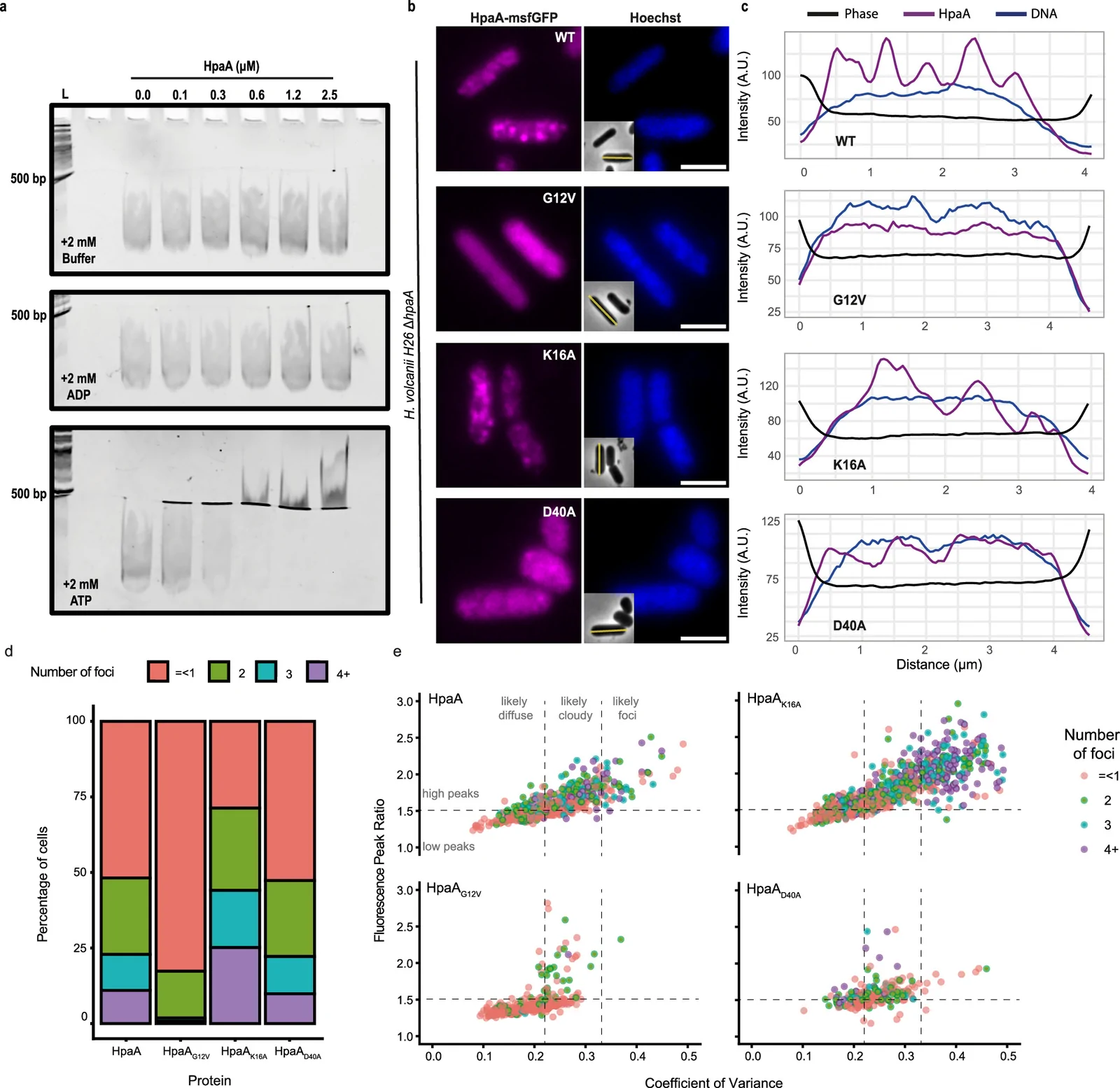
DNA-binding and in vivo behavior of HpaA depend on the ATP Walker motif. **a** Electrophoretic mobility shift assays (EMSAs) of dsDNA fragments incubated with increasing concentrations of HpaA and 2 mM nucleotide. **b** Fluorescence microscopy showing representative cells at OD_600_ of 0.02, with different in vivo behavior of HpaA tagged with msfGFP (magenta) in H26 Δ*hpaA H. volcanii*. DNA is stained with Hoechst dye (blue). Scale bar = 2 µm. Yellow line across inset indicates line scan area for (**c**), which shows presence or absence of foci across the cell length in relation to DNA (Blue). **d** Differences in number of foci observed between different HpaA variants as a percent of different foci numbers in each population. **e** Scatter plot of coefficient of variance (CV) vs. fluorescence peak ratio (FPR) for each cell, plotted with color showing foci number in that cell. Dotted gray line indicates cut-offs to determine diffusive, cloudy, foci-forming behavior, respectively, and is compared between mutants. *n* (HpaA_WT_ = 629, HpaA_G12V_ = 385, HpaA_K16A_ = 909, HpaA_D40A_ = 303) for each strain from three replicates. Source data are provided as a Source Data file.

ParA-like proteins such as ParF, Soj, MipZ, and ParA form dimer sandwiches that trap nucleotides for hydrolysis, with the signature lysine of each monomer inserted into the active site of the other^28,69–71^. McdA forms a similar dimer sandwich without the signature lysine and rather relies on an McdA-specific conserved lysine^72^. On the other hand, SegA forms a forward-backward dimer rather than adopting an ATP sandwich^42^. Structure prediction of dimeric HpaA in the presence of ATP via Alphafold 3 shows ATP sandwiched within the dimer interface with the signature lysine (K11) predicted to interact with the ATP of the other subunit, similar to canonical ParAs described above (Supplementary Fig. 1a). Moreover, the surface of the predicted HpaA dimer is largely negative owing to the high salt concentration of *H. volcanii* cytoplasm^73^. However, it contains a positively charged cleft (Supplementary Fig. 1b, c) which is predicted to be the DNA-binding region. Electrophoretic mobility shift assays with purified HpaA revealed that HpaA binds DNA in the presence of ATP (Fig. 2a), with progressively stronger shifts observed at increasing protein concentrations. In contrast, no detectable DNA binding by HpaA was observed in the absence of ATP or in the presence of ADP, as indicated by the absence of shifted DNA bands (Fig. 2a). These results suggest that HpaA DNA binding is ATP-dependent.

### Dynamic localization of HpaA depends on its putative conserved ATPase cycle residues

As fundamental insights toward the understanding of canonical ParABS systems can be collected via the study of their subcellular localization, we proceeded to tag HpaA with msfGFP followed by in trans expression in *H. volcanii* H26 Δ*hpaA*. HpaA-msfGFP variants showed different levels of heterogeneity in cells. HpaA-msfGFP was observed to form multiple cloudy patches and foci along the cell (Fig. 2a), along with a cloudy, dispersed population throughout the cell. The foci did not colocalize with any compacted DNA as seen by the line scans. In some cases, HpaA-msfGFP also showed oscillatory behavior (Supplementary Fig. 2a). This was predominantly observed in rod-shaped cells.

Subcellular dynamics of ParA-like proteins depend on their ATPase cycles. The Walker sites harbor highly conserved amino acid residues, which are found in both bacterial ParA proteins and HpaA (Fig. 1a). These residues are essential for the ATP-dependent functions of dimer-sandwich-forming ParA-like proteins^70,74,75^. Any variations in these amino acids disrupt ATP binding, hydrolysis, or dimer formation, ultimately impairing the protein’s localization and function in vivo. Strategic substitutions were introduced in the conserved key residues of HpaA that would lead to HpaA being trapped in different stages of its mechanistic cycle. The invariant glycine was replaced with a valine at position 12 (G12V), the catalytic lysine with alanine at position 16 (K16A), and the aspartate at position 40 with alanine (D40A). These mutations have been previously described for a variety of ParA-like proteins^29,40,70,71^.

All HpaA mutants were expressed in a strain background lacking the wild-type allele of HpaA to prevent heterodimer formation. The mutants displayed distinct in vivo behaviors that could be quantitatively assessed. The distribution of foci number varied among the variants, with G12V showing the lowest proportion of cells containing foci, whereas K16A exhibited a higher proportion of cells with foci and a greater average number of foci per cell.

We also quantified signal heterogeneity for each variant using scatter plots of the coefficient of variance (CV) and fluorescence peak ratio (FPR). Higher CV and FPR values indicated more defined and stable foci, whereas lower CV values corresponded to increasingly cloudy or near-diffuse protein distributions. K16A showed a greater tendency to form strong foci, while D40A exhibited a more diffuse, cloudy distribution. In contrast, G12V displayed an almost completely diffuse localization pattern. In most cases, high CV and FPR values also correlated with cells containing a larger number of foci. Statistically, G12V showed the greatest deviation from the wild-type distribution, whereas D40A most closely resembled the wild type (Supplementary Fig. 3).

None of the mutants exhibited oscillatory behavior (Supplementary Fig. 2a, b). The kymographs show alternating enrichment of signal at opposite poles for full-length HpaA-msfGFP. On the other hand, all variants lack this behavior (Supplementary Fig. 2b). HpaA-msfGFP G12V exhibited a nearly homogeneous fluorescence distribution, HpaA-msfGFP K16A displayed a patchy localization pattern due to foci-like behavior, and HpaA-msfGFP D40A showed a cloudier signal with increasing fluorescence toward the center of the cell.

The distinct in vivo localization behaviors of the HpaA variants highlight the importance of the conserved residues within the Walker motifs and provide strong cell biological evidence that HpaA functions as an ATPase, similar to bacterial ParA-like proteins.

### HpaA localization and dynamics are dependent on HpaB

It is known that ParA-like protein dynamics, and in certain cases intracellular localization, are dependent on the activity of ParB or non-homologous cargo adapters^74,76–78^. We aimed to determine whether HpaA localization and dynamics also depend on the presence of HpaB. To this end, *hpaA-msfGFP* and *hpaA-HaloTag* were ectopically expressed in *H. volcanii* H26 strains, wild-type and *H. volcanii* H26 Δ*hpaB*. HpaA-msfGFP was used to qualitatively describe the dynamics of HpaA, while the HaloTag-tagged protein was constructed to study the effects of HpaB on HpaA at the single-molecule level using organic dyes. HpaA and HpaB were also tagged with mTurquoise2 and mScarletI^79^, respectively, for dual fluorescent expression and co-localization studies.

**Fig. 3:**
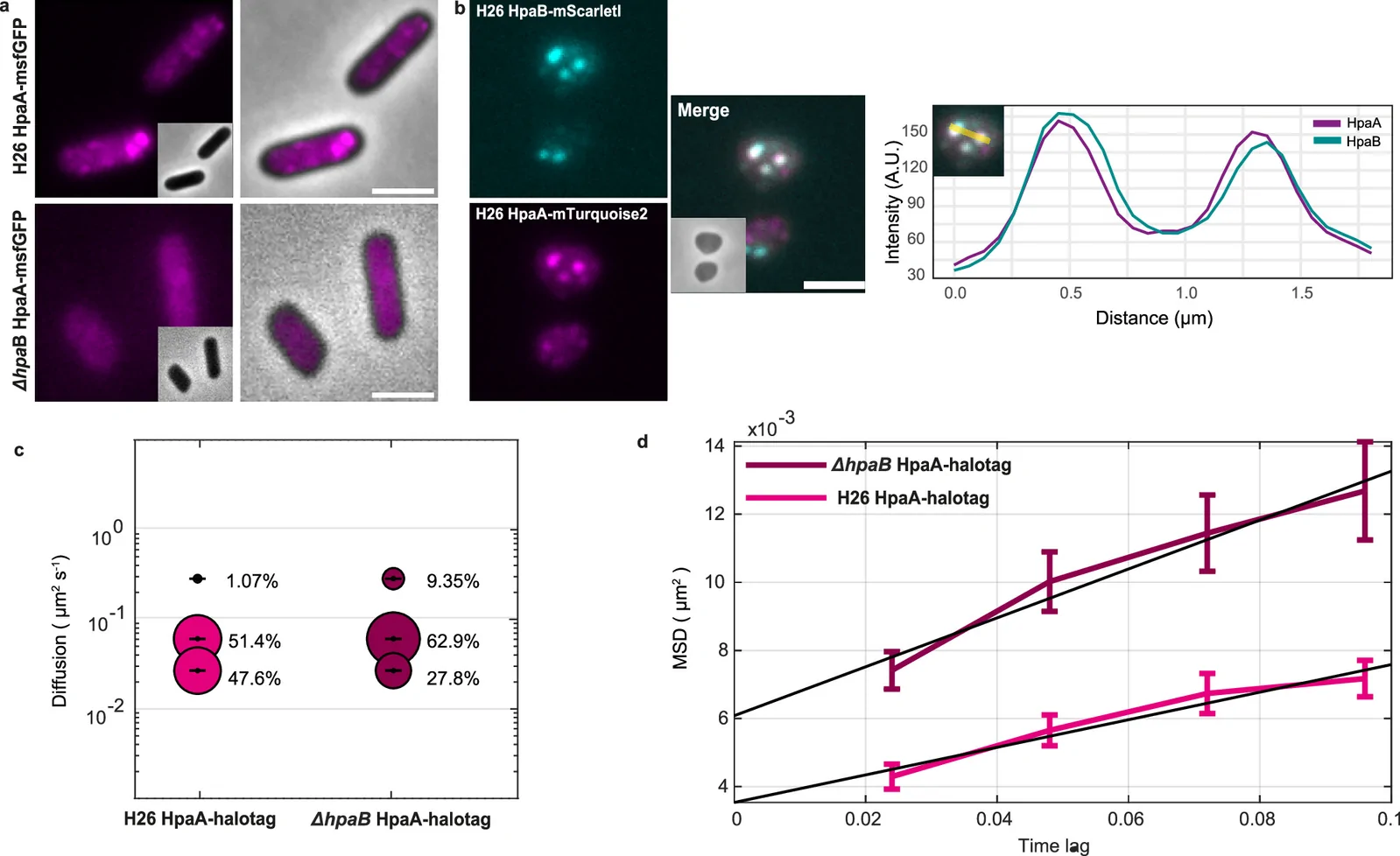
In vivo localization and dynamics of HpaA depend on HpaB. **a** Fluorescence microscopy showing representative cells at OD_600_ of 0.02 with HpaA tagged with msfGFP (Magenta) in wild-type cells and cells lacking *hpaB*. Scale bar = 2 µm. **b** Colocalization of HpaA-mTurquoise2 (magenta) and HpaB-mScarletI (Cyan) foci in representative cells OD_600_ of 1.0 along with a representative line scan showing the overlapping peaks of foci (yellow line in inset shows selected area of interest). Scale bar = 1 µm. **c**, **d** show dynamics of single particles of TMR stained HpaA-HaloTag in *H. volcanii* H26 *WT* and *ΔhpaB* using SMTracker (Rösch et al.^121^). **c** Bubble plot showing single-molecule diffusion rates of HpaA-HaloTag in wild type (*n* = 8745 tracks) and mutant cells (*n* = 21,615 tracks). Populations were determined by fitting the cumulative distribution function (CDF) of square displacements with a three-component model (‘fast’, ‘slow’, and ‘static’ protein populations). **d** Plot of the mean-squared displacement of HpaA-HaloTag in wild type (*n* = 8745 tracks) and mutant cells (*n* = 21,615 tracks). Data are presented as mean values of the MSD with error bars depicting standard deviation.

Fluorescence images show that a majority of the wild-type cells in the logarithmic stage of growth display a heterogeneous distribution of HpaA-msfGFP all over the cell. In most cases, HpaA additionally tends to form foci-like structures (Figs. 2 and 3a). On the other hand, in the absence of HpaB, the fluorescence of HpaA-msfGFP is more homogenously distributed in the cells, and discrete foci were rarely observed (Fig. 3a). Moreover, the oscillatory behavior of HpaA seemed to be very obvious in H26, and abolished in absence of HpaB (Supplementary Fig. 4). Interestingly, instead of being continuous, the oscillation from one pole to the other in rod cells was observed within 1–2 min followed by persistence of signal at the new pole for more than 5 min. The plate cells showed a complete absence of oscillation of HpaA in both wild-type and mutant strains.

Upon high induction of fluorescently tagged HpaA and HpaB, foci from both proteins were observed to often localize in close proximity (Fig. 3b). The presence of foci with non-obvious colocalization can be attributed to the wild-type background of the strain, expressing the native copies of both HpaA and HpaB. A representative line scan of foci from each channel (HpaA-mTq2 and HpaB-mSC-I) shows that there is enrichment of HpaB and HpaA around the other foci as well. Combined with the observation that HpaA foci formation requires the presence of HpaB, the colocalization between HpaA and HpaB supports a model in which HpaB causes a local enrichment of HpaA (Fig. 3b).

To further investigate the effect of HpaB on HpaA dynamics and localization, single-particle tracking (SPT) was performed using single-molecule localization microscopy (SMLM). Therefore, ectopically expressed HpaA-HaloTag was stained with the TMR fluorescent dye to enable precise single-molecule detection and tracking within live cells. SPT measurements used a 20 ms exposure time.

Comparison of single-molecule dynamics showed that HpaA was more mobile in the absence of HpaB as compared to the wild-type cells (Fig. 3c, d). Protein dynamics are comprised of multiple separate subpopulations, each defined by its own parameters. To account for this behavior of dynamic proteins, jump distance (JD) analysis was performed. The data could be interpreted best as three distinct populations, which were identified and are represented as bubble plots (‘fast’, ‘slow’ and ‘static’ from top to bottom) in both strains (Fig. 3c). The fast-mobile proportion of the protein was small to begin with, at 1.07%. However, the proportion of fast-mobile protein in the Δ*hpaB* strain shows a huge increase to 9.35%. Moreover, an increase in the slow-mobile fraction of the protein can also be observed from 51.4% in the wild type to 62.9% in the mutant. The increase in both fast and slow mobile populations is mirrored by a corresponding decrease in the static fraction from 47.6% in wild type to 27.8% in Δ*hpaB* cells (Fig. 3c). In light of these results, we hypothesize the three fitted populations of the proteins to be enriched respectively in diffusive monomers (fast), DNA-interacting dimers (slow diffusive), and the segregation complex-associated dimers (static). The distribution of the populations based on their dynamics showed that the absence of HpaB led to an increased mobility of HpaA-HaloTag, indicating that HpaB might have a role in stabilizing the HpaA interaction to the segregation complex. In the absence of HpaB, decreased recruitment of HpaA onto the segregation complex would lead to an accumulation of dimeric HpaA interacting non-specifically with DNA, hence increasing the slow diffusive population and decreasing the static population. Subsequently, saturation of DNA with HpaA would lead to freely diffusive population of HpaA increasing, which can be seen as the highest increase in fast diffusive population. Mean-squared displacement (MSD) was used as a quantitative indicator of the averaged diffusion speed. It showed that the diffusion coefficient of HpaA-HaloTag was larger in absence of HpaB (Fig. 3d). The results are intriguing because HpaA loses its oscillatory behavior, while its dynamicity increases in absence of HpaB. This is probably because of an increase in free cytoplasmic protein, and by extension an increase in random protein diffusion.

### HpaA and HpaB are required for optimal growth on xylose

**Fig. 4:**
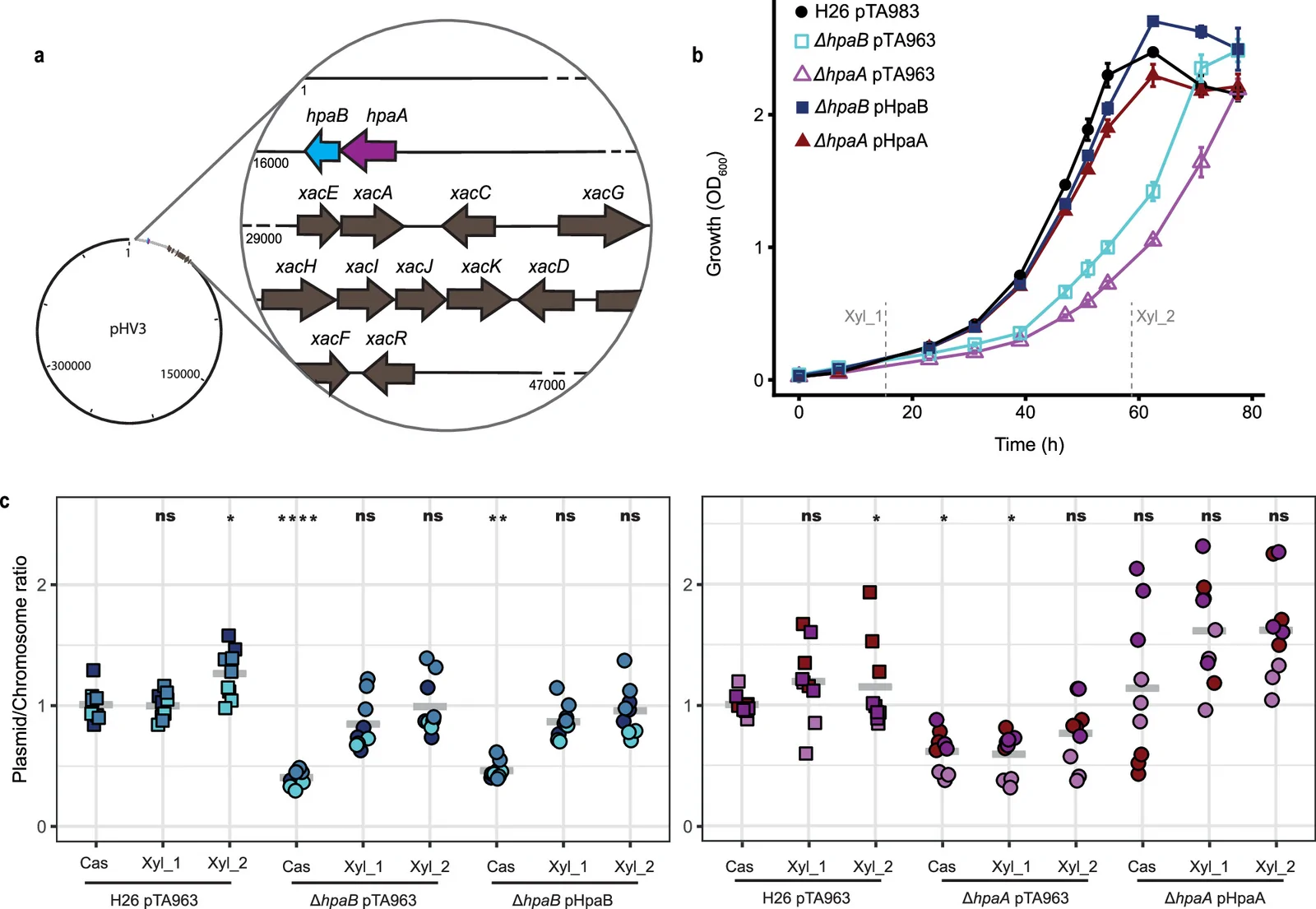
HpaB is essential to maintain pHV3 to chromosome ratio and for growth on xylose. **a** Cartoon showing the position of the *hpaAB* operon and the xylose degradation pathway genes on pHV3. **b** Growth curves of *H. volcanii* H26 wild type, *ΔhpaB, ΔhpaA*, and complement strains on synthetic media containing 20 mM xylose showing a significant defect of growth in the mutants, which is rescued by complementation. All curves were performed in four replicates, data are presented as mean values with error bars depicting standard deviation. **c** qPCR assays performed on genomic DNA of *H. volcanii* grown on casamino acids, and xylose over time, with primers for the main chromosome and pHV3 show pHV3 to chromosome ratio (*y*-axis) for the mutants and complementation strains (*x*-axis). Strains used: H26 *ΔhpaB, ΔhpaA*, wild type, and complement strains induced with 0.1 mM or 0.03 mM tryptophan. Colors indicate biological replicates-with 3 technical replicates each. Stars indicate significance: **** (<0.0001), *** (<0.001), *** (<0.01), *(<0.05), calculated using two-sided Kruskal–Wallis followed by Dunn’s multiple-comparisons test (two sided). Exact significance and effect size values are provided in Supplementary Table 2. Source data are provided as a Source Data file.

A fundamental aspect of chromosomes is that they carry housekeeping and core genes that are usually indispensable for viability and cell fitness. This includes genes involved in uptake and metabolism of carbon sources. The secondary chromosome pHV3 contains all genes that encode proteins involved in xylose uptake and degradation (Fig. 4a). In *H. volcanii*, D-xylose is metabolized via an oxidative degradation pathway to α-ketoglutarate^80,81^. The corresponding enzymes are encoded by the *xac* gene cluster, encoding xylose dehydrogenase (XacA, HVO_B0028), pentonolactonase (XacC, HVO_B0030), D-xylonate dehydratase (XacD, HVO_B0038A), 2-keto-3-deoxy-xylonate dehydratase (XacE, HVO_B0027), and α-ketoglutarate semialdehyde dehydrogenase (XacF, HVO_B0039). Within the same cluster, the genes *xacGHIJK* (HVO_B0034–HVO_B0038) encode an ABC transporter for the uptake of xylose, consisting of a substrate-binding protein (XacG), two transmembrane domains (XacH and XacI), and two nucleotide-binding domains (XacJ and XacK)^82^. The expression of both the catabolic and transporter genes is controlled by the transcriptional activator XacR (HVO_B0040), which induces their transcription in the presence of xylose and arabinose^83^.

We reasoned that the secondary chromosome pHV3 may become in particular important during growth on xylose as a sole carbon source. Consequently, a decrease in pHV3 to chromosome ratio due to loss of inheritance via mis-segregation would lead to a defect in growth on xylose. In line with our hypothesis, deletion of both *hpaA* and *hpaB* resulted in severe growth defects, indicating that both genes are required for robust growth under these conditions (Fig. 4b). Moreover, these defects could be complemented upon tryptophan-controlled expression of HpaA and HpaB via ectopic complementation constructs. Complementation of both *hpaA* and *hpaB* fully restored growth to wild-type levels (Fig. 4b). The transient complementation by HpaA upon ectopic expression was dependent on the intracellular protein concentration, as increasing tryptophan levels resulted in progressively weaker complementation (Supplementary Fig. 5b). These results indicate that the correct concentration of HpaA in the cell is important for full functionality and *hpaA* expression likely requires precise temporal or spatial regulation, for full function during xylose-dependent growth. In contrast to growth on xylose, HpaA or HpaB are not essential for viability of *H. volcanii* H26 when grown on glucose as a carbon source (Supplementary Fig. 5a).

### HpaB is essential to maintain the secondary chromosome pHV3

Chromosomes and extra-chromosomal elements like plasmids are self-replicating entities allowing their vertical inheritance across generations. Inheritance of genomes via ParAB like systems involves and requires specificity, and would need to code for the segregation complex. Organisms with multiple chromosomes such as *Vibrio spp*, and *Deinococcus radiodurans* have *parAB* operons and distinct *parS* sites on their different chromosomes^84,85^. Similarly, low copy number plasmids in bacteria, like the F plasmid or the P1 plasmid in *E. coli*, can be stably maintained with the help of partition systems that are responsible for positioning them inside the cell^86^. In contrast, high copy number plasmids usually do not need an active segregation system because random segregation is sufficient.

To test whether the growth defect on xylose was a direct consequence of reduced pHV3 copy numbers upon deletion of *hpaA* or *hpaB*, we performed a qPCR to obtain a ratio of the secondary chromosome to the main chromosome in a given population. As under physiological conditions, the pHV3 to chromosome ratio ranges between 0.8–1.2, a significant decrease in this ratio upon deletion of the functional HpaAB system would indicate an overall decrease of pHV3 numbers in the population due to a loss of maintenance or stable inheritance. Moreover, improper segregation would result in cells containing fewer or higher copies of pHV3, wherein cells with more pHV3 would be selected for when grown on xylose.

Multiple biological replicates were performed with cultures of the H26 *H. volcanii*, Δ*hpaB*, and Δ*hpaA* strains with either empty vector, or vectors expressing HpaB or HpaA, respectively, to asses pHV3 to chromosome ratios. When grown on casamino acids, the deletion of both genes resulted in a significant decrease in the pHV3 to chromosome ratio (Fig. 4c), from a mean ratio of ~ 1 in the wild type to ~0.35 and ~0.6 in the Δ*hpaB* and Δ*hpaA* mutants, respectively. Upon growth on xylose, samples were taken at two time points: at early log phase, and at late log/stationary phase to determine how the chromosome ratio changed. We observed that the pHV3 to main chromosome ratio significantly increased to near wild type levels earlier for the Δ*hpaB* population and later for the Δ*hpaA* population. The ratio did not seem to significantly increase further in these conditions.

Furthermore, we constructed complementation strains in which *hpaB* and *hpaA* were expressed from a plasmid under control of a tryptophan inducible promoter, respectively. Synthesis of HpaA or HpaB was induced with 0.03 mM, and 0.1 mM tryptophan, respectively. Upon ectopic expression, both proteins seem to at least partially complement the pHV3 to main chromosome ratio. While this is not completely complemented for HpaB, the robustness of the ratio upon HpaA overexpression is lost. Further upon growth on xylose, the HpaB complementation strain reached near wild type levels of pHV3 to chromosome ratio while HpaA complementation dramatically increased the ratio in a less robust fashion. Overall, qPCR results indicated that both HpaA and HpaB are required to maintain pHV3 to chromosome ratio in *H. volcanii*, and that cells with normal pHV3 levels are selected for when grown on xylose.

### HpaB binds specifically to pHV3

Adapter proteins such as ParB, or SegB show highly specific binding sites to their respective chromosomes^18,41^. Combining the fact that the loss of HpaB or HpaA led a reduction in pHV3 numbers in the population, we hypothesized that it binds specifically to pHV3.

**Fig. 5:**
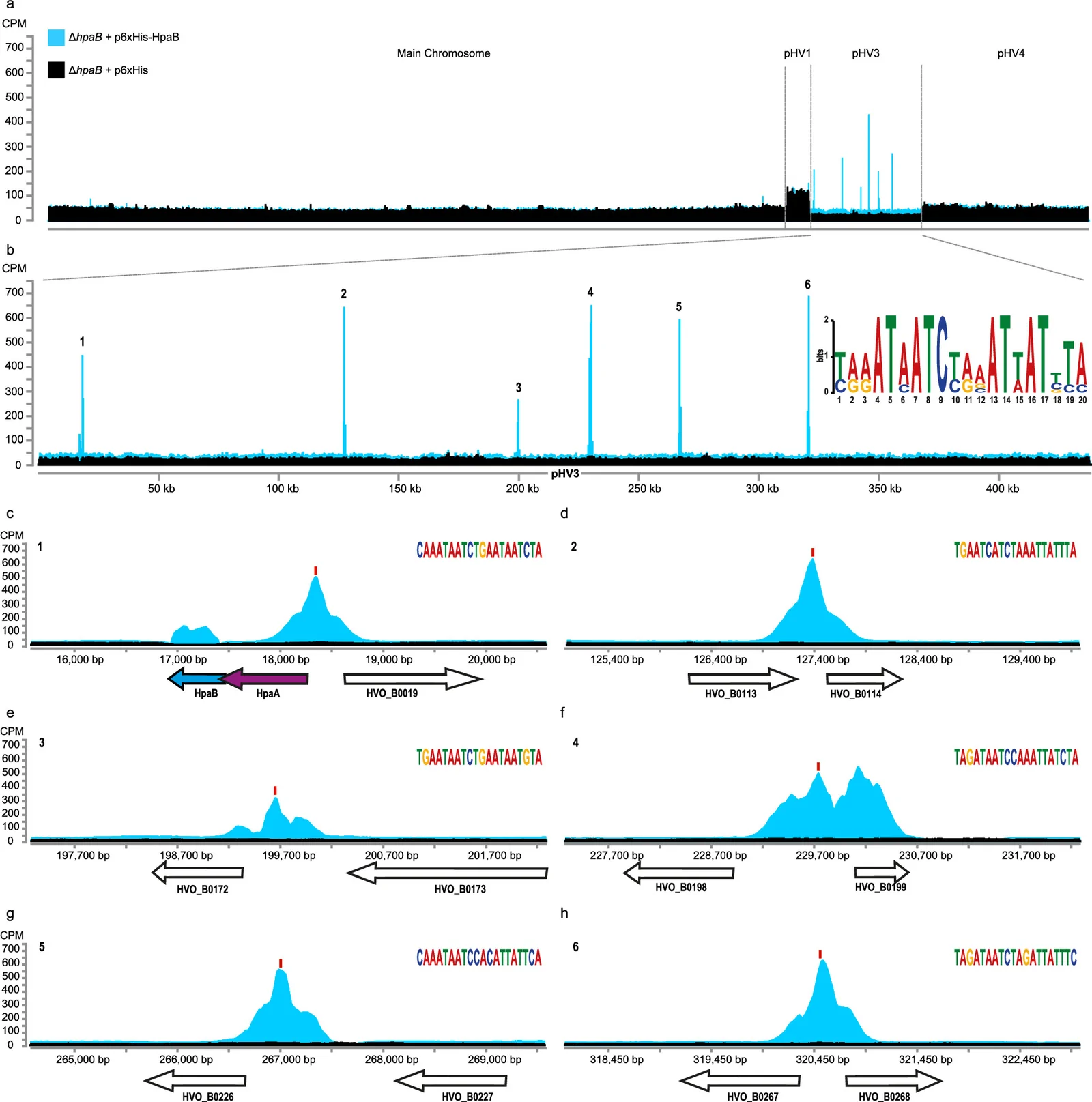
HpaB binds specifically to pHV3 at 6 discreet peaks. **a** ChIP-seq profile of HpaB enrichment on *H. volcanii* genome (presence of HpaB in cyan, absence of HpaB in black). All replicons are labeled. **b** Zoomed-in enrichment of HpaB on pHV3 with each discreet peak labeled from 1–6. The inset shows the consensus sequence for the *hpaS3* motif found on all peaks. **c**–**h** Zoomed-in individual peaks showing their genome coordinates, their spread on pHV3, the motif location to scale (red), motif in inset, and surrounding genes under the *X*-axis. *Y* axis represents counts per million of all bases read (CPM) at each genomic position in all graphs.

To determine the chromosomal binding landscape of HpaB, we performed chromatin immunoprecipitation (ChIP) on exponentially growing *H. volcanii* Δ*hpaB* with a plasmid overexpressing 6xHis-HpaB. *H. volcanii* Δ*hpaB* 6xHis tag (empty plasmid vector) was used as a control. Genome-wide analysis of enriched regions identified six discrete and highly significant peaks located exclusively on pHV3. No comparable enrichment was detected on the remaining replicons, suggesting a pHV3-specific association of the protein (Fig. 5, cyan). Moreover, the peaks were also absent in the control (Fig. 5, black). Motif discovery analysis revealed an AT rich consensus sequence (Fig. 5, panel 2 inset), present exclusively on or right beside the center of the six peaks (Fig. 5, red), except peak 4 where the primary peak contains and secondary peak lacks the motif. The conservation of this sequence across the peaks supports its role as the *cis* element required for protein nucleation. We propose this consensus sequence as the *hpaS3* sequence.

Furthermore, closer inspection of the 6 peaks determined that HpaB has a very weak, if any, spreading pattern from the epicenter of the peaks. Peak enrichment is observed ~800 bps in both directions. Upon investigating the genomic context of the identified binding sites, we observed that while all binding sites were upstream of coding regions, the genes were all unrelated to each other (Supplementary Table 1).

The results not only identify a potential *hpaS* site on *H. volcanii*, they also clearly indicate that HpaB is a DNA-binding protein highly-specific to pHV3, consistent with the growth and qPCR data.

### HpaB forms foci that are ordered in an HpaA-dependent manner

Most ParAB-like systems involved in cargo positioning exist in operons containing an adapter protein^87,88^. This adapter binds to the cargo; for example, centromere binding proteins (CBPs) like ParB bind to the *parS*/*parC* sites on chromosomes or plasmids^89,90^, and McdB binds to carboxysomes^38,88,91^. CBPs are known to form condensed structures to allow high concentration of the protein around their cargo sites^30,91–93^.

**Fig. 6:**
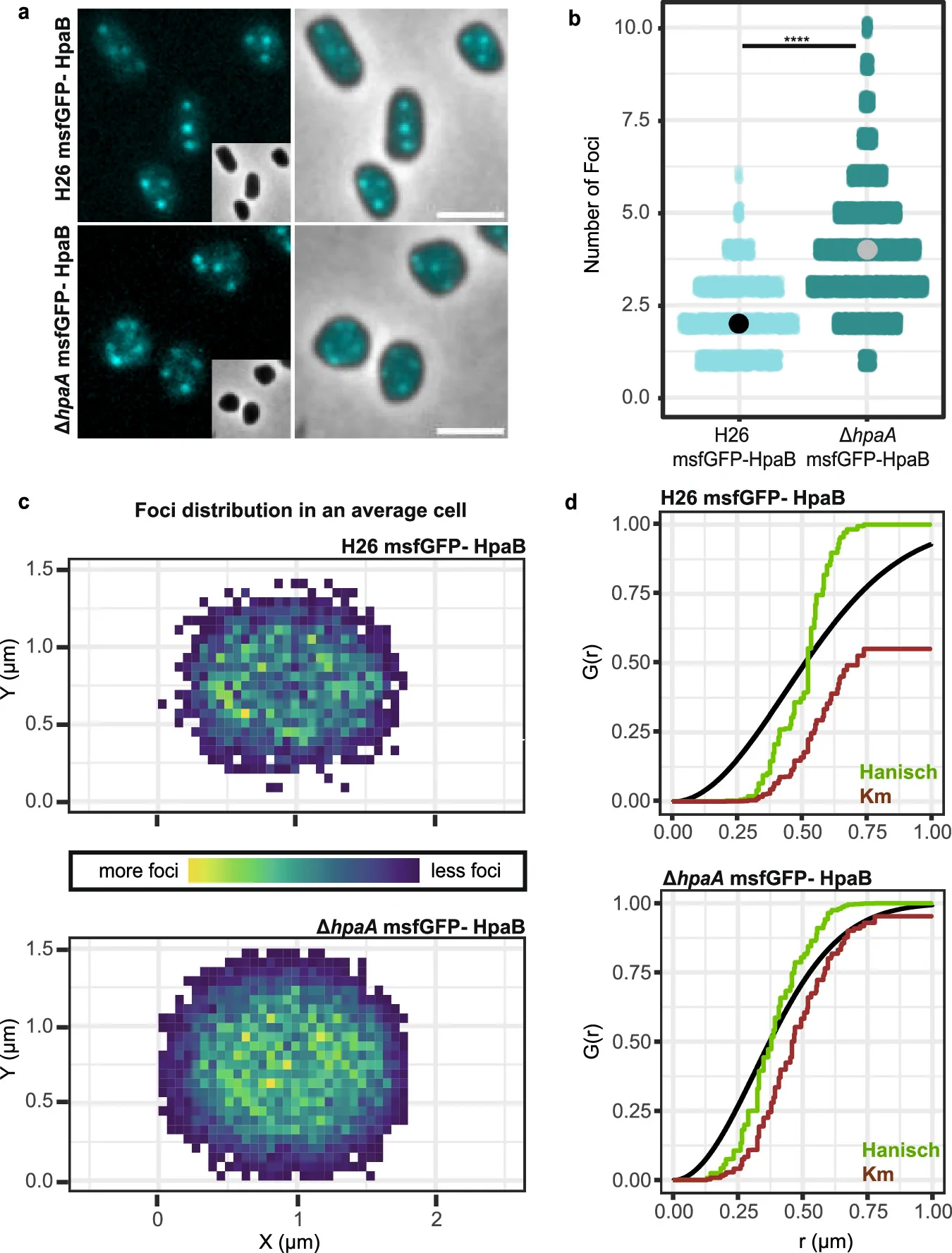
HpaB forms equidistant foci dependent on HpaA for ordered distribution. **a** Widefield fluorescence microscopy showing representative cells OD_600_ of 1.0 with fluorescent foci of HpaB tagged with msfGFP (cyan) in wild type cells and cells lacking *hpaA*. Scale bar = 2 µm. **b** Comparison of number of foci per cell (*y*-axis) in wild type (*n* = 3688 cells) and mutant (*n* = 4740 cells) (*x*-axis) across three replicates; *p* value < 1e−16 Stars indicate significance: **** (<0.0001) calculated using two-sided Kruskal–Wallis test. **c** The distribution heatmap of foci within all the analyzed cells, centered using X/Y coordinates, showing an average likelihood of foci distribution in cells. **d** G-function curve with border-corrected nearest-neighbor cumulative curves using Hanisch (green) and km estimates (brown). The black curve indicates theoretical randomness on the given set of data; the corrected curves show that the HpaB foci are non-randomly distributed in an HpaA-dependent manner. Source data are provided as a Source Data file.

Since we have shown that HpaB binds to pHV3 specifically, it was important to determine the behavior of the protein in vivo. To this end, HpaB-msfGFP, or msfGFP-HpaB were ectopically expressed in *H. volcanii* H26 wild type and Δ*hpaA* cells. Microscopic examination of both variants showed the presence of HpaB condensed in foci (Fig. 6a). We observed an average of 2–3 HpaB foci per cell in presence of natively expressed HpaA. Deletion of *hpaA* led to a significantly increased average number of foci ~ 4–5 foci per cell (Fig. 6b). Moreover, the HpaB foci were closer to each other in absence of HpaA (Supplementary Fig. 6c, d). The high density of the foci in the Δ*hpaA* mutant can be attributed to the fact that the foci number shows a near 2-fold increase, and are likely not able to fuse without HpaA.

To compare the distribution of HpaB foci quantitatively, the microscopic data of wild-type and Δ*hpaA* cells, along with the distribution of fluorescence, were extracted as X and Y data points. These were plotted together to obtain the distribution of foci in an average cell. The nearest neighbor distribution for each focus within a cell shows that HpaB foci are significantly closer to each other in the absence of HpaA (Supplementary Fig. 6c), further indicated by a left shift in the cumulative nearest distance (Supplementary Fig. 6d). Moreover, HpaB foci are differently distributed in the presence and absence of HpaA, wherein a lack of HpaA causes a shift from ordered to a random distribution. The heatmap (Fig. 6c) shows visually where HpaB foci are most likely to be located, and it is clear that HpaA is required for an ordered distribution of the foci such that they neither cluster in the center of the cell nor near the membrane. These data clearly support a model of an active distribution of HpaB foci by HpaA via corralling a few HpaB-DNA complexes together. The increase in the number of foci in the absence of HpaA also indicates that pHV3 copies possibly need corralling via HpaA to be bundled together into HpaB foci.

To quantify the randomness, a G function curve was plotted for both the strains using the cumulative nearest neighbor distance between the HpaB foci within each strain. The G function G(r) measures the distribution of nearest distances of points (foci in our case). Given any set of points on a plane, random distribution of the points would lead to clustering and repelling behavior within the same plane, while ordered distribution would tend closer to equipositioning of the points on the plane (Supplementary Fig. 7). This distribution is then plotted as a G function curve with the cumulative nearest neighbor distances (ecdf) for each point, and corrected for border effects due to a limited plane size using the Hanisch and Kaplan-Meier estimates^94,95^ (Supplementary Fig. 7). In case of random distribution, the corrected curves tend closer to complete spatial randomness (CSR), while an ordered distribution would tend towards compression of the distance on the *x*-axis which can be attributed to equal distances between each point (Supplementary Fig. 7).

**Fig. 7:**
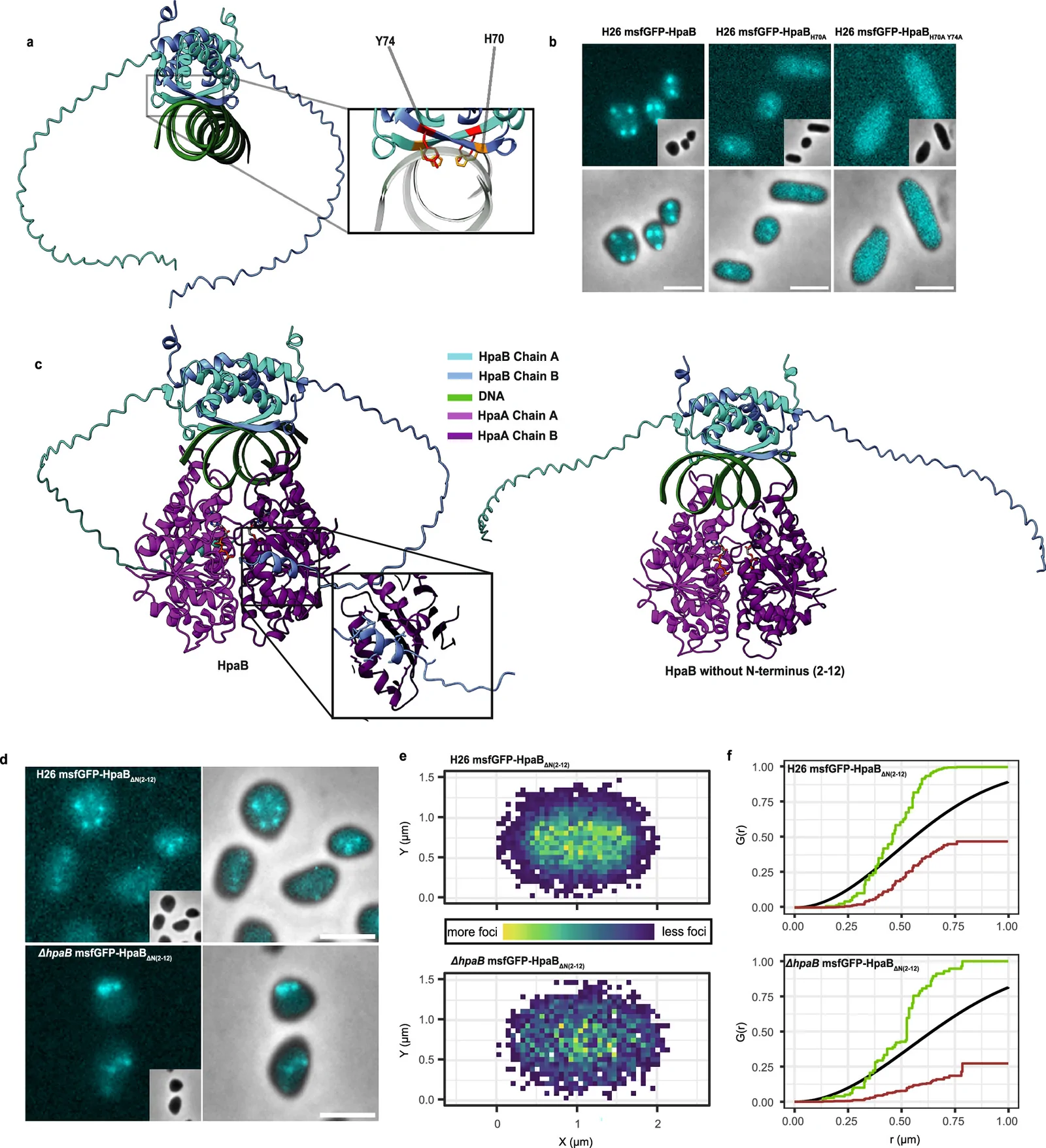
HpaB requires tyrosine at position 74 to form foci, and its N-terminus for ordered distribution. **a** Predicted binding of HpaB dimer with DNA, highlighting important residues in the Ribbon Helix Helix motif. that interact with the DNA strand, tyrosine (Y) in red and histidine (H) in orange. **b** Widefield fluorescence microscopy showing representative cells OD_600_ of 1.0 with fluorescence of HpaB variants tagged with msfGFP in wild type cells showing foci and point mutation variants lacking foci. Scale bar = 2 µm. **c** The predicted binding of HpaB_WT_ and HpaB_ΔN_ with HpaA dimers via the N-terminus of HpaB in focus. **d** Widefield fluorescence microscopy showing representative cells OD_600_ of 1.0 with fluorescent foci of HpaB_ΔN_ tagged with msfGFP (cyan) in wild-type cells and cells lacking *hpaB*. Scale bar = 2 µm. **e** The distribution heatmap of foci within all the analyzed cells (*n*_WT_ = 3959 cells, *n*_Δ*hpaB*_ = 1685 cells) centered using X/Y coordinates, showing an average likelihood of foci distribution in cells. **f** G-function curve with border-corrected nearest-neighbor cumulative curves using Hanisch (green) and km estimates (brown). The black curve indicates theoretical randomness on the given set of data; the corrected curves show that the HpaB foci are non-randomly distributed in an N-terminus-dependent manner. Source data are provided as a Source Data file.

In our case, the ecdf curves were plotted against a theoretical random distribution according to the provided dataset, and are then corrected via Hanisch and Km estimates^94,95^. A random distribution of the foci can be observed in the strain with *hpaA* deletion (Fig. 6d), with the Hanisch and Km estimates tending towards the CSR. On the other hand, in the wild-type cells, it can be seen that the foci start repelling each other while interestingly forming clusters at higher distances and G(r) values (Fig. 7d). The G function curve tends towards more equipositioning via ordered distribution of the foci, with a preference of distance between foci being around 0.4–0.7 µm. These results allow us to conclude that HpaA is required for ordered distribution of the HpaB foci via equipositioning them in two dimensions, and its lack thereof leads to a random distribution of the HpaB foci.

msfGFP-HpaB also showed consistent equipositioning in the absence of native HpaB (Supplementary Fig. 8). Interestingly, while both msfGFP-HpaB, and HpaB-msfGFP were rather consistent with these observations (Supplementary Figs. 9 and 10), only msfGFP-HpaB complemented the growth on xylose (Supplementary Fig. 5e, f).

### HpaB likely binds DNA via its ribbon helix helix motif

We identify HpaB as the cargo-binding adapter for the HpaAB system, which binds pHV3 at the cis-acting *hpaS3* sequence. HpaB, is encoded immediately downstream of HpaA. HpaB showed no sequence homology or significant similarity with any known CBP or analogous proteins. However, structure alignment of predicted structures showed the presence of similar domains with other CBPs like ParG^96^ and SegB^42^, particularly in its RHH motif. ParG (PDB: 1P94)^96^ in particular showed a similar RHH fold with an overall predicted RSMD of 1.0 Å (Supplementary Fig. 11a). On the other hand, when the predicted structure of HpaB was compared with the known structure of SegB (PDB: 7DUV)^42^, an RSMD score of 0.525 Å was predicted with higher similarity in the monomers rather than the dimer (Supplementary Fig. 11b). Structure prediction via Alphafold 3 indicated a presence of the DNA binding region within its RHH motif (Fig. 7a). Two residues, histidine at position 70 and tyrosine at position 74, were predicted to interact with DNA. To test whether these residues had any influence on the in vivo localization of HpaB, variants were generated with residues mutated to alanine to cause a loss of function.

Full-length msfGFP-HpaB showed the presence of clear foci in the cells (Fig. 7b). While HpaB H70A was able to form foci-like structures, they were not comparable to full-length HpaB. Furthermore, HpaB H70A Y74A (double mutation) lacked any discernible foci. The results indicate that the foci formation of HpaB depends on DNA binding via the histidine and tyrosine residues at positions 70 and 74 in the RHH motif. Interestingly, when predicted with the different DNA strands, HpaB always preferred to bind at the *hpaS3* site (Supplementary Fig. 12).

HpaB-msfGFP variants also showed similar in vivo behavior (Supplementary Fig. 13a) qPCR analysis on complementation by C-terminus msfGFP-tagged full-length HpaB, HpaB Y74A, and HpaB H70A Y74Ashows that the two variants show no significant complementation with the pHV3 to main chromosome ratio (Supplementary Fig. 13b).

### HpaB likely interacts with HpaA with its N-terminal domain

The disordered distribution of HpaB foci in the absence of HpaA suggests that the interaction between HpaB and HpaA is essential for in vivo localization of HpaB. Despite the diversity of cargo adapter protein in ParA-like operons, it has been shown that they often interact with their ATPase partner via the N-terminus^78,97–99^.

To determine if HpaB might also have an N-terminus-dependent interaction with HpaA; we modeled the predicted interaction between the two proteins via Alphafold 3^100^. The prediction included an ATP-dependent HpaA sandwich dimer, DNA, and two variants of HpaB: full-length, and a truncated HpaB lacking residues 2–12 (HpaB_ΔN_) (Fig. 7c). Both variants of HpaB were predicted to dimerize and showed DNA interaction via the RHH motif. The N-terminus of each monomer of HpaB formed a small α-helix comprising of 12 residues (Fig. 7ca inset), which was predicted to interact with a monomer of HpaA. Upon repeating this analysis without the 11 residues in the N-terminus of HpaB the α-helix did not form, and the proteins lost their interaction interface (Fig. 7c).

In order to corroborate these models, the in vivo role of the N-terminus was determined by expression of msfGFP-HpaB_ΔN_ and HpaB_ΔN_-msfGFP in H26 and *ΔhpaB H. volcanii*. The H26 cells contained both the native copy of HpaB and the fluorescent HpaB_ΔN_, while the *ΔhpaB* strain expressed only the truncated HpaB. The N-terminus was found not to be essential for the foci formation behavior of HpaB; fluorescent foci were observed in both strains (Fig. 7d and Supplementary Fig. 13c). However, the localization of HpaB_ΔN_ revealed severe differences in both cases. Therefore, the localization and distribution were quantified in both strains as above. In the strain containing both native full length HpaB and ectopically expressed HpaB_ΔN_, where heterodimers of HpaB and HpaB_ΔN_ might form, the foci showed a similar ordered distribution as full length HpaB around the center of the cell (Fig. 7e and Supplementary Fig. 13d), indicative of correct interaction with HpaA, which was shown before to be essential for correct HpaB positioning (Fig. 6). On the other hand, the foci formed by homodimers of HpaB_ΔN_ lost this ordered distribution (Fig. 7f and Supplementary Fig. 13e) and instead had a more clustered or random distribution as demonstrated by the ecdf curves (Fig. 7f and Supplementary Fig. 13e green). The clustering and random distribution of HpaB_ΔN_ is also apparent from the heatmap analysis. Therefore, we suggest from these results that the N-terminus of HpaB is highly likely the interaction domain with HpaA and therefore essential for the ordered distribution of HpaB foci.

## Discussion

Bioinformatic analysis of HpaA shows a high degree of sequence similarity in the conserved ATP Walker motifs with canonical bacterial ParA-ATPases, especially with respect to conserved catalytic and signature lysines, and the invariant glycine residues^26,71^. In canonical ParA systems, dimerization and ATP binding are tightly linked to DNA interaction, enabling dynamic gradients for positioning cellular cargo such as plasmids^69^. The ATP-binding region in the predicted structure of HpaA appears to be sandwiched between two monomers, similar to canonical ParA dimers, meaning that the ATP is stabilized by the signature lysine of the respective other monomer. This brings the P-loops and switch II motifs of both monomers in close proximity once the dimer is formed^71^.

Alphafold3 predictions also suggest that HpaA forms an ATP sandwiched dimer as seen in Soj and MinD from *Bacillus subtilis*^71,101^ for example. Interestingly, the phylogenetic analysis of the full length HpaA sequence places it closer to ParA-like proteins such as MipZ and McdA, rather than Soj or plasmid-borne ParA´s (Fig. 1). The structure of the carboxysome positioning ATPase McdA contains an unusual dimer formation^72^. In ATP bound McdA a lysine residue located far from the P loop facilitates ATP dependent formation of a nucleotide sandwich dimer that positions the two McdA monomers in opposite orientation. This non-canonical lysine is conserved among McdA homologs^72,74^. The sequence of HpaA, however, contains the canonical signature lysine in the Walker motif (Fig. 1a), assuming that the dimerization is not McdA-like. On the other hand, the predicted binding site of HpaA with HpaB seems to be on each monomer rather than a cleft on the dimer interface (Fig. 7c) arguing towards homology to MipZ for adapter binding as MipZ also lacks the hydrophobic cleft seen in proteins like Soj^70,71^.

We show that HpaA is capable of binding DNA non-specifically in the presence of ATP. The HpaA dimer is predicted to form a positively charged DNA-binding surface clustered with lysines and arginines that is embedded within an otherwise negatively charged protein surface, which is likely an adaptation to the hypersaline cytosol of *H. volcanii*^73,102^, which contains close to 2 M KCl (Supplementary Fig. 1). The positively charged patch is consistent with non-specific DNA interaction, which is a hallmark of ParA-like ATPases^71^.

In vivo data further support that HpaA behaves as a bona fide ParA-like protein. Walker motif mutants, including the putative ATP-binding mutant (K16A), ATP-hydrolysis mutant (D40A), and the dimerization mutant (G12V) all show localization patterns consistent with loss of ATP binding or dimerization, leading to either static foci or cytoplasmic dispersion, thereby phenocopying the subcellular localization of known ParA-like proteins such as MipZ and McdA^40,70,74,103^.

Interestingly, the G12V variant of HpaA exhibits homogenous cytoplasmic distribution, recapitulating the dynamics of equivalent McdA mutants^74^. Furthermore, oscillatory behavior of wild-type HpaA was observed in a subset of rod-shaped cells, suggestive of gradient-based positioning mechanisms similar to McdA^38^, MinD^39^, and ParA^104^. The oscillation of HpaA was absent in cells lacking HpaB, suggesting that HpaB modulates HpaA localization, just like the adapter protein is responsible for modulating dynamics of the ParA-like ATPase in other ParAB-like systems^74,77^. ATP-dependent DNA binding, together with the altered in vivo dynamics observed in the absence of HpaB, suggests that HpaA likely possesses ATPase activity that depends on conserved Walker motif residues and is stimulated by interaction with HpaB. However, despite repeated attempts, we were unable to detect ATPase activity in vitro under the tested conditions. We therefore propose that robust ATPase activity may require specific reaction conditions, potentially including appropriate ionic strength, the presence of HpaB, and cognate *hpaS* DNA sequences for full activation.

HpaB was previously annotated as a hypothetical protein due to lack of functional characterization, but our data identify it as a trans-acting element, binding specifically to pHV3 to allow for secondary chromosome partitioning. Sequence analysis shows homology to uncharacterized archaeal proteins but no similarity to bacterial homologs. HpaB is small (~150 amino acids), disordered, and predicted to contain a ribbon-helix-helix (RHH) motif that is commonly found in centromere-binding proteins (CBPs) such as ParG^96^ and SegB^42^. The fact that substitution of the predicted DNA-contacting residues perturbs or abolishes focus formation suggests that HpaB focus formation is a consequence of its direct interaction with pHV3. We interpret the observed foci to represent pHV3 clusters with bound HpaB. Such clustering mirrors known behaviors of ParB-like proteins that undergo liquid-liquid phase separation (LLPS)^24^. The high salt environment in *H. volcanii* may promote or regulate LLPS, enabling HpaB to form localized condensates similar to McdB^105^ or ParB^24^. Thus, we conclude that HpaB is functionally analogous to the CBPs such as ParG from bacteria^96^, or SegB from *S. solfataricus* that also bind DNA via its RHH motif and not via an adapter protein as seen in other archaeal systems such as the *Sulfolobus* NOB8H2 AspA-ParB-ParA system^43^.

It is also interesting to note that the ChIP-Seq shows a higher enrichment of the pHV3 baseline in the presence of HpaB (see above), consistent with the qPCR results which show a decrease in pHV3 copy numbers in the absence of HpaB. ATAC-seq on the *H. volcanii* genome done previously has shown regions in pHV3 to be non-compacted^60^, some of which we now show contain the AT rich *hpaS3* motif. It is also known that ParB-like proteins are involved in preventing DNA-compaction at *parS* sites^18^. Combined with that fact that the distribution of the *hpaS3* motif on pHV3 is adjacent to many genes, we also suggest that HpaB may additionally contribute to local chromosome structuring and transcription landscape.

While the secondary chromosome pHV3 encodes genes involved in xylose metabolism^82,83^, *hpaS3* sites are not found in the vicinity of any gene involved in xylose degradation. We therefore suggest that the growth defects seen with xylose as a carbon source is a direct result of unequal inheritance of pHV3. Inefficient segregation results in daughter cells that fail to inherit the secondary chromosome, leading to slower population growth with cells being selected for more higher pHV3 copy number with sustained growth on xylose, up to the ratio observed in H26 cells. Moreover, in carbon sources where pHV3 is not necessary, the population retained cells with fewer pHV3 copies suggesting that too high pHV3 copies is deleterious for the cells. The functionality of HpaA when provided in trans depends on optimal expression conditions, or the dynamic, concentration-dependent behavior of HpaA, as reported for other pattern-forming or concentration-dependent proteins^106^. Increasing HpaA levels in cells reduces complementation or even causes worse growth than in the absence of HpaA. This is consistent with the reduced robustness in pHV3 copy number inheritance upon overexpression of HpaA. The robustness in pHV3 copy number maintenance between biological replicates suggests that HpaB functions as the primary driver of pHV3 segregation, directly ensuring proper partitioning and maintenance of the secondary chromosome. In contrast, HpaA may play a modulatory role, contributing to its dynamic localization.

HpaA itself is responsible for the ordered distribution of HpaB. In analogy to the McdAB system^38^, HpaA may prevent aggregation of HpaB foci, ensuring their equipositioning. Deletion of *hpaA* results in random distribution of HpaB foci, while deletion of *hpaB* alters the dynamics of HpaA, consistent with mutual interdependence. Moreover, this ordered distribution of HpaB is dependent on the presence of its N-terminus, which is predicted to interact with HpaA. Truncation of the N-terminus leads to a disordered distribution of HpaB foci, quite similar to the loss of ordered distribution in the absence of HpaA. Our data suggest that HpaB depends on an α-helix at the N-terminus to interact with HpaA; quite similar to how the N-terminus in known cargo adapter proteins binds to their ParA-like partners^75,99^. The differences in the foci number and the degree of randomness between the in vivo behavior of truncated HpaB in the absence of wild-type HpaB, and the behavior of HpaB in the absence of HpaA, indicate a role of the N-terminus in more than just HpaA interaction. We speculate that the N-terminus of HpaB may be important for a more regulated condensation of HpaB via interaction with HpaA. Truncation of the N-terminus therefore leads to a more disordered and aggregating behavior, with the aggregates being randomly distributed in the cell.

Equipositioning ensures that each daughter cell inherits plasmid copies, particularly critical in pleomorphic disk-shaped cells where the division plane is not fixed by polarity but rather a consequence of the cell shape^107^. This contrasts with many bacterial ParA-ParB systems, where chromosomes and plasmids are segregated toward specific cellular regions, such as the poles^104,108^. In *H. volcanii*, equipositioning allows for spatial segregation across two dimensions, accommodating the organism’s polyploidy and pleomorphic morphology. The equidistant pattern may be especially beneficial during late-log phase when cells are disk-shaped, and division axes vary.

The dynamic interplay between HpaA and HpaB is conceptually reminiscent of both McdAB and ParAB systems. Like McdA, HpaA may form gradients regulated by local interactions with HpaB foci. The HpaAB system exhibits several features consistent with a Brownian ratchet mechanism. HpaB binds specifically to *hpaS3* sites on pHV3, forms localized foci that are positioned to maximize spacing across the nucleoid, indicating that movement is non-random and spatially regulated. HpaA is dynamic, binds DNA in an ATP-dependent manner, and its localization likely depends on ATP binding and hydrolysis, as shown by mutations that specifically disrupt these activities. Together, these observations suggest that HpaAB uses diffusion coupled to ATP-driven cycles of binding and release to actively partition the secondary chromosome. This behavior is reminiscent of the McdAB carboxysome positioning system, in which McdB-bound carboxysomes are similarly distributed across the nucleoid through interactions with the ATPase McdA^38,74,105^. Both systems appear to employ a comparable strategy of equipositioning via localized binding and dynamic ATPase activity. Deletion of *hpaA* results in a dramatic increase in the number of HpaB foci, from approximately average 2–4 in wild type cells to an average of 4–5, and a maximum of 9–10 in Δ*hpaA* strains. This suggests that in wild-type cells, HpaA is required to corral multiple replicons into shared HpaB foci, whereas in its absence, replicons tend to form independent HpaB foci. These observations suggest that HpaA acts as both a corraler, promoting the clustering of HpaB-bound replicons, and a spatial organizer, biasing their distribution over the nucleoid.

The HpaAB system in *H. volcanii* provides an intriguing example of a hybrid segregation mechanism involving a bacterial origin ParA-like ATPase and an archaeal-specific DNA adapter, similar to the SegAB system from *S. solfataricus*^41,42^. This modular system likely evolved via horizontal gene transfer and adaptation to hypersaline conditions, where protein surface charge and LLPS properties are crucial^60,109^.

The fact that the system enables an equidistant distribution of chromosomes throughout the cytoplasm is likely critical for chromosome inheritance in a polyploid archaeon that is not known to rely on traditional segregation mechanisms. This model of equidistant, repulsion-driven plasmid organization may reflect a broader evolutionary strategy in pleomorphic Archaea to cope with their unique cellular geometry and extreme environments. The fragmented genome of *H. volcanii* requires several segregation systems that ensure stable segregation of the individual replicons. We found that all secondary chromosomes (pHV1-3) contain one HpaAB pair, while the pHV4 replicon contains two of these systems. Since pHV4 is integrated into the main chromosome in the lab strain H26, we think it might be possible that these systems may contribute to the segregation of the main chromosome in *H. volcanii*.

In summary, the HpaAB system shows mechanistic and evolutionary divergence from canonical plasmid partitioning systems, combining elements of both spatial regulation (via HpaA gradients) and phase-separated anchoring (via HpaB foci). It represents the first active chromosome segregation system identified in a highly polyploid organism, underscoring the versatility of segregation mechanisms across life domains. Further biochemical and phylogenetic analyses are needed to dissect the ATP cycle of HpaA and its interaction with HpaB, and to trace the evolutionary origin of this modular system.

## Methods

### Growth conditions and media

All strains are based on the *H. volcanii* H26 strain serving as the wild type. Strains, plasmids and oligonucleotides used in this study are listed in Supplementary Tables S3–5. All strains were grown at 42 °C under aerobic conditions in synthetic medium^110^ supplemented with either casamino acids (1% w/v), D-glucose (20 mM) or D-xylose (20 mM). The wild-type and deletion strains that did not carry a plasmid were grown in the presence of uracil (50 μg/ml). Strains transformed with either the empty vector pTA963^111,112^ or derived plasmids were grown in medium without the addition of uracil to select for plasmid-carrying cells. Cells were always pre-inoculated directly from glycerol stocks into media containing 1% casamino acids for 24–48 h and then transferred to media containing the desired carbon source.

Growth of the wild type and of the Δ*hpaA* and Δ*hpaB* deletion strains was compared on D-xylose. For complementation of the uracil auxotrophy, these strains carried the empty vector pTA963. For in trans complementation, the deletion mutants were transformed with plasmids derived from pTA963, encoding the corresponding genes (*hpaA* or *hpaB*) under the control of the tryptophan-inducible promoter p.tnaA. Precultures of the strains were grown on 1% casamino acids supplemented with 0.1 mM tryptophan. Growth experiments were performed in 100 ml flasks containing 20 ml synthetic medium supplemented with 20 mM xylose and 0.03 - 0.1 mM tryptophan by measuring the optical density at 600 nm (OD600). Growth was started at an OD600 of 0.05 and followed for approximately 80 h.

### Generation of deletion mutants

Gene deletion mutants of *hpaA* and *hpaB* were constructed using the pop-in/pop-out strategy^58^. The 500 base pairs upstream and downstream of the genes, excluding the genes, were fused and cloned into the suicide vector pTA131 using Gibson’s assembly^113^. *H. volcanii* H26 cells were transformed with the plasmids after multiplication in *E. coli* (XL1 Blue MRF). *H. volcanii* H26 was transformed using polyethylene glycol 600^114^. Pop-in cells, which contain the plasmid integrated into the host genome via homologous recombination, were selected via growth of colonies on agar plates without uracil, containing 1% casamino acids. Correct clones were verified for presence of plasmid via colony PCR, and passaged 5 times in synthetic medium to allow allelic integration into all copies of the genome. Pop-out clones were subsequently generated by plating on agar plates containing both uracil and 5-fluoroorotic acid (5-FOA) (50 µg/ml). 5-FOA acted as the selective agent, as it is toxic to cells containing the *pyrE2* gene. Cells that had undergone a second homologous recombination event (pop-out) were able to grow, leading to either wild-type or deletion variants, which were screened and selected via colony PCR.

### Generation of strains expressing fusion proteins

Plasmids were constructed via restriction enzyme-based insertion of genes of interest, or via Gibson cloning^113^. Polymerase chain reactions were carried out using Q5 or Phusion® HF polymerase (New England Biolabs) according to the manufacturer’s instructions. Purified genomic or plasmid DNA served as the template. Primers with overlaps were designed via NEBuilder (NEB) for fusion proteins and Gibson assembly constructs. Point mutations and indels were introduced using mutated primer overlaps via Gibson assembly or via Q5 site-directed mutagenesis (NEB). Primer design for the latter was done via the NEBaseChanger tool (NEB). Plasmid pTA963^111^ was used as a basis to construct controlled expression of fluorescently tagged HpaA or HpaB variants. For plasmids that were constructed via Gibson assembly, the proteins of interest were amplified from the genome and fused with a fluorophore of interest via a GGGGSGGGGS linker. The fusion protein was controlled via the tryptophan-inducible promoter p.tnaA. Induction was performed with 1 mM tryptophan for 1 h when necessary.

### Purification of HpaA and electrophoretic mobility shift assay (EMSA)

Plasmids for 6xHis-HpaA were constructed as described above and used for transformation of *H. volcanii* H1209. The transformed H1209 cells were grown in synthetic medium supplemented with 0.1 % casamino acids, 0.1 % bacto peptone, and 0.5% yeast extract  at 37 °C shaking at 160 rpm till an OD_600_ of around 0.3 was reached. Expression was induced by addition of 2 mM tryptophan. The cells were allowed to grow up to an OD_600_ of around 2.0 before harvesting via centrifugation. For purification, cell pellets were resuspended in Buffer A (100 mM Tris, 1.5 M KCl, 10% glycerol, 5 mM imidazole, and protease inhibitors (Roche cOmplete^TM^). The pH was adjusted to 7.8). Cells were disrupted using a French press, and debris was removed by centrifugation at 41,657 rcf for 30 min. The supernatant was passed through a Ni-NTA column, and elution was performed with 200 mM imidazole. The eluted fractions were then loaded on a Superdex 200 16/60 column (GE Healthcare), and eluted by an isocratic flow in Buffer C (50 mM MOPS, 1.5 M KCl, 10% glycerol, 2 mM MgCl_2_, pH adjusted to 7.5).

The eluted protein was exchanged into a binding buffer (pH 7.5, 50 mM HEPES, 2 M NaCl, 10% sorbitol, 15 mM MgCl_2_). Multiple concentrations of protein were prepared by dilution in binding buffer, and they were incubated (40 ul reactions) with 100 ng of a 250 bp dsDNA probe generated by PCR, and with 2 mM nucleotide (ADP, ATP) at 22 °C for 25 min. The protein-DNA-nucleotide solutions were loaded (without loading dye) onto an 8% polyacrylamide gel in TB Buffer and run at 4 °C at 60 V for 1 h, followed by 120 V for 2.5 h in 0.5x TB Buffer (45 mM Tris, 45 mM boric acid, pH 8.3). After electrophoresis, the gels were incubated in an ethidium bromide bath for 15 min, followed by de-staining for 15 min, and imaged using a UV lamp.

### Fluorescence microscopy

For still images, fluorescence microscopy was performed with exponentially growing cells mounted on agarose-coated slides (1% agarose in growth medium). Cultures were grown in synthetic media with glucose as a carbon source to an OD_600_ of 1.0 to obtain plate morphologies, and spotted on agarose pads and allowed to dry at room temperature, followed by covering with a glass coverslip. Images were acquired on an Axio-Observer 7 fluorescence microscope (Carl Zeiss) equipped with a Colibri 7 LED light source (Zeiss) with an EC Plan Neofluar 100 x/1.3 oil Ph3 objective, a 2.5 x optovar, and a Hamamatsu ORCA-R2 camera. msfGFP was visualized with a filter set 38 HE (BP470/40 FT 495 BP 525/50) and excited at 488 nm (100% Colibri LED intensity) for 400 ms, mScarlet-I was visualized with a filter set 43 HE (BP550/25 FT570 BP605/70) and excited for 500 ms, while mTurqoise2 was visualized with a filter set 47 HE (BP 436/25 FT 455 HE BP 480/40) and excited for 100 ms. The microscope was equipped with an environmental chamber set to 42 °C. Digital images were acquired with Zen Blue (Zeiss) and analyzed and edited using FIJI (version 2.17)^115^.

Time-lapse experiments were performed on exponentially growing cells after dilution to an OD_600_ of 0.02 in synthetic media and loaded on a 1% agarose pad. Images were taken in 1 min intervals. Imaging of live cells was performed with the same settings used for imaging cells mounted on agarose.

Computational analysis of agarose-mounted cells was performed using the MicrobeJ plugin^116^ from the image analysis platform FIJI (Version 2.17). MicrobeJ algorithm was used for automatic detection of cell (using restricted cell length and area to recognize real cells) and foci for extraction of corresponding regions of interest from all image channels. The regions of interest (cell contours and foci) were converted into a map of X and Y coordinates. Data were further evaluated via these X and Y coordinates using the software R^117^ and RStudio (Version 4.5)^118^.

### Super resolution microscopy and single particle tracking

Exponentially growing *H. volcanii* WT and Δ*hpaB* cells expressing HpaA-HaloTag ectopically were stained with 50 nM HaloTag TMR Ligand (Promega) for 30 min at 42 °C at 600 rpm on a standard 2 ml heat block (Eppendorf). Cells were harvested (4.5 g, 2 min, RT) and washed three times in filtered synthetic media.

Slides were cleaned with 1 M KOH overnight, rinsed with deionized H_2_O, and dried with pressurized air. Agar pads were prepared with the aid of gene frames (Thermo Fisher) and 1% (w/v) low-melting agarose (agarose, low gelling temperature, Sigma-Aldrich) in sterile filtered synthetic media (0.2 μm pores).

Single molecule localization microscopy (SMLM) was performed with an Elyra 7 (Zeiss) inverted microscope equipped with pco.edge sCMOS 4.2 CL HS cameras (PCO AG), connected through a DuoLink (Zeiss), only one of which was used in this study. Cells were observed through an alpha Plan-Apochromat 63×/1.46 Oil Korr M27 Var2 objective in combination with an Optovar 1 × (Zeiss) magnification changer, yielding a pixel size of 97 nm. During image acquisition, the focus was maintained with the help of a Definite Focus 2 system (Zeiss). Fluorescence was excited with a 561 nm (100 mW) laser, and signals were observed through a multiple beam splitter (405/488/561/641 nm) and laser block filters (405/488/561/641 nm) followed by a Duolink SR DUO (Zeiss) filter module (secondary beam splitter: LP 560, emission filters: LP570). For each time lapse series, 10,000 frames were taken with 20 ms exposure time (∼24 ms with transfer time included) and 50% 561 nm intensity laser in TIRF mode (62° angle).

For single-particle tracking, spots were identified with the LoG Detector of TrackMate v6.0.1^119^, implemented in Fiji 1.53, an estimated diameter of 0.5 μm, and median filter and sub-pixel localization activated. The signal-to-noise threshold for the identification of the spots was set at 7. To limit the detection of ambiguous signals, frames belonging to the TMR bleaching phase (first 1000 frames) were removed from the time lapses prior to the identification of spots. Spots were merged into tracks via the Simple LAP Tracker of TrackMate, with a maximum linking distance of 500 nm, two frame gaps allowed, and a gap-closing maximum distance of 0 nm. Only tracks with a minimum length of 5 frames were used for further analysis.

To identify differences in protein mobility and/or behavior, the resulting tracks were subjected to mean-squared-displacement (MSD), square displacement (SQD) analysis, as described previously^120^. Both analytical approaches relied on SMTracker 2.0^121^.

The average MSD was calculated for four separate time points per strain (exposure of 20 ms—*τ* = 24, 48, 72 and 96 ms), followed by fitting of the data to a linear equation. The last time point of each track was excluded to avoid track-ending-related artifacts. The cumulative probability distribution of the square displacements (SQD) was used to estimate the diffusion constants and relative fractions of up to three diffusive states (‘fast’, ‘slow’, and ‘static’)^122^.

### qPCR

The genomic DNA was extracted from samples collected during growth on xylose and cas-amino acids using the Wizard® Genomic DNA purification kit (Promega) according to the manufacturer’s manual with the following changes. After centrifugation with the protein precipitation buffer to separate the cell debris, the DNA was pipetted twice with a 1 ml pipette tip to get a homogeneous solution; then the supernatant was incubated in isopropanol overnight at −20 °C. The concentration and purity of the isolated genomic DNA were verified using a nanophotometer, wherein the A260/A280 ratio was around 1.8, and it was used for qPCR or stored at −20 °C.

DNA amplification was performed using 2x qPCR Mastermix SYBR blue (GeneON, Y220) according to the manufacturer’s manual. Reactions for plasmid and chromosome contained 200 nM of the respective oligonucleotides and 5 ng genomic DNA (total reaction volume of 25 µl). Samples were measured in technical triplicates via an AriaMx, and *C*_t_ values were determined using the Agilent Aria Software 1.8 v. Oligonucleotide efficiencies were estimated by calibration dilution curves and slope calculation; data were analyzed by 2^−Δ^*C*^T^ method accounting for dilution factors and sample volumes used for DNA purification.

### Chromatin immunoprecipitation (ChIP) sequencing

In vivo ChIP-seq on *H. volcanii* HpaB was performed with strains lacking the native *hpaB* locus and instead harboring a plasmid expressing a 6xHis-tagged HpaB in trans, or just the His-tag sequence. Exponentially growing cells at OD_600_ 0.8–1.0, grown in synthetic media with casamino acids, were crosslinked at 24 °C with 1% formaldehyde shaking for 25 min and quenched with 0.125 M glycine (10x dilution from a stock of 1.25 M glycine in growth media). The cells were pelleted at 4500 × *g* for 15 min, and washed twice with ice cold 0.125 M glycine. After pelleting, the fixed cells were resuspended in cold lysis buffer (50 mM HEPES-KOH, 140 mM NaCl, 1 mM EDTA, 1% Triton X-100, 0.1% deoxycholate, 0.1 mg/ml RNaseA, EDTA-free protease inhibitor cocktail) (pH 7.5). Further cell disruption and shearing of genomic DNA to less than 700 bp length was done by sonication using an ultrasonic cell disruptor in a cold block (Branson Ultrasonics Sonifier™; 20% amplitude, pulse 0.5 s on/off, 12 × 20 s with 30 s intervals). Anti-His magnetic beads (Sigma-Aldrich) were equilibrated in lysis buffer twice before introducing them to the cell lysate. The beads were incubated with cell extract overnight at 4 °C. Thereafter, beads were washed twice in cold lysis buffer, twice in buffer W1 (Lysis buffer with 500 mM NaCl), twice in buffer W2 (10 mM Tris-HCl, 250 mM LiCl, 0.5% Triton X-100, 0.5% deoxycholate, 1 mM EDTA) (pH 8.0), and once in TE buffer (10 mM Tris-HCl pH 8, 1 mM EDTA). The beads were finally resuspended in TE/SDS buffer (10 mM Tris-HCl pH 8, 1 mM EDTA, and 1% SDS). Protein-DNA complexes were eluted at 65 °C for 20 min in two rounds; beads were removed, and the crosslinks were reversed with the eluate at 65 °C overnight. Phenol–chloroform extraction was performed and yielded DNA pellets.

DNA pellets were resuspended in nuclease-free water and sent to Genewiz from Azenta Life Sciences for sequencing. Briefly, DNA was end-repaired and adenylated. Adapters were ligated after adenylation of the 3’ ends, followed by enrichment by limited-cycle PCR. The DNA libraries were validated using the Agilent 5600 Fragment Analyzer and quantified using the Qubit 4.0 Fluorometer. The libraries were multiplexed on a flow cell and loaded on the Illumina NovaSeq 6000 instrument according to the manufacturer’s instructions. The samples were sequenced using a 2 × 150 paired-end (PE) configuration. Image analysis and base calling were conducted by the NovaSeq Control Software on the NovaSeq instrument. Raw sequencing data (.bcl files) generated from Illumina NovaSeq were converted into fastq files and de-multiplexed using Illumina’s bcl2fastq software.

Further analysis on the obtained sequences was performed on the Galaxy web platform^123,124^. The raw sequences were cleaned, sorted, deduplicated, and reads were aligned to the *H. volcanii* DS2 genome sequence (GenBank ID GCA_000025685.1). The coverage of the reads on the genome was normalized as counts per million bases read and visualized using the Integrative Genomics Viewer (IGV)^125^.

### Structure prediction

Alphafold 3^100^ was used to predict all structures of HpaA and HpaB variants in Figs. 1, 7 and Supplementary Figs. The same seed was specified for all mutants of HpaB. Predicted structures with pLDDT scores and Predicted Alignment Errors are shown in Supplementary Fig. 14.

### Statistical analysis

All statistical analyses for cell morphologies, qPCR pHV3 to main chromosome ratios, nearest neighbor distance distributions, and linear regressions were performed in R (version 4.5)^117^ and Studio (version 2025.09.2 + 418)^118^. Morphological parameters (cell length, width), nearest neighbor distances of foci as x and y coordinates on a Cartesian plane, and cell profiles were extracted for each individual cell from FIJI using the plugin MicrobeJ^116^. To test for differences among strains (wild type, Δ*hpaA*, and Δ*hpaB*), the Kruskal–Wallis test was used for each parameter, as the data were not normally distributed (Shapiro–Wilk test, *p* < 0.05). When the Kruskal–Wallis test indicated a significant difference (*p* < 0.05), Dunn’s post-hoc tests with Benjamini–Hochberg false discovery rate (FDR) correction were performed to compare each mutant strain against the wild type (WT). Adjusted p-values were used to determine statistical significance and summarized as follows: *p* < 0.05 (*), *p* < 0.01 (**), and *p* < 0.001 (***). Effect sizes were determined via Vargha and Delaney’s A. All tests were two-tailed unless otherwise noted.

### Reporting summary

Further information on research design is available in the Nature Portfolio Reporting Summary linked to this article.

## Supplementary information

Supplementary Information

Reporting Summary

Transparent Peer Review file

## Source data

Source Data 1

Source Data 2

Source Data 3

Source Data 5

Source Data 6

Source Data 4

## Data Availability

All data supporting the findings of this study are available within the article and its Supplementary Information. Source data underlying the graphs and quantitative analyses, and the accession numbers and links to sequences and structures used are provided with this paper in the Source Data file. The ChIP-seq data generated in this study have been deposited in ENA under accession number PRJEB125767 and are publicly available. Any additional data generated during the current study are available from the corresponding author upon request. Source data are provided with this paper.
