# Supplementary material for: Equipositioning of chromosomes in the polyploid archaeon Haloferax volcanii by HpaAB
Source: Nat Commun. 2026 Oct 30;17:11296. doi: 10.1038/s41467-026-78993-w (PMC13722703; doi:10.1038/s41467-026-78993-w)
Supplement: Supplementary file 1 [file 41467_2026_78993_MOESM1_ESM.pdf]

Supplementary Information

Equipositioning of Chromosomes in the Polyploid Archaeon *Haloferax volcanii* by HpaAB

Kartikeyan Premrajka, Ulrike Johnsen, Holger Preidel, Jascha Wienberg, Chiara Corleis, Marius Ortjohann, Giacomo Giacomelli, Marc Bramkamp*

Institute for General Microbiology, Kiel University, Germany;

**Correspondence:* Marc Bramkamp, Institute for General Microbiology, Kiel University, Germany. E−mail: bramkamp@ifam.uni−kiel.de

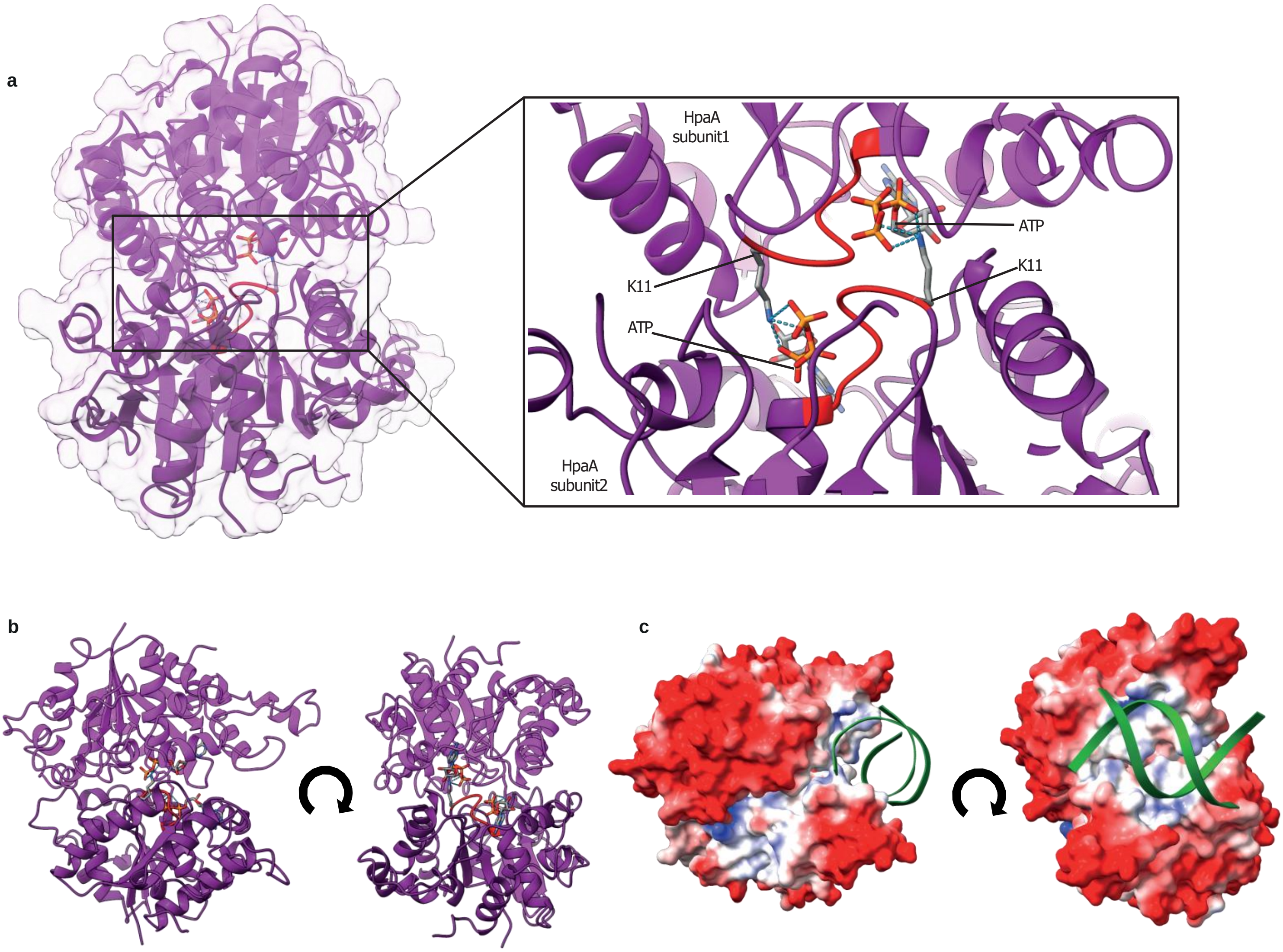

Supplementary figure 1: HpaA predicted dimer shows an ATPase motif sandwich.

(a) Predicted HpaA dimer structure using AlphaFold showing the conserved Walker A motif in red and ATP inside a sandwich, with zoomed in view at the interaction of the signature lysine K11 from each subunit with ATP of the other subunit. (b and c) Predicted structure (rotated 90°) of the dimer showing majorly negatively charged surface (red), and a positive region where the DNA (green) might bind (blue)

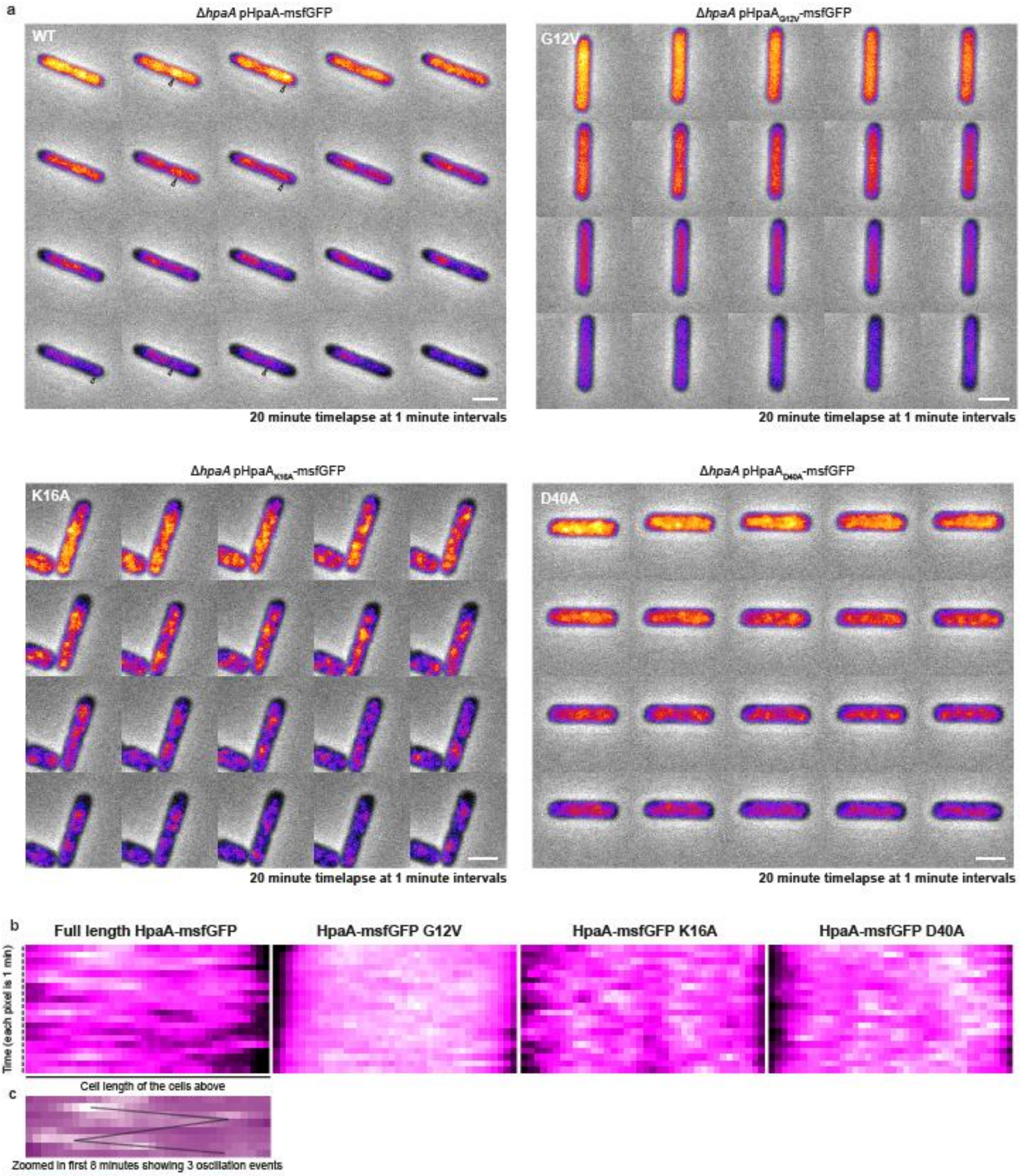

Supplementary figure 2: HpaA-msfGFP variants show different *in vivo* oscillatory behaviour.
Representative time lapses of cells of Δ*hpaA* with over-expressed HpaA-msfGFP variants showing different protein behavior *in vivo* in "fire" colored look-up where yellow indicates higher intensity, and purple indicates lower intensity. Cells were grown to an OD of 0.02, spotted on a 1% agar pad, and images were taken for 20 minutes at 1-minute intervals. The non-substituted HpaA-msfGFP shows a small degree of oscillation of a high intensity zone within 2 minutes on panels 2/3 and 7/8. On the other hand, the variant G12V is completely homogenous. While D40A, and K16A show a high degree of dynamic behavior, we did not observe any oscillation like movements. Scale bar indicates 2 µm. (b) Kymographs of the cells imaged above showing intensity peaks using the magenta hot scale (low – medium – high: black – magenta- white). Wild type HpaA shows alternating peaks at poles, while the others do not. Y axis indicates time, X axis indicates cell length. (c) zoomed in first 8 minutes of the full length HpaA-msfGFP highlighting 3 oscillation events (black bars) which are followed by persistence.

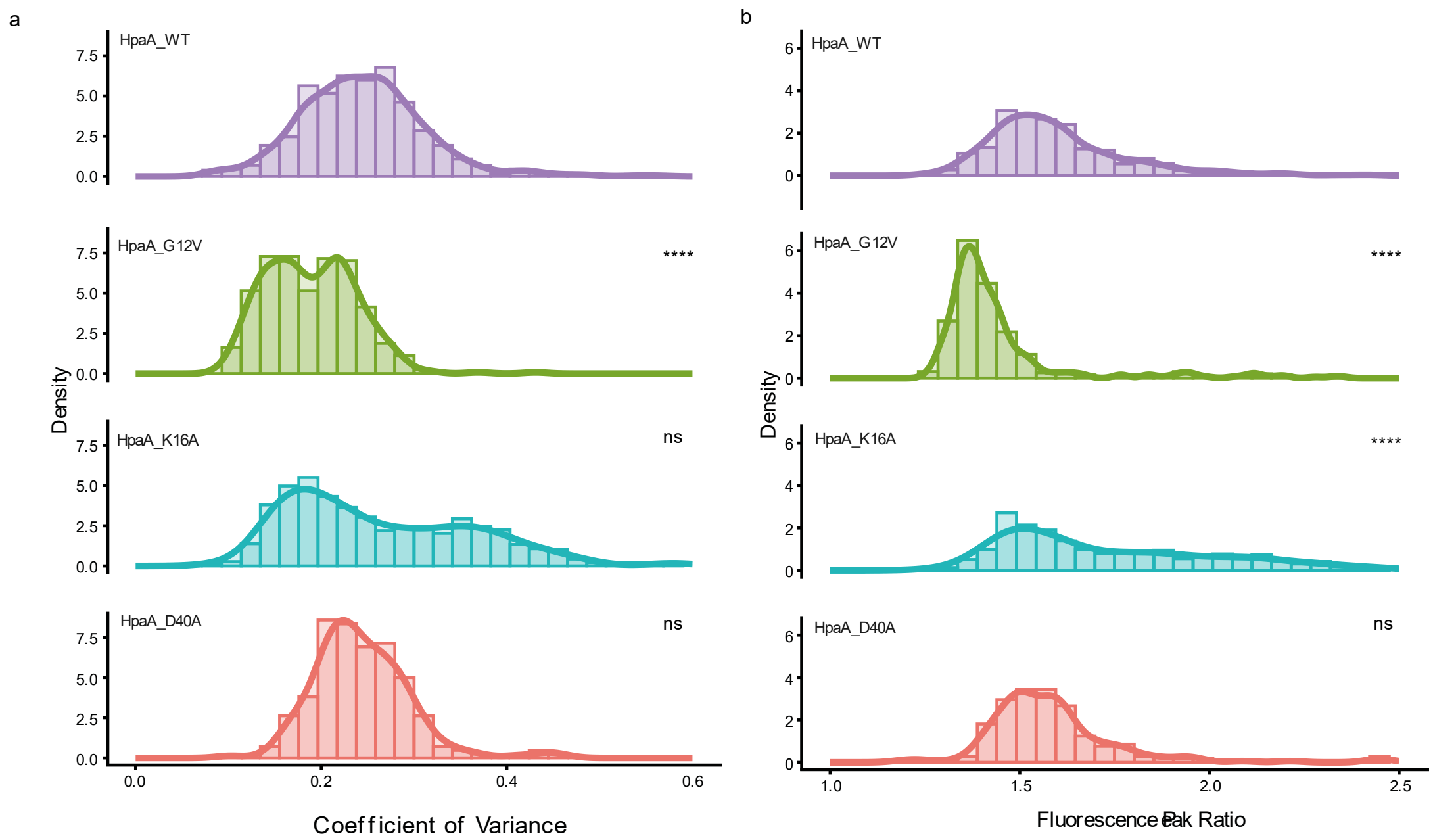

Supplementary figure 3: Coefficient of variance, and fluorescence peak ratio are different in HpaA-msfGFP variants.

Histograms with overlayed density plots showing the distribution of (a) CV, and (b) FPR in a population of HpaA-msfGFP variants indicating a shift in the heterogeneity of fluorescence signal in foci, cloudy clusters, or diffused populations. Lower CV indicates more homogenous signal, lower FPR indicates absence of foci. Stars indicate significance values. $p < 0.05$ (*), $p < 0.01$ (**), and $p < 0.001$ (***).

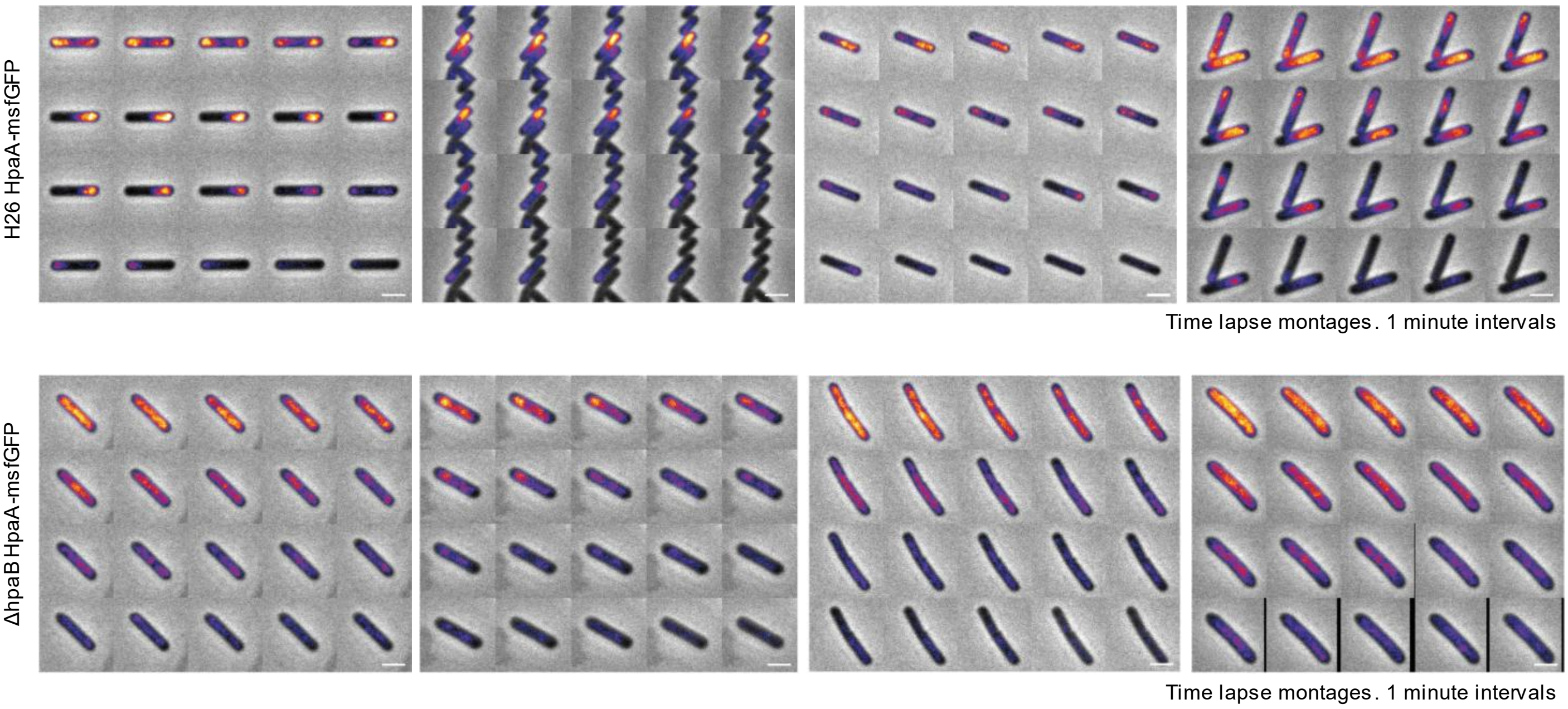

Supplementary figure 4: HpaA-msfGFP lacks oscillatory behaviour in absence of HpaB.
Representative time lapses of cells of H26 and Δ*hpaB* cells with over-expressed HpaA-msfGFP showing different protein behaviour in vivo in "fire" coloured look-up where yellow indicates higher intensity, and purple indicates lower intensity. Cells were grown to an OD of 0.02, spotted on a 1% agar pad, and images were taken for 20 minutes at 1-minute intervals. HpaA-msfGFP shows clear oscillation of high intensity zones from pole to pole within 1-2 minutes followed by persistence on the new pole for 5-6 minutes. On the other hand, in the absence of HpaB we did not observe any oscillation like movements of HpaA-msfGFP. Scale bar indicates 2 µm.

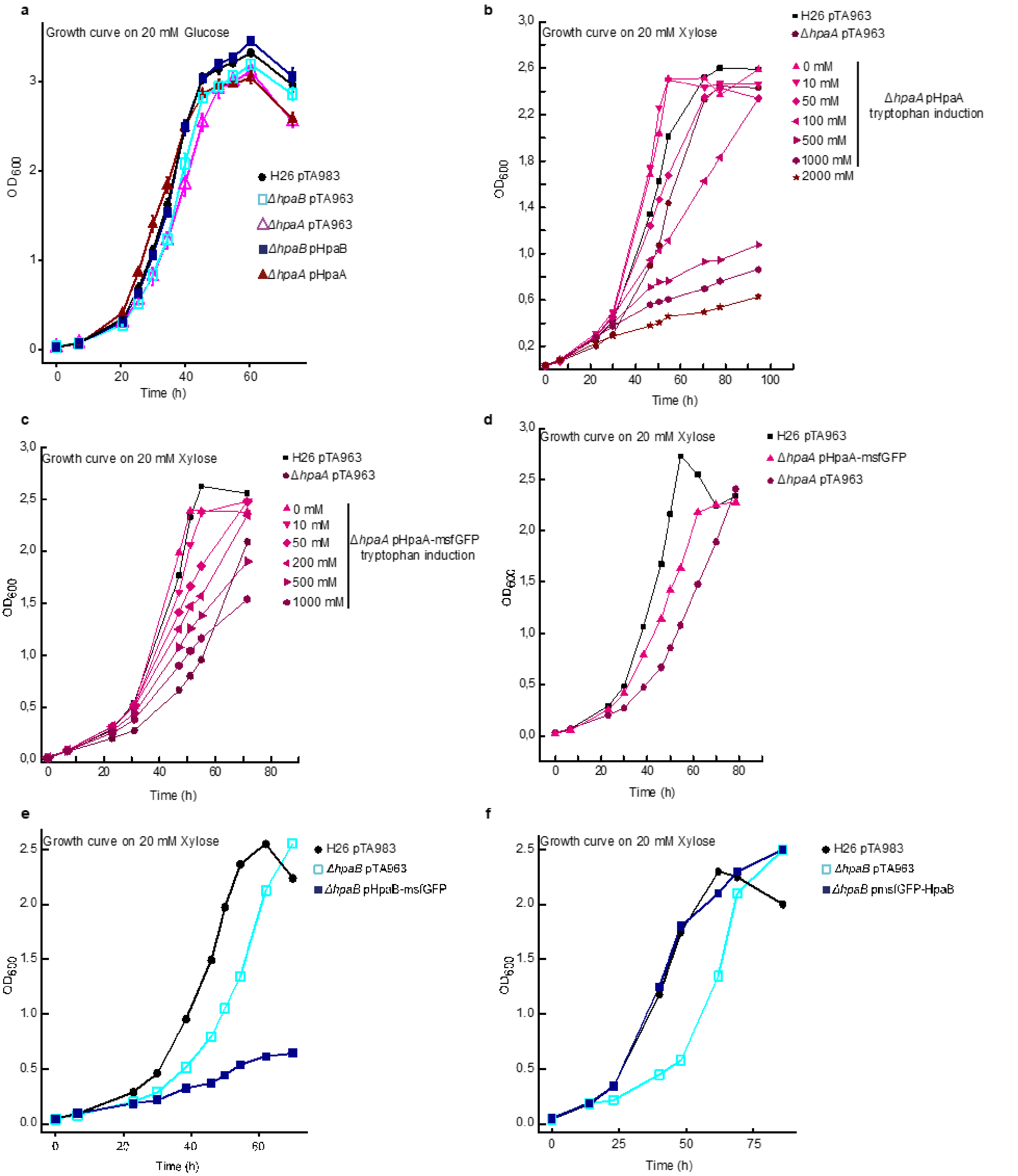

Supplementary figure 5: Growth behaviour of *H. volcanii* in presence and absence of HpaA and HpaB in xylose and glucose.

Cells were grown in optimal conditions at 42° C, 160 RPM, and 2.1 M NaCl containing synthetic media supplemented with 20 mM glucose (a), or xylose (b - e). (a) All strains remain viable with no severe growth defects when grown on glucose in the absence of HpaA or HpaB. Titration of induction of HpaA (b) and HpaA-msfGFP (c) with different tryptophan concentrations shows better complementation with lower induction. (d) HpaA-msfGFP complementation with 30 µM Tryptophan induction which is used for fluorescence microscopy. Complementation with HpaB-msfGFP (e) and msfGFP-HpaB (f) with 100 µM Tryptophan induction.

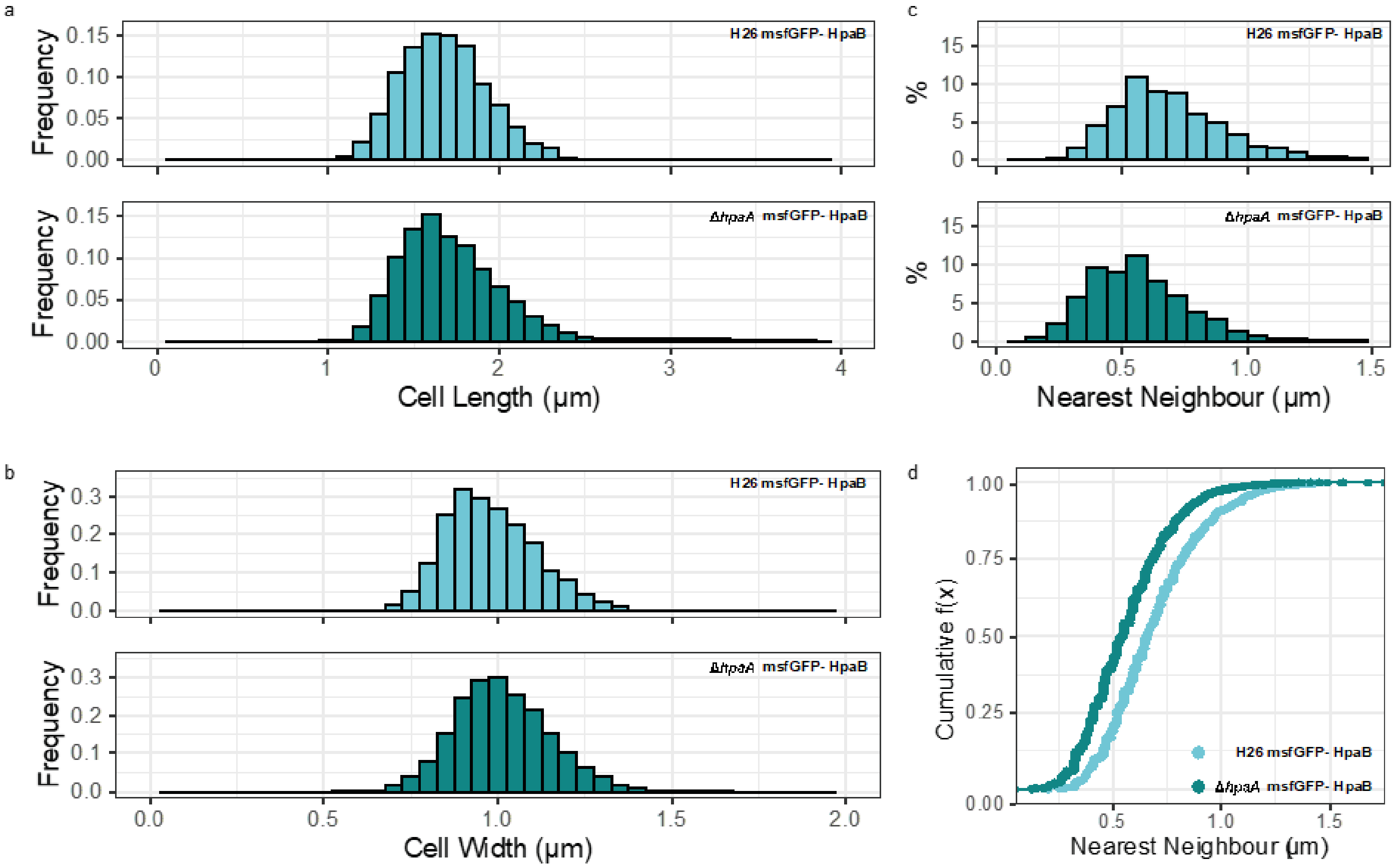

Supplementary figure 6: Cell parameters showing a difference in foci behaviour of msfGFP-HpaB in absence of HpaA.

Differences in cell length (a), and cell width (b) between the two strains represented as histograms. (c) Nearest neighbour distances for each focus in all cells represented as a histogram showing a left shift in absence of HpaA indicating increased closeness between foci for any given msfGFP-HpaB focus. (d) Cumulative distance function of the foci showing a left shift in absence of HpaA. ECDF if used further for G function.

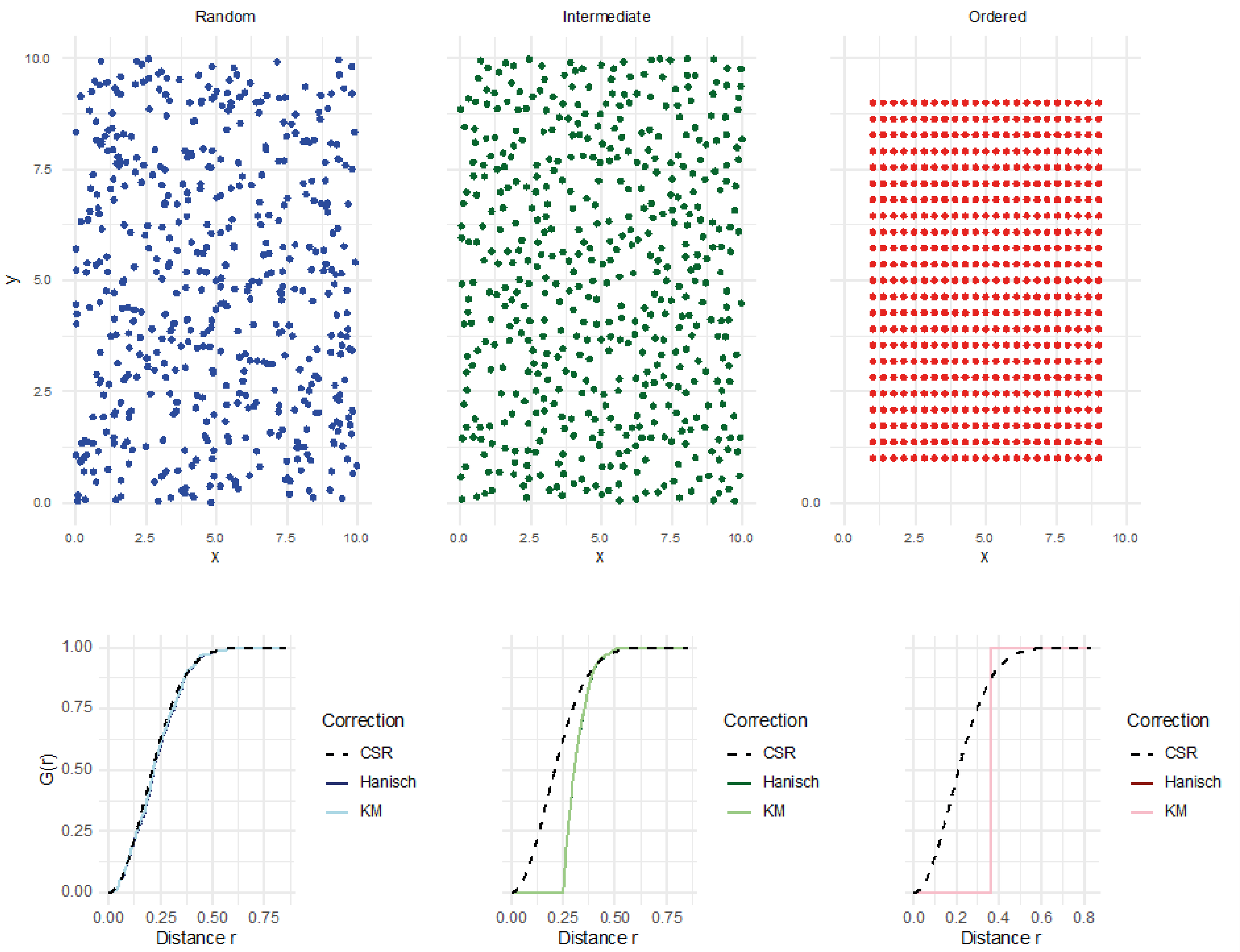

Supplementary figure 7: Simulated representation of point distribution and G function curves

The points on a plane in a random, intermediate, or ordered distribution showing clusters and repelling behaviours in the same plane for random (blue), decreased randomness in intermediate (green), and equipositioning in ordered (red). The G-function curve with border corrected nearest neighbour cumulative curves using Hanisch3 (bold) and km4 estimates (not-bold). The non-continuous black curve indicates complete spatial randomness on the given set of data; the corrected curves show how the G-function would look for random distribution (overlapping estimates with CSR), intermediate distribution (compression of estimates to a repelling behaviour), and ordered distribution (compressed estimates leading to a small range of distance on x- axis).

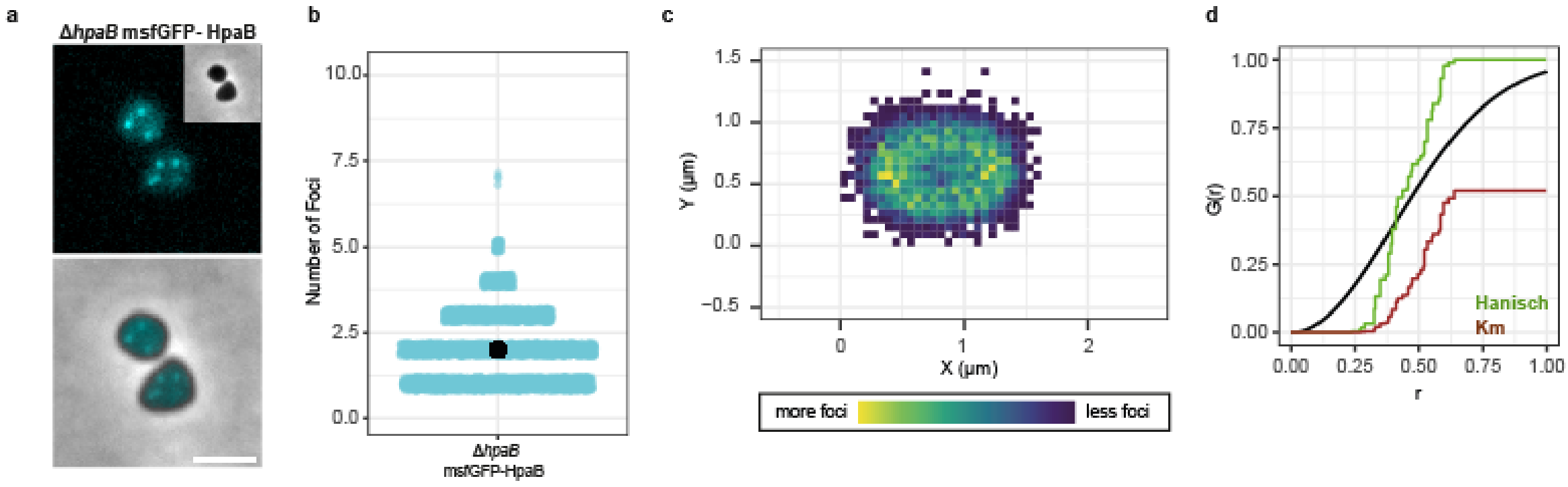

Supplementary figure 8: msfGFP-HpaB forms equidistant ordered foci in *H. volcanii* Δ*hpaB*

(a) Representative images of H. volcanii Δ*hpaB* cells showing msfGFP-foci. Cells were grown in optimal conditions to an OD of 0.5 to obtain plates, spotted on 1% agar pad, and imaged with the same parameters as H26 and Δ*hpaA* cells. (b) Foci number distribution showing a mean of 2 foci per cell. (c) Heatmap of an average cell using aggregate of all analysed cells showing likelihood of finding a focus higher in a ring shape around the centre of the cell. (d) G function curve showing a non-random more ordered distribution of msfGFP-HpaB foci. n = 3468 cells. Scale bar indicates 2 µm.

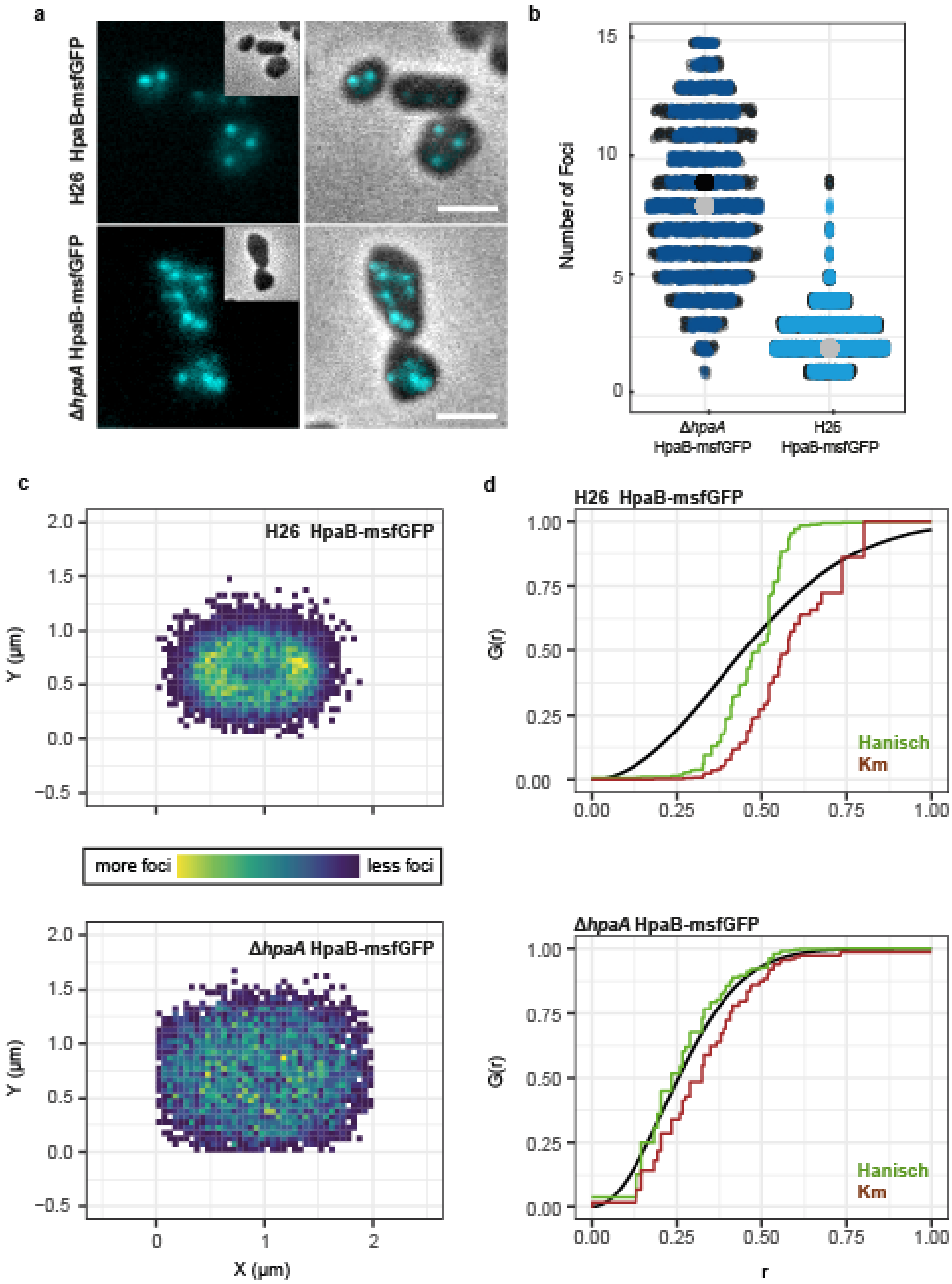

Supplementary Figure 9: HpaB foci numbers, and equipositioning are dependent on HpaA.

(a) Widefield fluorescence microscopy showing representative cells with fluorescent foci of HpaB tagged with msfGFP (cyan) in wild type cells and cells lacking HpaA. Scale bar = 2 µm. (b) Comparison of number of foci per cell (y-axis) in wild type and mutant (x-axis); p value < 1e-16 calculated using two sided Kruskal-Wallis test, n > 7000 cells (>3500 cells per replicate). Colours for each strain indicate two biological replicates. (c) The distribution heatmap of foci within all the analysed cells centred using X/Y coordinates showing an average likelihood of foci distribution in cells. (d) G-function curve with border corrected nearest neighbour cumulative curves using Hanisch (green) and km estimates (brown). The black curve indicates theoretical randomness on the given set of data; the corrected curves show that the HpaB foci are non-randomly distributed in an HpaA dependent manner.

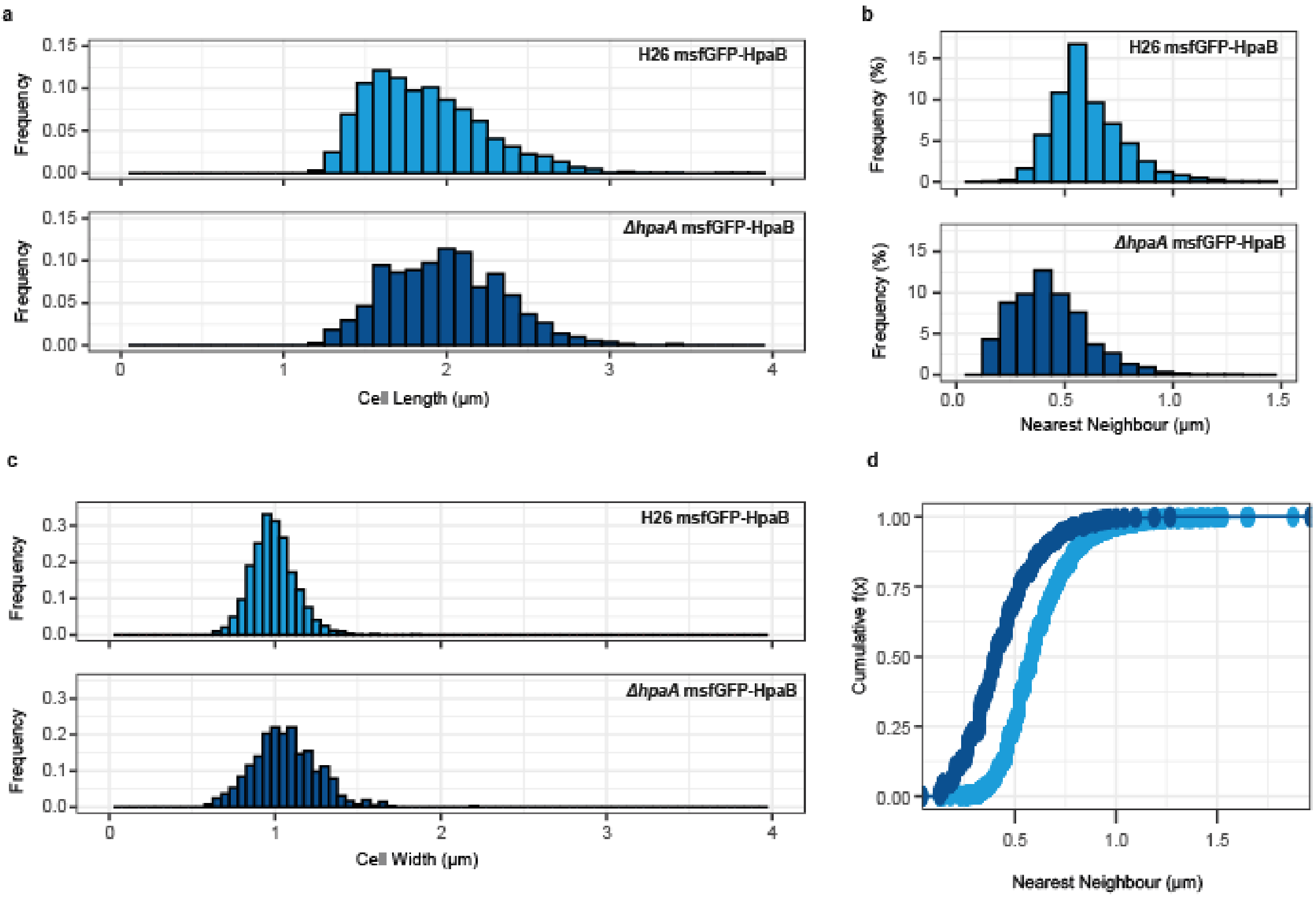

Supplementary figure 10: Cell parameters showing a difference in foci behaviour of HpaB-msfGFP in absence of HpaA.

Differences in cell length (a), and cell width (b) between the two strains represented as histograms. (c) Nearest neighbour distances for each focus in all cells represented as a histogram showing a left shift in absence of HpaA indicating increased closeness between foci for any given HpaB-msfGFP focus. (d) Cumulative distance function of the foci showing a left shift in absence of HpaA. ECDF if used further for G function.

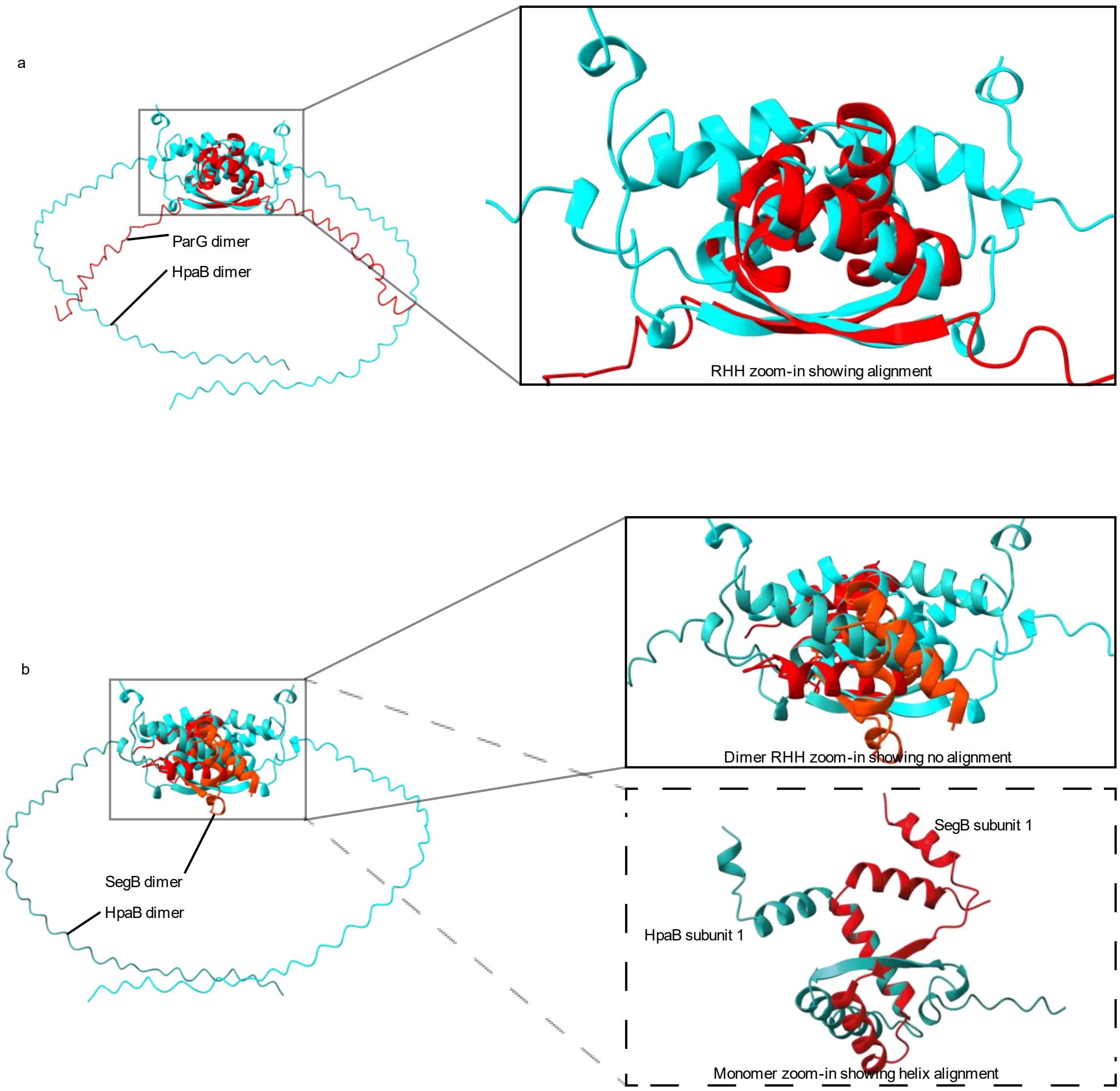

Supplementary figure 11: HpaB contains a conserved RHH fold

Comparison of Alphafold 3 predicted structure of HpaB in cyan with the known structures of ParG (PDB: 1P94)[1] and SegB (PDB: 7DUV)[2] in red. (A) Comparison with ParG with inset zoomed-in RHH motif shows similar RHH fold overlap. (B) Comparison with SegB with inset zoomed-in dimer and monomer showing overlapping helix from the RHH motif.

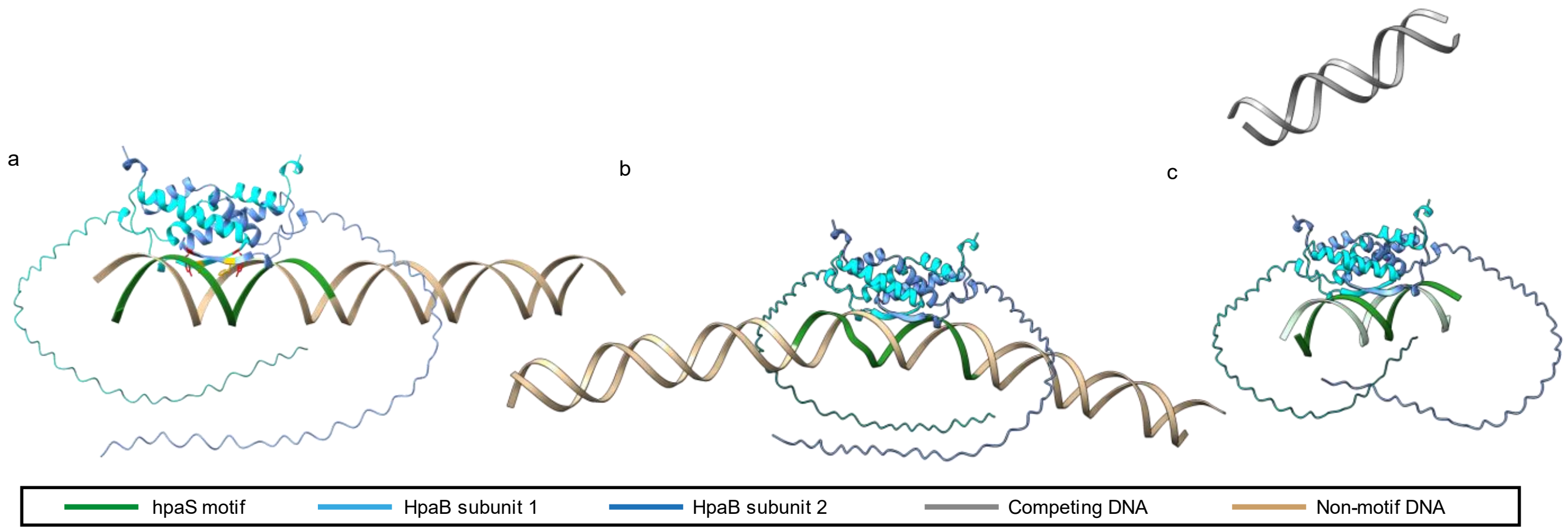

Supplementary figure 12: HpaB prefers the *hpaS3* motif for binding

Comparison of Alphafold 3 predictions of HpaB dimer in cyan with different dsDNA. (a) *hpaS3* (green) on the end of a DNA strand (beige), (b) *hpaS3* in the middle of a DNA strand, and (c) *hpaS3* competing with a similar sized scrambled sequence (grey).

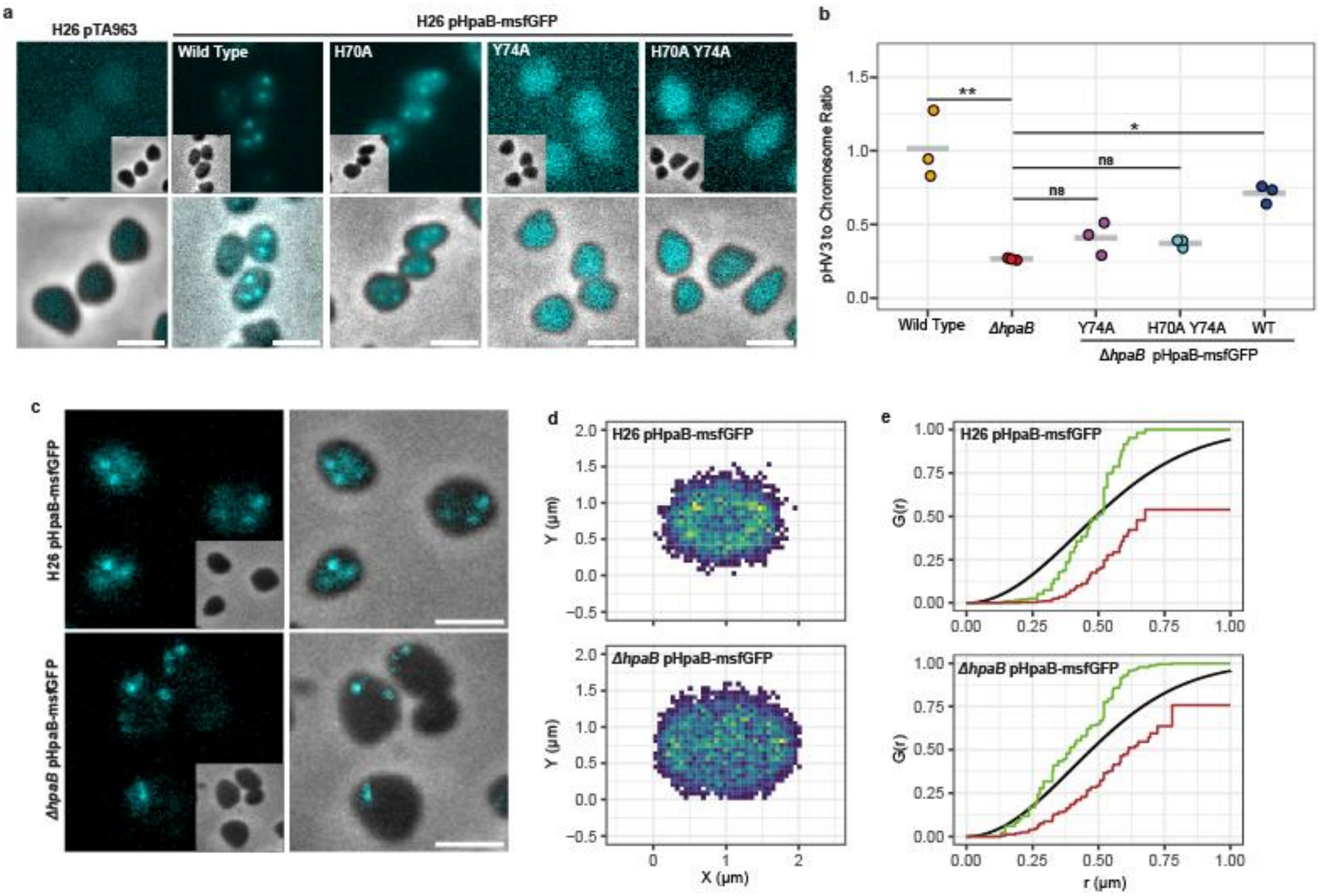

Supplementary figure 13: HpaB binds DNA via RHH motif, and HpaA via N terminus

(a) Widefield fluorescence microscopy showing representative cells with fluorescence of HpaB variants tagged with msfGFP in wild type cells showing foci and point mutation variants lacking foci. Scale bar = 2 µm. (b) qPCR assays performed on genomic DNA of *H. volcanii* showing the partial complementation of pHV3 to chromosome ratio by over-expression of WT HpaB, and no complementation by HpaB variants. Stars indicate significance: ** (<0.01), *(<0.05), calculated using two-sided Kruskal-Wallis Test. (c) Widefield fluorescence microscopy showing representative cells with fluorescent foci of $HpaB_{\Delta N}$ tagged with msfGFP (cyan) in wild type cells and cells lacking *hpaB*. Scale bar indicates 2 µm. (d) The distribution heatmap of foci within all the analysed cells centred using X/Y coordinates showing an average likelihood of foci distribution in cells. (e) G-function curve with border corrected nearest neighbour cumulative curves using Hanisch (green) and km estimates (brown). The black curve indicates theoretical randomness on the given set of data; the corrected curves show that the HpaB foci are non-randomly distributed in an N-terminus dependent manner. p value < 1e-16, n_{WT} = 620 cells, $n_{\Delta N}$ = 523 cells.

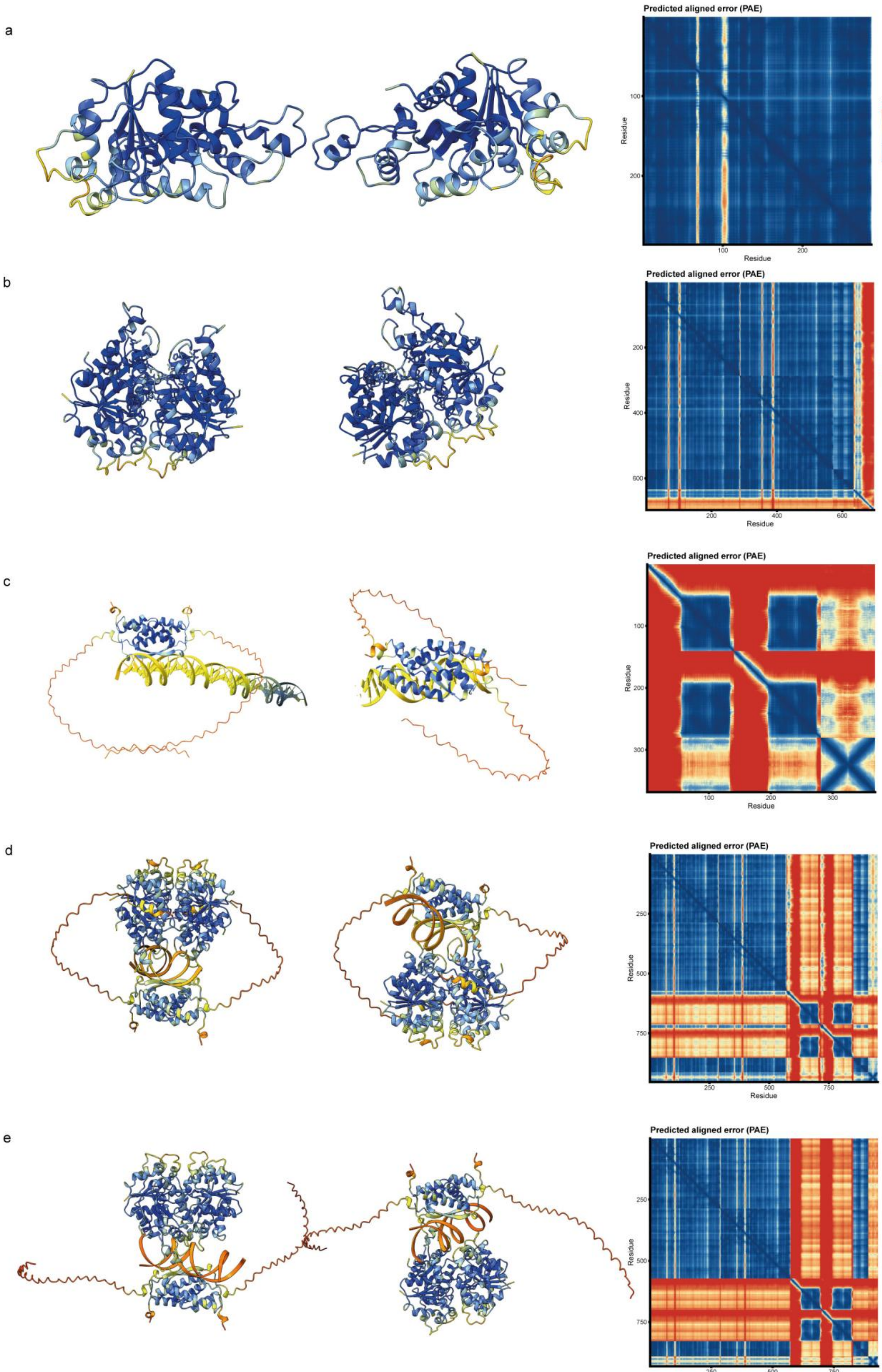

a
Predicted aligned error (PAE)
PAE (Å)
Residue
b
Predicted aligned error (PAE)
PAE (Å)
Residue
c
Predicted aligned error (PAE)
PAE (Å)
Residue
d
Predicted aligned error (PAE)
PAE (Å)
Residue
e
Predicted aligned error (PAE)
PAE (Å)
Residue

Supplementary figure 14: Alphafold structures with pLDDT scores and predicted alignment errors

(a) HpaA monomer, (b) HpaA dimer with ATP, (c) HpaB dimer with DNA, (d) HpaB dimer with HpaA dimer and DNA, (e) HpaB Δ*N12* dimer with DNA, all in two different angles colored according to plDDT scores, with their corresponding PAE matrices to the right. pLDDT legend: dark blue: pLDDT > 90, light blue: 90 >pLDDT > 70, yellow: 70> pLDDT > 50, orange: pLDDT < 50**.** Uniprot sequence accessions: HpaB D4GP18, HpaA D4GP19**.** Sequence for truncated HpaB is in source data file.

Supplementary table 1: Genes up/downstream of *hpaS* sites

Gene Annotation	Description	Up/Downstream
HVO_B0019	Putative anion symporter	Downstream Forward
HVO_B0113	Putative oxidoreductase	Upstream Forward
HVO_B0114	Transcription regulator RhcR	Downstream Forward
HVO_B0172	Hypothetical protein	Downstream Reverse
HVO_B0173	SMC-like protein Sph4	Upstream Reverse
HVO_B0198	ABC-type transport system periplasmic substrate-binding protein	Downstream Reverse
HVO_B0199	Deaminase domain protein	Downstream Forward
HVO_B0226	Auxin permease family transport protein	Downstream Reverse
HVO_B0227	ABC-type transport system ATP-binding protein	Upstream Reverse
HVO_B0267	Peptidase M24 family protein	Downstream Reverse
HVO_B0268	Monooxygenase	Downstream Forward

Supplementary table 2: Statistics Values

**qPCR comparisons between HpaA-msfGFP variants for Coefficient of Variance – Supplementary Figure 3a**		
**Comparison**	**p-value (significance)** [a]	**VDA.m (eff. size)** [b]
HpaA/HpaA$_{G12V}$	7.7e-36(****)	0.759 (large)-
HpaA/HpaA$_{K16A}$	ns	-
HpaA/HpaA$_{D40A}$	ns	-
HpaA$_{K16A}$/HpaA$_{G12V}$	5.9e-44(****)	0.721 (large)

$HpaA_{D40A}/HpaA_{G12V}$	5.4e-21(****)	0.784 (large)
$HpaA_{K16A}/HpaA_{D40A}$	ns	-

[a] Adjusted p value and associated significance levels: non-significant (ns), <0.05 (*), <0.01 (**), <0.001 (***), <0.0001(****)
[b] Vargha and Delaney's A and associated effect size: VDA<0.56 (-), 0.56≤VDA<0.64 (small), 0.64≤VDA<0.71 (moderate), 0.64≤VDA (large)

qPCR comparisons between HpaA-msfGFP variants for PeakRatio – Supplementary Figure 3b

Comparison	p-value (significance) [a]	VDA.m (eff. size) [b]
$HpaA/HpaA_{G12V}$	1.6e-50(****)	0.816 (large)-
$HpaA/HpaA_{K16A}$	3.8e-16(****)	0.64 (moderate)
$HpaA/HpaA_{D40A}$	0.752(ns)	-
$HpaA_{K16A}/HpaA_{G12V}$	3.5e-117(****)	0.869 (large)
$HpaA_{D40A}/HpaA_{G12V}$	2.8e-27(****)	0.829 (large)
$HpaA_{K16A}/HpaA_{D40A}$	9.4e-9(****)	0.651 (moderate)

[a] Adjusted p value and associated significance levels: non-significant (ns), <0.05 (*), <0.01 (**), <0.001 (***), <0.0001(****)
[b] Vargha and Delaney's A and associated effect size: VDA<0.56 (-), 0.56≤VDA<0.64 (small), 0.64≤VDA<0.71 (moderate), 0.64≤VDA (large)

qPCR comparisons between wild type, Δ*hpaA,* Δ*hpaB,* and complements – Figure 4c

Comparison	p-value (significance) [a]	VDA.m (eff. size) [b]
H26_Cas/H26_Xyl1	0.7158 (ns)	-
H26_Cas/H26_Xyl2	0.0180 (*)	0.739 (large)
H26_Xyl1/H26_Xyl2	0.3493 (ns)	-

H26_Cas/ Δ*hpaB*_Cas	5.74e−14 (****)	1.000 (large)
H26_Cas/ Δ*hpaB*_Xyl1	1 (ns)	-
H26_Cas/ Δ*hpaB*_Xyl2	1 (ns)	-
H26_Cas/ Δ*hpaB*_comp_Cas	0.0049 (**)	0.9676 (large)
H26_Cas/ Δ*hpaB*_comp_Xyl1	0.07 (ns)	-
H26_Cas/ Δ*hpaB*_comp_Xyl2	1 (ns)	-
H26_Cas/ Δ*hpaA*_Cas	0.0168 (*)	0.996 (large)
H26_Cas/ Δ*hpaA*_Xyl1	0.0377 (*)	1 (large)
H26_Cas/ Δ*hpaA*_Xyl2	1 (ns)	-
H26_Cas/ Δ*hpaA*_comp_Cas	1 (ns)	-
H26_Cas/ Δ*hpaA*_comp_Xyl1	0.1062 (ns)	-
H26_Cas/ Δ*hpaA*_comp_Xyl2	0.1917 (ns)	-

[a] Adjusted p value and associated significance levels: non-significant (ns), <0.05 (*), <0.01 (**), <0.001 (***), <0.0001(****)

[b] Vargha and Delaney's A and associated effect size: VDA<0.56 (-), 0.56≤VDA<0.64 (small), 0.64≤VDA<0.71 (moderate), 0.64≤VDA (large)

**qPCR comparisons between wild type, Δ*hpaB,* and complements – Supplementary figure 12b**		
**Comparison**	**p-value (significance) [a]**	**C. d (eff. size) [b]**
H26/ Δ*hpaB*	0.0014 (**)	-4.59 (Huge)
H26/ Δ*hpaB*_c($HpaB_{WT}$)	0.0783(ns)	–
H26/ Δ*hpaB*_c($HpaB_{Y74A}$)	0.0058(**)	-3.34 (Huge)
H26/ Δ*hpaB*_c($HpaB_{H70A\_Y74A}$)	0.0026(**)	-3.91 (Huge)
Δ*hpaB* / Δ*hpaB*_c($HpaB_{WT}$)	0.0179 (*)	9.82 (Huge)
Δ*hpaB* / Δ*hpaB*_c($HpaB_{Y74A}$)	0.5001 (ns)	-
Δ*hpaB* / Δ*hpaB*_c($HpaB_{H70A\_Y74A}$)	0.6164(ns)	-

[a] Adjusted p value and associated significance levels: non-significant (ns), <0.05 (*), <0.01 (**), <0.001 (***), <0.0001(****)
[b] Cohen's d effect size: 0.2>C d (-), 0.2<C d<0.5 (Small), 0.5<C d<0.8 (Medium), 0.8≤C d<1.2 (Large), 1.2≤C d<2 (Very Large), 2≤C d (Huge)."-" indicates directionality

Supplementary table 3: Strains used in this study

**Strain Name**	**Description**	**Reference**
	Haloferax volcanii	
	H26 Δ*pyrE2*	[3]
HPHfx007	H26 Δ*pyrE2* Δ*hpaB* (pop–out)	This study
HPHfx010	H26 Δ*pyrE2* Δ*hpaB* pTA963 HpaB	This study
HPHfx011	H26 Δ*pyrE2* Δ*hpaB* pTA963 HpaB–msfGFP	This study
HPHfx014	H26 Δ*pyrE2* pTA963 HpaB–msfGFP	This study
HPHfx017	H26 Δ*pyrE2* Δ*hpaB* pTA963 HpaA–msfGFP	This study
HPHfx019	H26 Δ*pyrE2* pTA963 HpaA–msfGFP	This study
HPHfx028	H26 Δ*pyrE2* Δ*hpaA* (pop–out)	This study
HPHfx031	H26 Δ*pyrE2* Δ*hpaA* pTA963 HpaB–msfGFP	This study
HPHfx032	H26 Δ*pyrE2* Δ*hpaA* pTA963 HpaA–msfGFP	This study
HPHfx106	H26 Δ*pyrE2* Δ*hpaB* pTA963 (empty vector)	This study
HPHfx107	H26 Δ*pyrE2* Δ*hpaA* pTA963 (empty vector)	This study
HPHfx108	H26 Δ*pyrE2* Δ*hpaB* pTA963 HpaA	This study
HKP003	H26 Δ*pyrE2* pHVID HpaA–mTurqoise2 HpaB–mScarlet–I	This Study
HKP005	H26 Δ*pyrE2* pTA963 HpaA–Halo	This study
HKP007	H26 Δ*pyrE2* Δ*hpaB* pTA963 HpaA–Halo	This study
HKP010	H26 Δ*pyrE2* pTA963 Δ–msfGFP H70A	This study
HKP015	H26 Δ*pyrE2* pTA963 HpaB–msfGFP Y74A	This study
HKP016	H26 Δ*pyrE2* pTA963 HpaB–msfGFP H70A Y74A	This study
HKP025	H26 Δ*pyrE2* Δ*hpaB* pTA963 HpaB–msfGFP H70A Y74A	This study
HKP026	H26 Δ*pyrE2* Δ*hpaB* pTA963 HpaB–msfGFP Y74A	This study

HKP030	H26 Δ*pyrE2* pTA963 HpaB−msfGFP ΔN−term (−A12)	This study
HKP031	H26 Δ*pyrE2* Δ*hpaB* pTA963 HpaB−msfGFP ΔN−term (−A12)	This study
HKP041	H26 Δ*pyrE2* Δ*hpaA* pTA963 HpaA−msfGFP G12V	This study
HKP042	H26 Δ*pyrE2* Δ*hpaA* pTA963 HpaA−msfGFP K16A	This study
HKP043	H26 Δ*pyrE2* Δ*hpaA* pTA963 HpaA−msfGFP D40A	This study
HKP051	H26 Δ*pyrE2* pTA963 msfGFP-HpaB	This Study
HKP052	H26 Δ*pyrE2* Δ*hpaB* pTA963 msfGFP-HpaB	This Study
HKP053	H26 Δ*pyrE2* Δ*hpaA* pTA963 msfGFP-HpaB	This Study
HKP054	H26 Δ*pyrE2* pTA963 msfGFP-HpaB H70A	This Study
HKP055	H26 Δ*pyrE2* pTA963 msfGFP-HpaB H70A Y74A	This Study
HKP056	H26 Δ*pyrE2* pTA963 msfGFP-HpaB ΔN-term 2-12	This Study
HKP057	H26 Δ*pyrE2* Δ*hpaB* pTA963 msfGFP-HpaB ΔN-term 2-12	This Study

Supplementary table 4: Plasmids used in this study

Plasmid Name	Description	Reference
pTA963		[4]
	pTA963 HpaA	This study
	pTA963 HpaB	This study
	pTA963 HpaB–msfGFP	This study
	pTA963 $HpaB_{H70A}$–msfGFP	This study
	pTA963 $HpaB_{Y74A}$–msfGFP	This study
	pTA963 $HpaB_{H70A\ Y74A}$–msfGFP	This study
	pTA963 $HpaB_{\Delta N\text{–}term}$–msfGFP	This study
	pTA963 msfGFP-HpaB	This study
	pTA963 msfGFP-$HpaB_{H70A}$	This study
	pTA963 msfGFP-$HpaB_{H70AY74A}$	This study
	pTA963 msfGFP-$HpaB_{\Delta N\text{–}term}$	This study
	pTA963 HpaA–msfGFP	This study
	pTA963 HpaA–halotag	This study
	pTA963 $HpaA_{G12V}$–msfGFP	This study
	pTA963 $HpaA_{K16A}$–msfGFP	This study
	pTA963 $HpaA_{D40A}$–msfGFP	This study
pTA131 BEMCS		[5]
	pTA 131 BEMCS Δ*hpaB*	This study
	pTA 131 BEMCS Δ*hpaA*	This study
pHVID 2		[6]
pHVID 7		
pHVID 9		
	pHVID HpaB–mScarlet–I HpaA–mTurqoise2	This study

Supplementary table 5: Oligonucleotides used in this study

Description	Sequence
HVO_B0017frg1_s	CGC TTC TCG GGG ACG ACT ACG
HVO_B0017frg1_as	GGA TTA TCC GAA GGT CGC CGA AGG GGT CTT CGC
HVO_B0017frg2_s	TCG GCG ACC TTC GGA TAA TCC ACC TCC GTC G
HVO_B0017frg2_as	GTA CGC GAC CAT GAG GAG TTC G
HVO_B0017s_BspH1	TGG CCT CAT GAG CGA AGA CCC C
HVO_B0017as_BamH1	GTC CGG GGA TCC GAG TTT CGA CGG
HVO_B0017as_+3linker	GGA CCC TCC GCC ACC GCT ACC CCC GCC ACC TTC GGA ATC TTC GTC GTC GAC
HVO_A0002as_+3linker	GGA CCC TCC GCC ACC GCT ACC CCC GCC ACC CTC GTT GCG ACG TTC GAG GAC
HVO_A0459as_+3linker	GGA CCC TCC GCC ACC GCT ACC CCC GCC ACC GTC GAA CGT CAT CCC GTA GCC
msfGFP_s+5linker	GGT GGC GGG GGT AGC GGT GGC GGA GGG TCC ATG GGT ACC CTG CAG ATG AG
msfGFP_as+3BamHI	CCA CGA GGA TCC CTA TTT GTA GAG CTC ATC CAT GC
T3	AAT TAA CCC TCA CTA AAG GG
T7	TAA TAC GAC TCA CTA TAG GG
msfGFP_NcoI_s	TCG TGG CCA TGG GTA CCC TGC AGA TGA G
HVO_B0018frg1_s	GAG GAG GAA GTA CAC GAG CGA GG
HVO_B0018frg1_as	GAC GAT TTG GAT GGG GAT ATC CGT ACT ATC G
HVO_B0018frg2_s	GAT ATC CCC ATC CAA ATC GTC GAA GAA GGC GAG G
HVO_B0018frg2_as	GAC TTC TGT CCG GCG CTC CGA G
HVO_B0018s_Pci1	TCC CCA ACA TGT CAC GTG CAG TC
GSG−HaloTag_fwd	GGT GGC CCG AGG ATC GGG AGA AAT CGG TAC
GSG−HaloTag_rev	AGG CCC GCA CTT AGG AAA TCT CCA GAG TAG AC
pTA963_fwd	GAT TTC CTA AGT GCG GGC CTC TTC GCT ATT AC
pTA963_rev	CTC CCG ATC CTC GGG CCA CCT CGC CTT C
HVO_B0018as_+3linker	GGA CCC TCC GCC ACC GCT ACC CCC GCC ACC TCG GGC CAC CTC GCC TTC TTC
HpaB in mScarlet fw	ATACATATGAGCGAAGACCCCTTC
HpaB in mScarlet rev	TATGGATCCTTCGGAATCTTCGTCGTC

HpaA in mTq fw	ATACATATGTCACGTGCAGTCAGCG
HpaA in mTq rev	TATGGATCCTCGGGCCACCTCGCC
pTA963–Halo–Fw	GGT GGC GGG GGT AGC
pTA963–Halo–Rev	GTG GTG GTG GTG GTG G
pHVID_mTq_fwd<br>pHVID_mTq_rev	GGC CCG AGG CGG CGG GGG CTC GGG G<br>CGT GAC ATG GAT CCC ATA TGT CTA GAA GGT CCG CGA ATG TGA AGT A
HpaA_fwd	TGG GAT CCA TGT CAC GTG CAG TCA GCG TC
HpaA_rev	CCG CCG CCT CGG GCC ACC TCG CCT TC
pHVID_mScarletI_fwd	TTC CGA AGG CGG CGG GGG CTC GGG G
pHVID_mScarletI_rev	CGC TCA TGG ATC CCA TAT GTC TAG AAG GTC CGC GAA TGT GAA GTA
HpaB_fwd	TGG GAT CCA TGA GCG AAG ACC CCT TC
HpaB_rev	CCG CCG CCT TCG GAA TCT TCG TCG TC
HpaB H70A OE fw	GAGACCGTCGCCAAGGCGCTG
HpaB H70A OE rv	GCGCCTTGGCGACGGTCTCGTC
HpaB Y74A OE fw	GGCGCTGGCCGTGCTCGAAG
HpaB Y74A OE rv	CTTCGAGCACGGCCAGCGCCTTG
pTA963_OLEG_fw	AGATTCCGAAGGTGGCGGGGGTAGCGGT
pTA963_OLEG_rv	CTTCGCTCATGTGGTGGTGGTGGTGGTGC
HpaB_OLEG_fw	CCACCACCACATGAGCGAAGACCCCTTC
HpaB_OLEG_rv	CCCCGCCACCTTCGGAATCTTCGTCGTC
HpaB_ΔNTerm_Fw	ATG GGA AGC GGC GAG

Supplementary References

1 Golovanov, A. P., Barilla, D., Golovanova, M., Hayes, F. & Lian, L. Y. ParG, a protein required for active partition of bacterial plasmids, has a dimeric ribbon-helix-helix structure. *Mol Microbiol* **50**, 1141-1153, doi:10.1046/j.1365-2958.2003.03750.x (2003).

2 Yen, C. Y. *et al.* Chromosome segregation in Archaea: SegA- and SegB-DNA complex structures provide insights into segrosome assembly. *Nucleic Acids Res* **49**, 13150-13164, doi:10.1093/nar/gkab1155 (2021).

3 Bitan-Banin, G., Ortenberg, R. & Mevarech, M. Development of a gene knockout system for the halophilic archaeon Haloferax volcanii by use of the pyrE gene. *J Bacteriol* **185**, 772-778, doi:10.1128/JB.185.3.772-778.2003 (2003).

4 Allers, T., Barak, S., Liddell, S., Wardell, K. & Mevarech, M. Improved strains and plasmid vectors for conditional overexpression of His-tagged proteins in Haloferax volcanii. *Appl Environ Microbiol* **76**, 1759-1769, doi:10.1128/AEM.02670-09 (2010).

5 Allers, T., Ngo, H. P., Mevarech, M. & Lloyd, R. G. Development of additional selectable markers for the halophilic archaeon Haloferax volcanii based on the leuB and trpA genes. *Appl Environ Microbiol* **70**, 943-953, doi:10.1128/AEM.70.2.943-953.2004 (2004).

6 Ithurbide, S., de Silva, R. T., Brown, H. J., Shinde, V. & Duggin, I. G. A vector system for single and tandem expression of cloned genes and multi-colour fluorescent tagging in Haloferax volcanii. *Microbiology (Reading)* **170**, doi:10.1099/mic.0.001461 (2024).
