# Supplementary material for: Equipositioning of chromosomes in the polyploid archaeon Haloferax volcanii by HpaAB
Source: Nat Commun. 2026 Oct 30;17:11296. doi: 10.1038/s41467-026-78993-w (PMC13722703; doi:10.1038/s41467-026-78993-w)
Supplement: Supplementary file 2 [file 41467_2026_78993_MOESM2_ESM.pdf]

nature portfolio

Corresponding author(s): Prof. Dr. Marc Bramkamp

Last updated by author(s): Sep 9, 2026

Reporting Summary

Nature Portfolio wishes to improve the reproducibility of the work that we publish. This form provides structure for consistency and transparency in reporting. For further information on Nature Portfolio policies, see our Editorial Policies and the Editorial Policy Checklist.

Statistics

For all statistical analyses, confirm that the following items are present in the figure legend, table legend, main text, or Methods section.

n/a	Confirmed	
☐	☒	The exact sample size (n) for each experimental group/condition, given as a discrete number and unit of measurement
☐	☒	A statement on whether measurements were taken from distinct samples or whether the same sample was measured repeatedly
☐	☒	The statistical test(s) used AND whether they are one- or two-sided<br>*Only common tests should be described solely by name; describe more complex techniques in the Methods section.*
☒	☐	A description of all covariates tested
☐	☒	A description of any assumptions or corrections, such as tests of normality and adjustment for multiple comparisons
☐	☒	A full description of the statistical parameters including central tendency (e.g. means) or other basic estimates (e.g. regression coefficient) AND variation (e.g. standard deviation) or associated estimates of uncertainty (e.g. confidence intervals)
☐	☒	For null hypothesis testing, the test statistic (e.g. F, t, r) with confidence intervals, effect sizes, degrees of freedom and P value noted<br>*Give P values as exact values whenever suitable.*
☒	☐	For Bayesian analysis, information on the choice of priors and Markov chain Monte Carlo settings
☒	☐	For hierarchical and complex designs, identification of the appropriate level for tests and full reporting of outcomes
☐	☒	Estimates of effect sizes (e.g. Cohen's d, Pearson's r), indicating how they were calculated

Our web collection on statistics for biologists contains articles on many of the points above.

Software and code

Policy information about availability of computer code

Data collection	Protein sequences, and Haloferax genome were retrieved from NCBI database as of October 2025. Phase contrast and fluorescent images were acquired using the Zen Blue 3.3.89 software, SPT images were acquired using Zen 3.0 Black software. qPCR data was acquired using Agilent Aria software 1.8, ChIP data was acquired using Novaseq Control software 1.8 and Illumina bcl2fastq 2.2.0 software
Data analysis	Protein structure prediction was done using Alphafold 3 server, followed by UCSF ChimeraX 1.9 for visualization. Protein alignment was done using Clustal Omega 1.2.4. Image analysis was performed using FIJI 2.17, MicrobeJ 5.13I, SMTracker 2.0 followed by visualization and statistics using RStudio 4.5 using the packages ggplot2 4.0.1, ggnewscale 0.5.2, patchwork 1.3.2, spatstat 3.4-1, rstatix 0.7.3, dplyr 1.4.4, effsize 0.8.1. ChIP sequence data was analyzed using Galaxy web platform, and visualized using IGV 2.19.4.

For manuscripts utilizing custom algorithms or software that are central to the research but not yet described in published literature, software must be made available to editors and reviewers. We strongly encourage code deposition in a community repository (e.g. GitHub). See the Nature Portfolio guidelines for submitting code & software for further information.

Data

Policy information about availability of data

All manuscripts must include a data availability statement. This statement should provide the following information, where applicable:

- Accession codes, unique identifiers, or web links for publicly available datasets
- A description of any restrictions on data availability
- For clinical datasets or third party data, please ensure that the statement adheres to our policy

All data supporting the findings of this study are available within the article and its Supplementary Information. Source data underlying the graphs and quantitative analyses are provided with this paper in the Source Data file. The ChIP-seq data generated in this study have been deposited in ENA under accession numbers PRJEB125767 (publically available) and PRJEB113869 (same data currently suppressed, due to be released soon). Any additional data generated during the current study are available from the corresponding author upon request.

Research involving human participants, their data, or biological material

Policy information about studies with human participants or human data. See also policy information about sex, gender (identity/presentation), and sexual orientation and race, ethnicity and racism.

Reporting on sex and gender	*Use the terms sex (biological attribute) and gender (shaped by social and cultural circumstances) carefully in order to avoid confusing both terms. Indicate if findings apply to only one sex or gender; describe whether sex and gender were considered in study design; whether sex and/or gender was determined based on self-reporting or assigned and methods used.*<br>*Provide in the source data disaggregated sex and gender data, where this information has been collected, and if consent has been obtained for sharing of individual-level data; provide overall numbers in this Reporting Summary. Please state if this information has not been collected.*<br>*Report sex- and gender-based analyses where performed, justify reasons for lack of sex- and gender-based analysis.*
Reporting on race, ethnicity, or other socially relevant groupings	*Please specify the socially constructed or socially relevant categorization variable(s) used in your manuscript and explain why they were used. Please note that such variables should not be used as proxies for other socially constructed/relevant variables (for example, race or ethnicity should not be used as a proxy for socioeconomic status).*<br>*Provide clear definitions of the relevant terms used, how they were provided (by the participants/respondents, the researchers, or third parties), and the method(s) used to classify people into the different categories (e.g. self-report, census or administrative data, social media data, etc.)*<br>*Please provide details about how you controlled for confounding variables in your analyses.*
Population characteristics	*Describe the covariate-relevant population characteristics of the human research participants (e.g. age, genotypic information, past and current diagnosis and treatment categories). If you filled out the behavioural & social sciences study design questions and have nothing to add here, write "See above."*
Recruitment	*Describe how participants were recruited. Outline any potential self-selection bias or other biases that may be present and how these are likely to impact results.*
Ethics oversight	*Identify the organization(s) that approved the study protocol.*

Note that full information on the approval of the study protocol must also be provided in the manuscript.

Field-specific reporting

Please select the one below that is the best fit for your research. If you are not sure, read the appropriate sections before making your selection.

☒ Life sciences ☐ Behavioural & social sciences ☐ Ecological, evolutionary & environmental sciences

For a reference copy of the document with all sections, see nature.com/documents/nr-reporting-summary-flat.pdf

Life sciences study design

All studies must disclose on these points even when the disclosure is negative.

Sample size	Most experiments were done in at least biological triplicates. ChIP data was done in a biological duplicate. Sample sizes for single cell experiments or analyses were always n > 200. Sample sizes were chosen based on standards in the field and were not determined using statistical methods.
Data exclusions	We did not exclude datapoints. A source data file with all raw data is submitted along with this paper. Potential confounding variables were controlled through standardized experimental conditions rather than statistical adjustment for covariates.
Replication	Number of tecnical or biological replications are indicated in the text. We did not observe severe differences between experimental replicates. Experiments were controlled by using identical experimental conditions, buffers and solutions as well as identically treated samples. Identical phenotypes of strains were oberserved in independent experiments. Attempts at replication were successful
Randomization	Samples were not randomized because it was not necessary.

Blinding	Blinding was not necessary since we relied on automated imaging and statistical data evaluation

Reporting for specific materials, systems and methods

We require information from authors about some types of materials, experimental systems and methods used in many studies. Here, indicate whether each material, system or method listed is relevant to your study. If you are not sure if a list item applies to your research, read the appropriate section before selecting a response.

Materials & experimental systems

n/a	Involved in the study
x	Antibodies
x	Eukaryotic cell lines
x	Palaeontology and archaeology
x	Animals and other organisms
x	Clinical data
x	Dual use research of concern
x	Plants

Methods

n/a	Involved in the study
	x ChIP-seq
x	Flow cytometry
x	MRI-based neuroimaging

Plants

Seed stocks	*Report on the source of all seed stocks or other plant material used. If applicable, state the seed stock centre and catalogue number. If plant specimens were collected from the field, describe the collection location, date and sampling procedures.*
Novel plant genotypes	*Describe the methods by which all novel plant genotypes were produced. This includes those generated by transgenic approaches, gene editing, chemical/radiation-based mutagenesis and hybridization. For transgenic lines, describe the transformation method, the number of independent lines analyzed and the generation upon which experiments were performed. For gene-edited lines, describe the editor used, the endogenous sequence targeted for editing, the targeting guide RNA sequence (if applicable) and how the editor was applied.*
Authentication	*Describe any authentication procedures for each seed stock used or novel genotype generated. Describe any experiments used to assess the effect of a mutation and, where applicable, how potential secondary effects (e.g. second site T-DNA insertions, mosiacism, off-target gene editing) were examined.*

ChIP-seq

Data deposition

- [x] Confirm that both raw and final processed data have been deposited in a public database such as GEO.
- [ ] Confirm that you have deposited or provided access to graph files (e.g. BED files) for the called peaks.

Data access links<br>*May remain private before publication.*	https://www.ebi.ac.uk/ena/browser/home Accession number: PRJEB125767 (Public)<br>Alternate accession: https://www.ebi.ac.uk/ena/browser/home Accession number: PRJEB113869 (currently suppressed, due to be released soon)<br>Final graphs are given in the manuscript and can be requested from the corresponding authors upon request.
Files in database submission	Files for PRJEB125767 "Anti-6xHis ChIP seq of HpaB from Haloferax volcanii"<br><br>ERR17851147 - p6his-HpaB reads<br>ERR17851148 - p6His Empty vector reads<br><br>Files for PRJEB113869 "Anti-6xHis ChIP seq of HpaB from Haloferax volcanii"<br><br>ERR17354094 - p6his-HpaB reads<br>ERR17354093 - p6His Empty vector reads
Genome browser session<br>(e.g. UCSC)	no longer applicable

Methodology

Replicates	Experiments were done in biological duplicates
Sequencing depth	Data were generated by illumina sequencing using 2x150 paired-end configuration. 65 million reads were obtained for the experiment.

Antibodies	monoclonal Anti-HIS magnetic beads (Sigma Aldrich) #SAE0222. Lot 0000428748
Peak calling parameters	bamCoverage was used for read mapping. Count per million was used and compared with control to verify enrichment. MACS2 program was used for peak calling in the galaxy browser
Data quality	We only considered MACS2-called peaks with a log 2 enrichment more than 2.3, which were further visually inspected using IGV.
Software	usegalaxy.org, bowtie2 2.5.5 (Langmead and Salzberg, 2012 https://doi.org/10.1186/gb-2009-10-3-r25) was used to map reads against reference genome, and deep tool bamcompare 4.5.4 (Ramirez et al, 2016 https://doi.org/10.1093/nar/gkw257) was used for normalization and binning of data.<br>ChIP-seq profiles were plotted with x axis representing genomic positions and the y axis representing count per million reads.
