# Supplementary material for: Equipositioning of chromosomes in the polyploid archaeon Haloferax volcanii by HpaAB
Source: Nat Commun. 2026 Oct 30;17:11296. doi: 10.1038/s41467-026-78993-w (PMC13722703; doi:10.1038/s41467-026-78993-w)
Supplement: Supplementary file 3 [file 41467_2026_78993_MOESM3_ESM.pdf]

nature portfolio

Peer Review File

Equipositioning of Chromosomes in the Polyploid Archaeon *Haloferax volcanii* by HpaAB

Corresponding Author: Professor Marc Bramkamp

This file contains all reviewer reports in order by version, followed by all author rebuttals in order by version.

Version 0:

Reviewer comments:

Reviewer #1

(Remarks to the Author)
Summary:

Haloferax volcanii is a polyploid archaeon with a main chromosome and three secondary chromosomes maintained at high copy number depending on the growth phase. Due to this high copy number, genomic segregation thus far has been considered to be random. Authors have successfully identified a two-gene operon and characterised the genes, hpaA and hpaB, with a role in segregation and equipositioning of the chromosome replicons. The study is focusing specifically on the secondary chromosome, pHV3. pHV3 encodes all genes necessary for xylose uptake and metabolism, and deletion of either hpaA or hpaB causes severe growth defects on xylose. In the HpaAB system, HpaA is a ParA-like ATPase that forms an oscillating gradient in the cytosol, and HpaB is a novel archaeal DNA-binding protein. HpaB forms discrete intracellular foci via a ribbon–helix–helix motif; mutation of a conserved tyrosine residue abolishes foci formation and pHV3 maintenance. Loss of hpaB leads to reduced pHV3: main chromosome ratio, indicating a defect in segregation and severe growth defect on xylose. In the absence of HpaA, HpaB foci become numerous and randomly distributed, indicating its importance in an orderly, equidistant positioning of HpaB-bound plasmids.

Together, these findings demonstrate that the HpaAB system functions as an equipositioning system instead of a classical ParAB system. The HpaAB system bridges bacterial ParABS and McdAB-like positioning systems, illustrating how horizontal gene transfer and archaeal-specific adaptations can give rise to hybrid mechanisms. This study provides first evidence of an active organisational system (albeit currently demonstrated for pHV3) to be in place in the highly polyploid H.volcanii.

Here are some suggestions that could aid in the flow and readability of the paper:

Major suggestion :
1. Given that cell morphology and ploidy in H. volcanii vary significantly with growth phase, it is important to specify in the figure legend/result section the growth stage at which fluorescence microscopy data were collected.
1.1: Were wild-type and mutant cells imaged at identical time points? If so, presenting representative cells with comparable morphology across panels would improve clarity and facilitate interpretation of the localization data (Figure 2a).
If not, differences in cell shape may confound interpretation of the localization patterns (2b). Especially since line 356 mentioned HpaA oscillation being predominant in rod shaped cells. However, the mutants shown in the panel have no rod cell representation.
o 1.2: A supplementary video is provided for HpaA, are comparable movies available for all mutants? Alternatively, a cumulative kymograph summarizing multiple cells would be more representative than a single-cell examplesingle-cell example
2. The brightness/contrast used for the images in Figure 2a appears to be different for every panel. Using the consistent settings would allow readers to visualise the difference in fluorescence intensity better.
3. Page 14, lines 391-394 and Figures 2 and 3: While Figure 3 clearly shows Foci formation, the same observation is less apparent in Figure 2. The HpaA-msfGFP signal in Figure 2 looks compartmentalised /diffused rather than as focal points in the cells. This may reflect imaging conditions, growth stage, or contrast settings and should be clarified.
4. page 17, line 491-492, the sentence describing the growth defect should already be clarified here already.
5. page 17, line 500, "..that the concentration of HpaA in the cell.." The sentence would require further experimental evidence

as support, or should be rephrased
6. HpaB is suggested as the primary driver of the pHV3 segregation. In Δ hpaA, HpaB foci distribution is affected, which in turn affects the segregation. Then how is it that the Δ hpaA is more viable (as seen in the glucose growth curve, Supp 2, and qPCR data, Fig 4?
o 6.1: if available, is there data to show redundancy between the HpaA protein (line 741)
7. Figures 6 and 7 can be potentially unified, and certain data can be moved to the supplementary section to improve the flow of the paper.
8. Page 24, lines 725 to 726, the discussion shifts from xylose-based experiments to glucose-based observations. Consider restructuring this section so that the rationale provided on line 730 is introduced earlier.
9. For all the fluorescently tagged proteins used in the study, a complementation assay would strengthen confidence that the tagged proteins are functional.

Minor suggestion :

10. Page 4, line 98: reference 52 doesn't mention histones in H. volcanii
11. Page 4, line 98: "…H. volcanii, coincides with the presence of histones 52, facilitating DNA compaction" DNA compaction has not yet been shown by the histone in H. volcanii; therefore, consider reframing the sentence. For reference: 10.1186/s13059-023-03095-5
12. Page 5, line 108: "..chromosome (1:1 to 1:1.2), remains poorly understood" Reference?
13. Page 5, line 123: Reference? "rods in early growth phases, and plates in late growth phases"
14. Page 14, line 394: "..absence of HpaA,.." in the absence of HpaB?
15. Page 18, line 528, Full length HpaB-msfGFP instead of Wild type.
16. Page 18, line 530, authors show mutation of Y74A disrupts foci formation ability of HpaB, in the result 5b panel 3 the image has been shown in high contrast which could be due to the high background signal. As a confirmation of expression and to verify the expression levels, western blot for the tagged mutants could be performed. The contrast and brightness of these panels should be maintained (similar as point 2)
17. Page 22, line 653, "..likely adaptation to the hypersaline.." Since this is known for Haloferax proteins, please add a reference.
18. Page 24, line 730 should also be mentioned in the results section already, for readers to understand the flow and reasoning to go from one experiment to another.
19. Page 25, line 741 "..redundancy between the HpaA proteins encoded in the H. volcanii genome" HpaA-like proteins? Since the other 3 genes are yet to be characterised? If not homologs, a justification for the sentence could provide more insight.
20. Ensure all microscopy images include both strain and fusion protein labels; avoid using "WT" to refer to proteins (e.g., use HpaA–msfGFP instead of WT in Figure 2a).
21. While in Line 287 p-values have been mentioned, in all figures with statistics, these values should also be specified in the legend and ensure to keep the numbers consistent throughout. Use "ns" instead of "-"
22. Please ensure to maintain the figure legends for every figure and the x- and y-axis labelling for graphs.
23. Statistical comparison should be shown of each mutant and complementation with respect to WT (H26). Please mention the gene/protein complemented in the figure itself (example for gene- hpaB::hpaB, if the complementation was without any tags)
24. Figure 4c should come before Figure 4b. This would also make sense for the flow of the paper.
25. Figure 4b legend for the growth curve has Δ OD.
26. Figure 5c, same for statistics as mentioned for Figure 4.
27. Keeping HpaA oscillatory behaviour in mind, is it possible that the qPCR results would have a difference when cells were collected from an earlier log phase (0.01-0.06), where more rods are observed, rather than the OD600 of 0.1(Page 9, line 254)?
28. Colours used in figure 7a,b are very similar to 7c, even though the data being shown is different. Additionally, a label for WT and mutants needs to be added to the figure.
29. Figure 7c needs to have the signal density scale bar.
30. Supp Figure 2: Complement the growth curve with cell shape images to follow phenotypic changes or a spot assay?
31. Live cell imaging with fluorescently tagged proteins and DNA dye to have visual evidence of the said segregation defect could add to the results already presented.
32. Although structural prediction and mutational analysis strongly suggest DNA binding by HpaB, direct biochemical evidence (e.g., EMSA, MST, Mass photometry) is missing.

While the study is missing some validations, the authors have integrated several techniques to cross-validate hypothesis and conclusions, with clear evidence of the function of the two previously uncharacterised proteins. The study is not only relevant for the field but also advances our knowledge of the genome organisation in the H.volcanii.

Reviewer #2

(Remarks to the Author)
Review of Premrajka et al., "Equipositioning of Chromosomes in the Polyploid Archaeon Haloferax volcanii"

This manuscript describes experiments aimed at understanding chromosome (technically secondary chromosome)

segregation in the Archaeon Haloferax volcanii. Based on similarity to bacterial DNA-partitioning protein ParA, the authors identify a gene, which they call hpaA, on one of the secondary chromosomes of Haloferax (pHV3). An adjacent (partially overlapping) gene, hpaB, is uncharacterized but the authors speculate that HpaA and HpaB might cooperate to promote DNA segregation by a mechanism similar to that of ParABS systems in bacteria. By fluorescence microscopy, fluorescent HpaA fusion proteins form discrete foci which, in rod-shaped cells, appear to spatially oscillate. HpaB forms foci that co-localize with HpaA, and loss of HpaB disrupts focus formation by HpaA. In addition, loss of HpaB (but not HpaA) skews the ratio of the secondary chromosome, pHV3, to the primary chromosome, suggesting a direct role in secondary chromosome maintenance. Cells lacking HpaA increase in size and contain more numerous HpaB foci. While the work is interesting and suggests that HpaAB may function in a similar manner to ParABS DNA segregation systems this conclusion is not well supported by the experiments presented.

Major points:

1. The title is misleading because the manuscript does not show any images of chromosome movement in live cells that prove HpaAB is required for equal distribution of chromosomes among daughter cells during division. Conclusions regarding chromosome organization are largely inferred from protein localization patterns rather than direct observation of chromosomes. The authors should either demonstrate equipositioning of chromosomes or change the title.

2. Fig. 2 presents localization data and evidence for oscillatory behavior of HpaA. However, it does not provide direct evidence for active or directed chromosome movement. It is unclear whether HpaA interacts nonspecifically with DNA, binds specific chromosomal regions, or associates with other segregation factors. The authors should include a panel of images showing chromosomal DNA (e.g., nucleoid staining or specifically labeled chromosomal loci) to contextualize the observed protein dynamics. Additionally, it would be great for the authors to comment on whether differences in localization patterns could arise from cell-cycle heterogeneity, as cells do not appear to be synchronized. Clarification on whether nucleoid compaction differs between wild-type and HpaA mutant strains would further strengthen the interpretation.

3. In Fig. 3 the altered HpaA dynamics observed in the absence of HpaB resemble those reported for ATP-binding or ATP-hydrolysis defective HpaA mutants. This raises the possibility that HpaB influences HpaA ATPase activity or stabilizes a specific nucleotide-bound state. The authors should comment on this possibility and (even better) perform in vitro experiments to directly test whether HpaB alters HpaA ATPase activity.

4. On l. 469 the authors claim that, “HpaA and HpaB are essential for growth on xylose,” This is clearly an overstatement! HpaA and B mutants exhibit growth defects but loss of either protein is clearly not lethal. This statement should be amended to something like: “HpaA and HpaB are both necessary for optimal growth on xylose”. Apart from xylose, did the authors get to see any growth defect with other common sugars like glucose? Did the authors test the double KO strain with both hpaA and hpaB deletion? Are they lethal?

5. The data in Fig. 5 should include DNA colocalisation as a control since protein foci can arise from aggregation, oligomerization, and/or phase separation. Without seeing the DNA, it is difficult to conclude that the observed foci represent chromosome-associated structures.

6. Also, related to Fig. 5 the authors claim that HpaA binds DNA but never demonstrate this fact. Analogy with ParA and AlphaFold predictions are not sufficient. At the very least, the authors should perform electrophoretic mobility shift analysis (EMSA) using purified HpaA before they say that it binds DNA.

7. In Fig. 6 no biochemical evidence is provided for a physical interaction between HpaA and HpaB. Also, without DNA colocalisation data it is impossible to determine whether HpaA controls HpaB distribution on DNA or whether loss of one protein alters localization of the other via more generic perturbations of cellular organization.

8. In Fig. 8, again conclusions regarding interactions between N-terminally truncated HpaB and HpaA are based on localization data (and analogy to bacterial ParAB systems). Biochemical or biophysical interaction assays are required to distinguish between a true loss of interaction and indirect effects on protein localization or stability resulting from N-terminal deletion. The AlphaFold analysis is suggestive but not definitive. None of the experiments in the paper rule out the possibility that HpaA and HpaB simply form aggregates or phase condensates, possibly induced by GFP labeling.

9. Conceptually, I am having a hard time understanding how inhibition of an equipartitioning system results in population-level depletion of the secondary chromosome pHV3. If the partitioning is simply unequal then the average should stay the same (at least for a while) but the variability in pHV3 ratio in the population should dramatically increase. If the secondary chromosome pHV3 is important for growth and survival I would expect cells with a lower copy number of this chromosome to be at a competitive disadvantage and that the ratio of pHV3 to primary chromosome might actually increase. I would expect the observed result only (or primarily) under conditions where increased pHV3 copy number is deleterious.

10. On ll. 504-506 the following sentences are not accurate: “Moreover, these defects could be rescued upon tryptophan-controlled expression of HpaA and HpaB via ectopic complementation constructs. Complementation of hpaB fully restored growth to wild-type levels.” The modest increase in cell density early in the growth curve for HpaA complementation does not appear to warrant the word “rescue”, especially given that the authors characterized the hpaA knockout cells as inviable.

11. Overall, the paper lacks sufficient statistical analysis and/or rigor. For example, in Fig. 2 how often did the authors observe oscillation of HpaA? What fraction of cells had diffuse localization or non-oscillating puncta? How many biological replicates were performed? How many cells analyzed from each replicate? This information should be provided for every

piece of quantitative data.

Minor Points:
1. Overall the figures should be labeled much better. For example, in Fig. 2 the authors should make clear that the individual kymograph images in 2b correspond to analysis of movies containing the images above, in 2a. At least I assume that they do. Also they should label on the figure which fluorescent fusion protein we are seeing.

2. In Fig. 2 the authors should mark which cell(s) in the image were used for the kymograph analysis. They should also mark the line along which the kymographs were computed. Also, the kymographs are not super convincing. The authors should include sequential images from the time laps movies illustrating the oscillatory behavior.

3. For the rescue and tryptophan-induced expression experiments the authors should show immunoblots of the expression levels. For example is the lack of “rescue” by HpaA simply due to low expression levels in those experiments?

4. In Fig. 4 I cannot tell whether the statistics were performed on biological or technical replicates.

Version 1:

Reviewer comments:

Reviewer #1

(Remarks to the Author)
The authors have satisfactorily addressed the experimental concerns raised during the first round of review. Addition of complementation assaya,ChIP data and restructuring of the result section has strengthened the mechanistic conclusion and greatly developed the flow. I beleive the manuscript is ready for publication

Minor suggestion: There are slight textual inconsistency that could be looked upon one last time

Reviewer #2

(Remarks to the Author)
The authors have adequately responded to all of my comments.

Open Access This Peer Review File is licensed under a Creative Commons Attribution 4.0 International License, which permits use, sharing, adaptation, distribution and reproduction in any medium or format, as long as you give appropriate credit to the original author(s) and the source, provide a link to the Creative Commons license, and indicate if changes were made.
In cases where reviewers are anonymous, credit should be given to 'Anonymous Referee' and the source.

The images or other third party material in this Peer Review File are included in the article's Creative Commons license, unless indicated otherwise in a credit line to the material. If material is not included in the article's Creative Commons license and your intended use is not permitted by statutory regulation or exceeds the permitted use, you will need to obtain permission directly from the copyright holder.
To view a copy of this license, visit https://creativecommons.org/licenses/by/4.0/

Answer letter

We would like to thank the reviewers for their positive feedback, valuable inputs, and constructive criticism. The raised points helped to improve our manuscript significantly with the addition of more experimental data that support the claims made in our manuscript. Please see below a detailed response and explanation for each point raised by the reviewers:

Reviewer #1 (Remarks to the Author):

Summary:

Haloferax volcanii is a polyploid archaeon with a main chromosome and three secondary chromosomes maintained at high copy number depending on the growth phase. Due to this high copy number, genomic segregation thus far has been considered to be random. Authors have successfully identified a two-gene operon and characterised the genes, hpaA and hpaB, with a role in segregation and equipositioning of the chromosome replicons. The study is focusing specifically on the secondary chromosome, pHV3. pHV3 encodes all genes necessary for xylose uptake and metabolism, and deletion of either hpaA or hpaB causes severe growth defects on xylose. In the HpaAB system, HpaA is a ParA-like ATPase that forms an oscillating gradient in the cytosol, and HpaB is a novel archaeal DNA-binding protein. HpaB forms discrete intracellular foci via a ribbon–helix–helix motif; mutation of a conserved tyrosine residue abolishes foci formation and pHV3 maintenance. Loss of hpaB leads to reduced pHV3: main chromosome ratio, indicating a defect in segregation and severe growth defect on xylose. In the absence of HpaA, HpaB foci become numerous and randomly distributed, indicating its importance in an orderly, equidistant positioning of HpaB-bound plasmids.

Together, these findings demonstrate that the HpaAB system functions as an equipositioning system instead of a classical ParAB system. The HpaAB system bridges bacterial ParABS and McdAB-like positioning systems, illustrating how horizontal gene transfer and archaeal-specific adaptations can give rise to hybrid mechanisms. This study provides first evidence of

an active organisational system (albeit currently demonstrated for pHV3) to be in place in the highly polyploid H.volcanii.

Here are some suggestions that could aid in the flow and readability of the paper:

Major suggestion :

1. Given that cell morphology and ploidy in H. volcanii vary significantly with growth phase, it is important to specify in the figure legend/result section the growth stage at which fluorescence microscopy data were collected.

The OD values at with the images were taken have now been indicated in figure legends of all appropriate figures.

1.1: Were wild-type and mutant cells imaged at identical time points? If so, presenting representative cells with comparable morphology across panels would improve clarity and facilitate interpretation of the localization data (Figure 2a).
If not, differences in cell shape may confound interpretation of the localization patterns (2b). Especially since line 356 mentioned HpaA oscillation being predominant in rod shaped cells. However, the mutants shown in the panel have no rod cell representation.

Originally, the cells were imaged at OD ~1.0. We have since analysed the different variants at OD 0.02 (as now indicated in the figure legend 2b).

We agree with the reviewer that interpretation of localization patterns is difficult to explain due to a difference in cell shape at the same stage of growth. To tackle this, we have used signal heterogeneity as a comparative metric to show different *in vivo* behaviour of HpaA. Furthermore, imaging at OD 0.02 enabled us to compare relatively similar cell morphologies. We thank the reviewer for this comment.

o 1.2: A supplementary video is provided for HpaA, are comparable movies available for all mutants? Alternatively, a cumulative kymograph summarizing multiple cells would be more representative than a single-cell examplesingle-cell example

We now provide time-lapse image montages for all mutants (Supplementary Figure 2). A cumulative kymograph would be more appropriate if we had observed synchronized oscillations and comparable cell dimensions across the population; however, this was unfortunately not the case. Consequently, we consider representative single-cell examples together with the newly added montages to provide the most faithful visualization of the observed dynamics. We also provide new kymographs that cover a longer time period.

2. The brightness/contrast used for the images in Figure 2a appears to be different for every panel. Using the consistent settings would allow readers to visualise the difference in fluorescence intensity better.

We thank the reviewer for pointing this out. We found the reason to be the format in which the figures were saved. We have imaged all mutants from each fluorescent protein at the same exposure time, and we have adjusted the contrast for each image to the same values for representation. Furthermore, fixing the formatting should now resolve any apparent differences in image contrast.

3. Page 14, lines 391-394 and Figures 2 and 3: While Figure 3 clearly shows Foci formation, the same observation is less apparent in Figure 2. The HpaA-msfGFP signal in Figure 2 looks compartmentalised /diffused rather than as focal points in the cells. This may reflect imaging conditions, growth stage, or contrast settings and should be clarified.

We thank the reviewer for bringing this up. The reason for this was the difference in expression levels of the protein. Figure 3 was originally imaged after induction with 1 mM

tryptophan to highlight the loss of foci formation in the absence of HpaB even with high levels of protein.

However, we have since titrated the induction with tryptophan for complementation via HpaA when grown on xylose (Supplementary Figure 5). All HpaA images are induced henceforth with 30 uM tryptophan. Furthermore, we have indicated in the text the reason for stronger foci formation in the dual-expression strain as a consequence of higher induction by tryptophan.

4. page 17, line 491-492, the sentence describing the growth defect should already be clarified here already.

We assume the reviewer is referring to the incomplete complementation by HpaA. We have revised the text accordingly, and the inclusion of the titrated HpaA condition, which shows complete complementation, should now clarify this point.

5. page 17, line 500, "..that the concentration of HpaA in the cell.." The sentence would require further experimental evidence as support, or should be rephrased

We agree with the reviewer that we cannot make conclusions about the absolute concentration of HpaA within the cell. However, to assess the influence of varying HpaA levels, we titrated the induction of HpaA and HpaA-msfGFP in Δ*hpaA* cells when grown on xylose (Supplementary figure 5). We assume different induction levels demonstrate the effect of difference in relative intracellular concentration of HpaA via expression, and show that strength of complementation indeed depends on the expression level.

6. HpaB is suggested as the primary driver of the pHV3 segregation. In Δ hpaA, HpaB foci distribution is affected, which in turn affects the segregation. Then how is it that the Δ hpaA is more viable (as seen in the glucose growth curve, Supp 2, and qPCR data, Fig 4?

We agree with the reviewer that this was surprising on first glance and this had us stumped as well. The improved growth curves on glucose show a relatively minor but consistent difference between Δ*hpaA* and Δ*hpaB* (Supplementary figure 5). Further, we repeated the qPCRs with the deletion strains harbouring the empty plasmid to keep the conditions of growth consistent for all strains, and we now observe a decrease in the pHV3 ratio in Δ*hpaA* cells, too (Figure 4 c). Nevertheless, HpaB is the trans-acting cargo specific binder for pHV3 and its loss results in a consistent and robust defect in growth. On the other hand, since the cellular function of HpaA is strictly concentration dependent, the complementation in the qPCR loses its robustness.

o 6.1: if available, is there data to show redundancy between the HpaA protein (line 741)

For this paper we have used phylogeny to highlight similarities between HpaA and other ParA like proteins in *H. volcanii*. For example, in Figure 1 we highlight that all the HpaA proteins in *Haloferax* contain P-loop walker motifs. But we understand that this statement might need more experimental data. Owing to the results we have now, we have decided to remove this statement from the manuscript. The functional analysis of the other HpaA proteins in *H. volcanii* is a separate story and subject of further work.

7. Figures 6 and 7 can be potentially unified, and certain data can be moved to the supplementary section to improve the flow of the paper.

We thank the reviewer for this suggestion. We have unified the foci number, and the G function for distribution of foci, and have moved the rest to the supplementary section.

8. Page 24, lines 725 to 726, the discussion shifts from xylose-based experiments to glucose-based observations. Consider restructuring this section so that the rationale provided on line 730 is introduced earlier.

We have restructured the section to now reflect the qPCR data and should now read better.

9. For all the fluorescently tagged proteins used in the study, a complementation assay would strengthen confidence that the tagged proteins are functional.

We agree with the reviewer, and thank them for this suggestion. We performed complementation assay upon growth in xylose and observed that while HpaA-msfGFP is able to fully complement the phenotype, HpaB-msfGFP does not complement it. This allowed us to construct the msfGFP-HpaB strains which do complement the growth phenotype on xylose, and repeat the foci distribution experiments, including almost all of the variants. Interestingly, despite the difference in growth complementation, the *in vivo* behaviour of HpaB remained quite consistent with what we had observed before. We have updated the entire manuscript to incorporate the data generated with the fully functional fusion proteins (Figures 6, and 7). We also kept the original data in the supplement (Supplementary Figures 9, 10, and 13), indicating the robust localization and dynamics of the HpaA/B fusions.

Minor suggestion :

10. Page 4, line 98: reference 52 doesn't mention histones in H. volcanii

We have added an appropriate reference, and reframed the sentence.

11. Page 4, line 98: "…H. volcanii, coincides with the presence of histones 52, facilitating DNA compaction" DNA compaction has not yet been shown by the histone in H. volcanii; therefore, consider reframing the sentence. For reference: 10.1186/s13059-023-03095-5

See point above

12. Page 5, line 108: "..chromosome (1:1 to 1:1.2), remains poorly understood" Reference?

We now added an appropriate reference.

13. Page 5, line 123: Reference? "rods in early growth phases, and plates in late growth phases"

We now added an appropriate reference.

14. Page 14, line 394: "..absence of HpaA,.." in the absence of HpaB?

We have corrected this now

15. Page 18, line 528, Full length HpaB-msfGFP instead of Wild type.

We have changed wild type to full length when mentioning the Trans induced proteins

16. Page 18, line 530, authors show mutation of Y74A disrupts foci formation ability of HpaB, in the result 5b panel 3 the image has been shown in high contrast which could be due to the high background signal. As a confirmation of expression and to verify the expression levels, western blot for the tagged mutants could be performed. The contrast and brightness of these panels should be maintained (similar as point 2)

Please see the response to point 2 above.

Further, to show that the expression is not affected, we have also included a panel of H26 cells with the empty pTA963 plasmid imaged in the same conditions as the other strains. There is

a noticeable decrease in fluorescence except the background. Furthermore, even if we do not rule out the possibility of differences in expression levels, since HpaB is a site-specific DNA-binding adaptor, the *in vivo* behaviour in the form of focus formation should remain independent of the total protein concentration in the cell, and rather depend on the local concentration around the *hpaS3* motif.

17. Page 22, line 653, "..likely adaptation to the hypersaline.." Since this is known for Haloferax proteins, please add a reference.

We have now added references

18. Page 24, line 730 should also be mentioned in the results section already, for readers to understand the flow and reasoning to go from one experiment to another.

With the restructured section, this statement loses its purpose so we have removed it. Instead the methods section clarifies the carbon source used for each experiment.

19. Page 25, line 741 "..redundancy between the HpaA proteins encoded in the H. volcanii genome" HpaA-like proteins? Since the other 3 genes are yet to be characterised? If not homologs, a justification for the sentence could provide more insight.

All HpaA proteins encoded on the secondary chromosomes are homologous ParA-like proteins. Although the three other proteins have not been characterized, they are referred to as HpaA proteins based on their sequence homology within the ParA family. Phylogenetic analysis suggests different origins with instances of independent horizontal gene transfer involving bacterial ParA-like proteins since they cluster with different bacterial ParA-like proteins rather than forming a monophyletic group (Figure 1b). Once characterized, one could mention HpaA with numbers for the secondary chromosome it is encoded on, for example HpaA3 for pHV3, to avoid ambiguity.

20. Ensure all microscopy images include both strain and fusion protein labels; avoid using "WT" to refer to proteins (e.g., use HpaA–msfGFP instead of WT in Figure 2a).

We have amended that for all figures.

21. While in Line 287 p-values have been mentioned, in all figures with statistics, these values should also be specified in the legend and ensure to keep the numbers consistent throughout. Use "ns" instead of "-"

Figure legends with significance values have been updated. Moreover, we have also added the significance values and effect sizes in supplementary tables.

22. Please ensure to maintain the figure legends for every figure and the x- and y-axis labelling for graphs.

We have crosschecked this once more, thank you

23. Statistical comparison should be shown of each mutant and complementation with respect to WT (H26). Please mention the gene/protein complemented in the figure itself (example for gene- ΔhpaB::hpaB, if the complementation was without any tags)

We show the statistical comparison with respect to H26 now, further comparisons have been listed in supplementary. The labels have also been updated.

24. Figure 4c should come before Figure 4b. This would also make sense for the flow of the paper.

We agree with the reviewer, and have updated the figure.

25. Figure 4b legend for the growth curve has Δ OD.

We have corrected the label.

26. Figure 5c, same for statistics as mentioned for Figure 4.

We have added the comparisons in the supplement with a statistics table.

27. Keeping HpaA oscillatory behaviour in mind, is it possible that the qPCR results would have a difference when cells were collected from an earlier log phase (0.01-0.06), where more rods are observed, rather than the OD600 of 0.1(Page 9, line 254)?

We thank the reviewer for this point. However, HpaA oscillation is observed in rod-shaped cells and these represent only a small fraction of the population even at earlier growth phases. Therefore, sampling at different OD values is unlikely to substantially change the population-level qPCR result. Since our analysis aims to assess global pHV3 copy number changes in the absence of HpaA, we consider the presented OD600 = 0.1 data representative and did not perform additional measurements at earlier growth phases.

28. Colours used in figure 7a,b are very similar to 7c, even though the data being shown is different. Additionally, a label for WT and mutants needs to be added to the figure.

We have added a scale bar for the heatmaps, and ensured correct labelling.

29. Figure 7c needs to have the signal density scale bar.

We have added a scale bar for the heatmaps

30. Supp Figure 2: Complement the growth curve with cell shape images to follow phenotypic changes or a spot assay?

We thank the reviewer for the suggestion. However, we do not believe that cell shape images would provide meaningful additional information. Cell morphology in *H. volcanii* is inherently heterogeneous, and representative images capture only a small subset of cells that may not reflect the full population. Since the subtle morphology phenotype is not central to the conclusions of this study, we consider the growth curves to be the most appropriate readout for complementation and therefore did not include additional morphology images or a spot assay.

31. Live cell imaging with fluorescently tagged proteins and DNA dye to have visual evidence of the said segregation defect could add to the results already presented.

We agree with the reviewer that live imaging of the segregation defect would provide valuable visual evidence of the segregation defect. We have explored this approach, but it has multiple technical limitations:

First, the dual expression strain needs strong induction to achieve detectable levels of both fluorescent proteins simultaneously, which is not ideal for proper HpaA functioning. Second, under these conditions, repeated exposure to different excitation wavelengths during time-lapse imaging results in phototoxicity which has adverse effects on cells growth and viability. We also currently lack a viable DNA stain for prolonged live-cell imaging. An alternative could be fluorescent tagging of nucleoid associated proteins Importantly, even with nucleoid staining, we would not necessarily observe the segregation defect because pHV3 makes up

less than 10% of the total DNA. Any changes in pHV3 segregation would not produce an obvious effect on overall nucleoid signal.

Due to these reasons, it is currently not a feasible approach for reliably visualizing the segregation defect.

32. Although structural prediction and mutational analysis strongly suggest DNA binding by HpaB, direct biochemical evidence (e.g., EMSA, MST, Mass photometry) is missing.

The ChIP-seq now provides solid evidence of HpaB binding directly to pHV3 (Figure 5). The mechanism of interaction *in vitro* is something we are interested in and intend to follow up on.

While the study is missing some validations, the authors have integrated several techniques to cross-validate hypothesis and conclusions, with clear evidence of the function of the two previously uncharacterised proteins. The study is not only relevant for the field but also advances our knowledge of the genome organisation in the H.volcanii.

We thank the reviewer for their suggestions and their support.

Reviewer #2 (Remarks to the Author):

Review of Premrajka et al., “Equipositioning of Chromosomes in the Polyploid Archaeon Haloferax volcanii“

This manuscript describes experiments aimed at understanding chromosome (technically secondary chromosome) segregation in the Archaeon Haloferax volcanii. Based on similarity to bacterial DNA-partitioning protein ParA, the authors identify a gene, which they call hpaA, on one of the secondary chromosomes of Haloferax (pHV3). An adjacent (partially overlapping) gene, hpaB, is uncharacterized but the authors speculate that HpaA and HpaB might cooperate to promote DNA segregation by a mechanism similar to that of ParABS

systems in bacteria. By fluorescence microscopy, fluorescent HpaA fusion proteins form discrete foci which, in rod-shaped cells, appear to spatially oscillate. HpaB forms foci that co-localize with HpaA, and loss of HpaB disrupts focus formation by HpaA. In addition, loss of HpaB (but not HpaA) skews the ratio of the secondary chromosome, pHV3, to the primary chromosome, suggesting a direct role in secondary chromosome maintenance. Cells lacking HpaA increase in size and contain more numerous HpaB foci. While the work is interesting and suggests that HpaAB may function in a similar manner to ParABS DNA segregation systems this conclusion is not well supported by the experiments presented.

Major points:

1. The title is misleading because the manuscript does not show any images of chromosome movement in live cells that prove HpaAB is required for equal distribution of chromosomes among daughter cells during division. Conclusions regarding chromosome organization are largely inferred from protein localization patterns rather than direct observation of chromosomes. The authors should either demonstrate equipositioning of chromosomes or change the title.

We agree with the reviewer that our original conclusions were based primarily on localization analyses of HpaB and did not include direct live-cell visualization of pHV3 segregation. To address this concern, we have now strengthened the manuscript by performing ChIP-seq analysis demonstrating that HpaB binds highly specific to pHV3 (Figure 5). Importantly, we identified a consensus HpaB binding sequence, termed *hpaS*, which occurs exclusively on pHV3 (Figure 5b inset).

These findings provide strong evidence that the observed HpaB foci directly mark pHV3-associated complexes rather than nonspecific cellular structures. Importantly, this organization closely parallels the well-established ParB–*parS* situation, in which ParB foci are widely used as proxies for chromosome or plasmid positioning and segregation. Therefore, the HpaB–*hpaS* interaction strongly supports our interpretation that HpaB participates in pHV3 organization and positioning.

Direct visualization of pHV3 segregation in living cells remains technically challenging because pHV3 constitutes less than 10% of the total cellular DNA. Nevertheless, we believe that the

combination of specific HpaB binding to pHV3, the identification of dedicated *hpaS* sites, and the focus-forming behaviour characteristic of HpaB provides sufficient support for the conclusions reflected in the title.

2. Fig. 2 presents localization data and evidence for oscillatory behavior of HpaA. However, it does not provide direct evidence for active or directed chromosome movement. It is unclear whether HpaA interacts nonspecifically with DNA, binds specific chromosomal regions, or associates with other segregation factors. The authors should include a panel of images showing chromosomal DNA (e.g., nucleoid staining or specifically labeled chromosomal loci) to contextualize the observed protein dynamics. Additionally, it would be great for the authors to comment on whether differences in localization patterns could arise from cell-cycle heterogeneity, as cells do not appear to be synchronized. Clarification on whether nucleoid compaction differs between wild-type and HpaA mutant strains would further strengthen the interpretation.

We thank the reviewer for these constructive suggestions. We have now added line-scan analyses of representative cells demonstrating that nucleoid compaction is not detectably altered among the different HpaA variants. These data clearly argue against major differences in global chromosome organization between wild-type and mutant strains.

We also agree that information on HpaA DNA interaction would be highly desirable. During the revision we now succeeded in HpaA purification and therefore could further address whether HpaA interacts with DNA. To this end, we have additionally included EMSA experiments demonstrating ATP-dependent HpaA–DNA interactions. While these experiments do not yet distinguish between sequence-specific and nonspecific DNA binding, they provide direct evidence that HpaA can associate with DNA.

We also considered the reviewer’s concern regarding possible cell-cycle heterogeneity. To minimize variability arising from growth state differences, fluorescence localization patterns were re-examined in cells at OD_{600} 0.02, where cultures display a significantly higher

proportion of rod-shaped cells. The comparison has now been quantified and included in Figure 2d, and Supplementary Figure 3.

We agree that dissecting the precise molecular mechanism underlying HpaA dynamics and its potential interactions with segregation factors will be highly interesting. However, we believe that these mechanistic questions extend beyond the scope of the current study, whose primary aim is to establish the role of the HpaAB system in pHV3 organization and positioning.

3. In Fig. 3 the altered HpaA dynamics observed in the absence of HpaB resemble those reported for ATP-binding or ATP-hydrolysis defective HpaA mutants. This raises the possibility that HpaB influences HpaA ATPase activity or stabilizes a specific nucleotide-bound state. The authors should comment on this possibility and (even better) perform in vitro experiments to directly test whether HpaB alters HpaA ATPase activity.

We absolutely agree with the reviewer and have ourselves looked into the possibility of HpaB affecting HpaA ATPase cycle. However, after multiple attempts under different conditions, we haven't found any conclusive evidence of ATPase activity in vitro, yet. We assume this is owing the complexity of the mixture which needs to be performed in a buffer with perfect ionic strength, in the presence of HpaB, and the *hpaS3* motif. We cannot rule out the possibility that we do not have the right conditions for the ATPase assay since the ATPase standard curve seems to suffer in high salt as well. We also mention this in the text now.

Therefore, we have focussed our claims on in vivo behaviour of HpaA, and also mentioned the possibility of ATPase activity dependent on DNA and HpaB interactions. Dissecting the ATPase activity of HpaA goes beyond the scope of this paper and we would like to perform these assays together with HpaA mutants once we have established a robust in vitro system for ATPase activity measurements under high salt conditions.

4. On l. 469 the authors claim that, "HpaA and HpaB are essential for growth on xylose," This is clearly an overstatement! HpaA and B mutants exhibit growth defects but loss of either protein is clearly not lethal. This statement should be amended to something like: "HpaA and

HpaB are both necessary for optimal growth on xylose”. Apart from xylose, did the authors get to see any growth defect with other common sugars like glucose? Did the authors test the double KO strain with both hpaA and hpaB deletion? Are they lethal?

We agree with the reviewer and have amended the statements accordingly.

5. The data in Fig. 5 should include DNA colocalisation as a control since protein foci can arise from aggregation, oligomerization, and/or phase separation. Without seeing the DNA, it is difficult to conclude that the observed foci represent chromosome-associated structures.

We agree that fluorescence foci of HpaB alone might not necessarily demonstrate chromosome association. However, as described in our response to point 1 above, we now show by ChIP-seq that HpaB specifically binds *hpaS* sites that occur exclusively on pHV3, strongly supporting that the observed foci are chromosome-associated rather than nonspecific aggregates. In addition, the msfGFP-HpaB fusion complements growth on glucose, demonstrating that the fusion protein is functional (Supplementary figure 5f).

6. Also, related to Fig. 5 the authors claim that HpaA binds DNA but never demonstrate this fact. Analogy with ParA and AlphaFold predictions are not sufficient. At the very least, the authors should perform electrophoretic mobility shift analysis (EMSA) using purified HpaA before they say that it binds DNA.

We have now purified HpaA, and performed High salt EMSAs in acrylamide gels and show that HpaA binds DNA in the presence of ATP (Figure 2a).

7. In Fig. 6 no biochemical evidence is provided for a physical interaction between HpaA and HpaB. Also, without DNA colocalisation data it is impossible to determine whether HpaA controls HpaB distribution on DNA or whether loss of one protein alters localization of the other via more generic perturbations of cellular organization.

While we agree that direct biochemical interaction would strengthen the claim, multiple different experiments from our data independently show that the presence or absence of each protein has an effect on the in vivo behaviour of the other. For HpaA we show that the lack of HpaB changes focus formation, oscillation, and overall dynamics (via SPT). For HpaB, we show that the lack of HpaA leads to random localization of the foci. However, most important, an N-terminal truncation of HpaB leads to loss of ordered HpaB foci distribution that is most similar to a deletion mutant. In summary, while not constituting direct physical proof, the described experiments strongly hint towards an HpaAB interaction in vivo.

Furthermore, the proteins are part of an operon with an overlap, both show consistent growth and qPCR defects related to xylose metabolism, and pHV3 copies.

We hope that this reviewer can agree with us that these data are sufficient to support our claim of in vivo HpaA-HpaB interaction.

8. In Fig. 8, again conclusions regarding interactions between N-terminally truncated HpaB and HpaA are based on localization data (and analogy to bacterial ParAB systems). Biochemical or biophysical interaction assays are required to distinguish between a true loss of interaction and indirect effects on protein localization or stability resulting from N-terminal deletion. The AlphaFold analysis is suggestive but not definitive. None of the experiments in the paper rule out the possibility that HpaA and HpaB simply form aggregates or phase condensates, possibly induced by GFP labeling.

Please see our response to point 7. Further, both tagged proteins complement the growth phenotype on xylose, showing that they are functional in vivo. We also show that an N-terminal GFP fusion of HpaB still forms foci, arguing against GFP-driven aggregation.

Together, the different truncation and mutant analyses provide consistent genetic evidence for a functional HpaA–HpaB interaction rather than nonspecific aggregation or phase separation.

We agree that in vitro biophysical interaction assays would be interesting. However, these are technically challenging under halophilic conditions and would not substantially change the conclusions of the study at this point.

9. Conceptually, I am having a hard time understanding how inhibition of an equipartitioning system results in population-level depletion of the secondary chromosome pHV3. If the partitioning is simply unequal then the average should stay the same (at least for a while) but the variability in pHV3 ratio in the population should dramatically increase. If the secondary chromosome pHV3 is important for growth and survival I would expect cells with a lower copy number of this chromosome to be at a competitive disadvantage and that the ratio of pHV3 to primary chromosome might actually increase. I would expect the observed result only (or primarily) under conditions where increased pHV3 copy number is deleterious.

We agree with the reviewer that inhibition of an equipartitioning system should primarily increase variability in pHV3 inheritance rather than directly changing the average copy number. Our interpretation is therefore that HpaAB functions in pHV3 equipartitioning, ensuring low-variance segregation at cell division. When this system is disrupted, pHV3 distribution becomes more stochastic, leading to heterogeneous copy numbers in new-born cells.

The population-level shift we observe is then not a direct consequence of partitioning itself, but rather reflects selection acting on this heterogeneity over multiple generations. Depending on growth conditions, cells with suboptimal pHV3 copy numbers are at a disadvantage, which results in a change in the steady-state ratio of pHV3 to the main chromosome.

We therefore interpret the phenotype as a combination of segregation defects and growth-dependent selection acting on the resulting distribution.

10. On ll. 504-506 the following sentences are not accurate: “Moreover, these defects could be rescued upon tryptophan-controlled expression of HpaA and HpaB via ectopic complementation constructs. Complementation of hpaB fully restored growth to wild-type levels.” The modest increase in cell density early in the growth curve for HpaA complementation does not appear to warrant the word “rescue”, especially given that the

authors characterized the hpaA knockout cells as inviable.

We now added new data, showing that inducer titration can find optimal complementation conditions for HpaA (Figure 4, Supplementary figure 5 b,c). We have changed the text accordingly.

11. Overall, the paper lacks sufficient statistical analysis and/or rigor. For example, in Fig. 2 how often did the authors observe oscillation of HpaA? What fraction of cells had diffuse localization or non-oscillating puncta? How many biological replicates were performed? How many cells analyzed from each replicate? This information should be provided for every piece of quantitative data.

We now mention these statistical data in the figure legends, and a have also added all statistical tables in the supplementary section.

Minor Points:

1. Overall the figures should be labeled much better. For example, in Fig. 2 the authors should make clear that the individual kymograph images in 2b correspond to analysis of movies containing the images above, in 2a. At least I assume that they do. Also they should label on the figure which fluorescent fusion protein we are seeing.

We thank the reviewer for the suggestions. We have changed the figures accordingly

2. In Fig. 2 the authors should mark which cell(s) in the image were used for the kymograph analysis. They should also mark the line along which the kymographs were computed. Also, the kymographs are not super convincing. The authors should include sequential images from the time laps movies illustrating the oscillatory behavior.

We have now added time lapse images in the supplementary section along with their kymographs.

3. For the rescue and tryptophan-induced expression experiments the authors should show immunoblots of the expression levels. For example is the lack of "rescue" by HpaA simply due to low expression levels in those experiments?

We titrated HpaA expression using different tryptophan concentrations and found that complementation of the Δ*hpaA* phenotype is indeed dependent on expression level. At the right inducer concentration, the deletion phenotype can be fully rescued.

This indicates that the previously observed lack of rescue is due to insufficient expression rather than loss of HpaA function. Because complementation can be achieved reproducibly by tuning inducer levels, we did not include western blots.

4. In Fig. 4 I cannot tell whether the statistics were performed on biological or technical replicates.

We have now added the statistical data in the figure legend and also in the supplement. For the qPCRs the statistics were performed for all biological replicates together.
